# Phytochemical Assessment of Native Ecuadorian Peppers (*Capsicum* spp.) and Correlation Analysis to Fruit Phenomics

**DOI:** 10.3390/plants9080986

**Published:** 2020-08-04

**Authors:** Carlos A. García-González, Cristina Silvar

**Affiliations:** 1 Grupo de Investigación en Bioloxía Evolutiva, Departamento de Bioloxía, Universidade da Coruña, 15071 A Coruña, Spain; cgarcia@utmachala.edu.ec; 2 Facultad de Ciencias Químicas y de la Salud, Universidad Técnica de Machala, El Oro 070150, Ecuador

**Keywords:** nutraceutical compounds, capsaicinoids, ascorbic acid, polyphenols, Tomato Analyzer, conventional phenotyping, correlation analysis

## Abstract

In this work, the impact of pepper (*Capsicum* spp.) fruits morphology on their composition for health-promoting compounds was investigated. For that purpose, pepper accessions from Ecuador, one of the hotspots in *Capsicum*’s origin, were analyzed for ascorbic acid, polyphenols, capsaicinoids, and prevention of cholesterol oxidation. Plant and fruit phenomics were assessed with conventional descriptors and Tomato Analyzer digital traits. Significant differences among accessions and species revealed a large diversity within the collection. The *Capsicum frutescens* group displayed the highest levels of capsaicinoids, whereas the polyphenols shortly varied among the five domesticated species. *Capsicum pubescens* exhibited the lowest content of ascorbic acid. The conventional descriptors describing the magnitude of plants and fruits, as well as digital attributes under the categories of size, shape index, and latitudinal section, mostly explained the variance among *Capsicum* groups. Correlation test revealed that phytochemical components were negatively correlated with the morphometric fruit attributes, suggesting that huge fruits contained lower amounts of nutraceutical compounds. Multivariate analysis showed that parameters related to fruit size, shape, and nutraceutical composition primarily contribute to the arrangement of pepper accessions. Such results suggested that those traits have been subjected to higher selection pressures imposed by humans.

## 1. Introduction

Peppers (*Capsicum* spp.) are one of the most important vegetables worldwide [1,2]. The genus belongs to the Solanaceae family and currently harbors more than 35 species, five of which (*Capsicum annuum* L., *Capsicum chinense* Jacq., *C. frutescens* L., *Capsicum baccatum* L., and *C. pubescens*, Ruiz et Pav.) are domesticated, and exemplify the most relevant *Capsicum* in terms of nutritional value, economic importance, and breeding [2]. The first three species are included in the Annuum complex and are potentially crossable with ease [3]. The other two belong to the Baccatum and Pubescens complexes, respectively, and they possess low compatibility with the first complex [4].

The genus *Capsicum* is native to South America [5]. Recently, Carrizo-Garcia et al. [6,7] proclaimed the genesis of this genus in a broad area, including Peru, Ecuador, and Colombia, along the Andes in western–north-western South America. The five cultivated *Capsicum* species were independently domesticated in either Mesoamerica or South America and subsequently shifted to the primary centers of diversification, mostly comprised of the Andean regions [5]. After the 15th century, peppers became grown worldwide, suffering from additional diversification at the secondary centers, which resulted in thousands of landraces or native varieties, well adapted to specific agro-climatic conditions [8]. Although landraces or traditional pepper varieties exist worldwide, those coming from the primary centers of crop diversity, such as Ecuador, might preserve even more promising variability and valuable traits [9].

Collections of native landraces have been widely investigated at the DNA level with molecular markers [10,11,12]. Nonetheless, highly precise phenotyping based on objective scales is much more limited. Historically, pepper morphological characterization has been performed with conventional heritable descriptors based on manual and visual inspections, which can be strongly biased [13,14]. High-throughput automated phenotyping tools are being continuously developed [15]. Among them, Tomato Analyzer (TA) software has emerged as an extremely useful system to perform accurate phenomics in Solanaceae. This freeware allows the semi-automatic and highly precise scoring of a large number of quantitative fruit traits from scanned images of fruit sections [16,17]. It has been successfully employed for tomato [18,19,20], eggplant [21,22], and pepper [23,24,25,26] and the diversity measured by fruit TA parameters demonstrated to be higher than that reflected by conventional descriptors [20,25,27]. Apart from the importance of adequate phenotyping, the increasing interest of consumers for tastier and healthier foods, make it necessary to investigate pepper landraces for the bioactive composition of fruits [28]. As nicely reviewed elsewhere, *Capsicum* fruits are a rich source of secondary metabolites with nutraceutical properties, including capsaicinoids, ascorbic acid, and phenolic compounds [2,29]. Capsaicinoids are alkaloids, responsible for the pungency in the genus *Capsicum*. Capsaicin (Cap) and dihydrocapsaicin (DHCap) are the major components of this group, besides other minor capsaicinoids, such as nordihydrocapsaicin (NDHCap), homocapsaicin (HCap), and homodihydrocapsaicin (HDHCap) [30]. Ascorbic acid (Vitamin C) acts as a cofactor for enzymes involved in human metabolism [31]. This vitamin can appear at outstanding levels in pepper fruits and its content increases during fruit ripening [32]. Phenolic compounds (or polyphenols) are defined as molecules that contains at least one aromatic ring attached to one or more hydroxyl groups. Flavonoids, phenolic acids, and their derivatives are the principal polyphenols in peppers [33]. These phytochemical components from pepper species are known for their analgesic, anti-obesity, cardio-protective, pharmacological, neurological, and dietetic properties [29]. Many of these functional features are largely attributed to the antioxidant activity of capsaicinoids, polyphenols, and vitamin C, which play a crucial role in scavenging the free radicals responsible for oxidative damage [33,34]. In addition, antioxidant compounds prevent the oxidation of lipids, such as cholesterol [35,36].

The morphology and biochemical composition of pepper fruits are affected by environmental growing conditions [37,38]. Ecuador is one of the most diverse countries in the world and it is divided into four geographical regions, including the Coast (lowland regions between the Pacific Ocean and the Andes), mountains (the Andean and Austro regions), and Amazon (east of the Andes mountain range), which are characterized by distinctive and well-defined climatic parameters. Such climatic diversity allowed for extensive growth of native and exotic plant species [39,40] and may have largely influenced pepper fruit characteristics prevailing in each natural Ecuadorian region.

Several works dealing with the phenotypic characterization of diverse *Capsicum* spp. collections and their composition in human health-promoting metabolites have been conducted to date. However, both assessments are usually accomplished separately. As far as we know, there are not any reports attempting to establish correlations between the morphometric attributes of pepper fruits and their biochemical profiles for nutraceutical components. Nevertheless, this knowledge results essential, as it will strongly determine the speed at which promising traits could be integrated into pepper commercial types. The main goals of the present work were to: (i) screen a collection of *Capsicum* spp. accessions for their content in capsaicinoids, ascorbic acid, polyphenols, and capability to prevent cholesterol oxidation; (ii) phenotypically evaluate this panel with both conventional descriptors and fruit attributes obtained with the Tomato Analyzer software; (iii) identify relationships between fruit morphology and phytochemical compounds. This study was performed in landraces from different geographical regions of Ecuador, which represent one of the hotspots in the origin and speciation of the genus *Capsicum*. 

## 2. Results

### 2.1. Biochemical Analysis

Four biochemical parameters, including ascorbic acid, phenols, prevention of cholesterol oxidation, and capsaicinoids (Cap, DHCap, HCap, NDHCap, and HDHCap) (Figure S1) were assessed in mature fruits from 42 accessions. Plants from six accessions (CGN17040, PI595908, PI593924, PI585264, PI585271, PI585273) died before mature fruits could be harvested. Highly significant differences (*p* < 0.001) among accessions were detected for all of them, except for their capability in preventing cholesterol oxidation during heating (Table S1). The content in ascorbic acid and capsaicinoids explained the greatest part of the variation. The amount of that acid ranged from 107.4 to 422.8 mg/100 g fresh weight (FW), with an average value of 209.7 mg/100 g FW. The highest levels were measured in the *C. annuum* accession PI241670 and lowest in the *C. pubescens* genotype PI585265 (Table S1). The greatest capsaicinoids concentration was found in a *C. frutescens* accession (PI355808), which harbored 8.6 mg/g dry weight (DW) distributed into 72.6% of Cap and 27.4% of DHCap. No capsaicinoids could be detected in 42.8% of *C. annuum* accessions, 22.2% of *C. chinense*, 14.3% of *C. pubescens*, and 8.3% of *C. baccatum*. As expected, Cap and DHCap appeared as the most abundant capsaicinoids. Curiously, one out of seven *C. pubescens* (PI585267) displayed a capsaicinoids profile composed of 100% DHCap. Similarly, three *C. pubescens* (BGV13300, PI585269, and PI585275) and one *C. annuum* genotype (PI585238) had compositions on which the percentage of DHCap prevailed over that in capsaicin (Table S1). The average value for total polyphenols content was 24.0 mg ferulic acid equivalents (FAE)/g DW, varying from 13.8 mg FAE/g DW in PI585254 (*C. frutescens*) to 33.9 mg FAE/g DW in PI585238 (*C. annuum*). The highest percentage of remaining cholesterol (93.2%) was observed in *C. chinense* PI593933, while the lowest (46.4%) was detected in *C. frutescens* PI595907, although they were not significantly different (Table S1). Capsaicin and dihydrocapsaicin showed the highest coefficients of variation, suggesting wide variability among all analyzed pepper, from non-pungent to hot (Table S1). 

**Figure 1:**
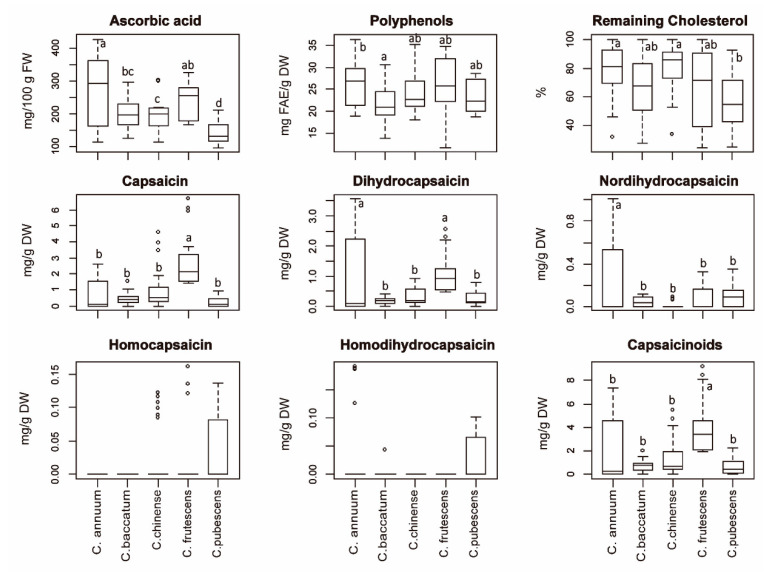
Box plot of biochemical parameters. Twenty-fifth percentile, median, 75th percentile, and range minimum-maximum. Outliers (white circle) were identified as 1.5 times the interquartile range. Different letters indicate significant differences at *p* < 0.05.

The analysis of variance, considering the species effect, indicated significant differences among *Capsicum* spp. for all biochemical measures, except for HCap and HDHCap. As above, ascorbic acid and capsaicinoids (primarily Cap) highly contributed to the differences among groups (Table 1). *C. annuum* showed the average highest level of ascorbic acid (279.8 mg/g FW), although this was not significantly different from that in *C. frutescens* (245.8 mg/g FW). The lowest amounts of ascorbic acid appeared in *C. pubescens* accessions (average of 142.4 mg/g FW) (Figure 1). The variation attributed to capsaicinoids was essentially due to the largest presence of capsaicin in *C. frutescens* in relation to other species (Figure 1). The distribution profiles, expressed as a percentage, confirmed that Cap and DHCap were the dominating capsaicinoids (Figure S2). *C. frutescens*, *C. chinense*, and *C. baccatum* primarily contained Cap; *C. pubescens* possessed mostly DHCap, while *C. annuum* exhibited an almost equal distribution for both capsaicinoids. Interestingly, accessions belonging to *C. pubescens* evidenced percentages of NDHCap, HCap, and HDHCap much higher than the other four *Capsicum* spp. (Figure S2). Only *C. annuum* and *C. baccatum*, which possessed the highest and lowest levels, respectively, displayed strongly significant differences for their content in total polyphenols (Figure 1). The lowest capacity to prevent cholesterol oxidation was observed in *C. pubescens* accessions, which significantly differed from that of the other four species.

### 2.2. Morphological Assessment with Conventional and TA Descriptors

*Capsicum* accessions were evaluated with 45 conventional descriptors (15 quantitative and 30 qualitative). Traits related to plant, leaf and inflorescence were recorded in 48 accessions while fruit traits were registered in 42 accessions. Conventional descriptors revealed great diversity within the Ecuadorian collection, with highly significant differences being observed for all of the quantitative traits analyzed in plants, flowers, and fruits (Table S2). The parameters that most contributed to explain the variance among genotypes were those related to plant morphology (plant height (PHE), plant width (PWI), and stem length (SLE)) and fruit size (fruit weight (FWE), and fruit length (FLE)). Thus, the PHE showed a mean value of 67.8 cm, ranging from 24.6 cm in *C. pubescens* accession PI585262 to 109.5 cm in *C. frutescens* PI593920. The heaviest fruits were recorded in BGV5857 (*C. annuum*) with an average value of 43.5 g per fruit, while the lightest (0.23 g per fruit) were found in *C. frutescens* PI585257 (Table S2). A coefficient of variation up to 96.2% and 91.5% was recorded for SLE and FWE, respectively, while for the remaining traits, it was lower than 56% (Table S2).

ANOVA analysis considering the *Capsicum* species suggested that belonging to a particular species greatly contributed to explain the morphological variation among accessions. Significant differences among species were found for most quantitative traits except for anther length (Table 2). Regarding plant morphological traits, *C. frutescens* accessions showed the highest PHE, SLE, and mature leaf length (MLL), while *C. pubescens* displayed the smallest PHE and MLL (Table 2). The shortest flowers were detected in *C. frutescens* and the largest ones in *C. annuum* and *C. pubescens*. A broad diversity was found for most of the fruit parameters. Remarkably, highly significant differences among the five species were identified for fruit length, with *C. frutescens* fruits being the shortest and *C. annuum* fruits the longest. A similar contrast between these two species was observed for fruit weight, with a mean difference of more than 15 g (Table 2).

The distribution of different qualitative morphological characteristics, which were measured on a scale, were represented as the percentage of relative frequencies for each group of accessions at any different region (Figure S3). The post hoc Tukey HSD (honest significant difference) test showed significant differences for all qualitative descriptors among the five species, although those most contributing to the variance were stem pubescens, corolla color, corolla spot color, anther color, and filament color (Table S3). Stem pubescens and corolla color clearly differentiated *C. pubescens* from the other species, whereas *C. annuum* showed the highest variation for anther color. Similarly, *C. baccatum* and *C. pubescens* exhibited differences in flower position (Figure S3).

Fruit morphology was assessed by image examination of scanned fruit sections with the Tomato Analyzer software. As occurred for the conventional fruit descriptors, broad diversity was found within the collection. All evaluated descriptors evidenced highly significant differences (*p* < 0.001) among average values for the 42 pepper accessions (Table S4). The greater part of variation was explained by attributes related to fruit size and shape, i.e., those under the category’s basic measurements, latitudinal section, and shape index. High F values were also observed for H.Asymmetry.Ov (HAov), proximal indentation area (PIA), and distal indentation area (DIA) (Table S4). Minimum values of zero were recorded for PIA, DIA, shoulder height (SH), distal end protrusion (DEP), Ovoid (Ov), Obovoid (Ob), H.Asymmetry.Ob (HAob), and HAov. The coefficient of variation ranged from 4.5% in Distal eccentricity (DEC) to 305.9% (HAov). For each category, the descriptors with the largest variability were: Area (88.7%), Fruit Shape Index Internal (57.9%), Distal Indentation Area (240.5%), Tomato Pericarp Area (89.7%), Curved Fruit Shape Index (61.1%), Fruit Shape Triangle (58.5%), Circular (46.2%), Proximal Indentation Area (135.0%), and H.Asymmetry.Ov (305.9%) (Table S4). 

Highly significant differences (*p* < 0.001) were detected for all analyzed TA descriptors across pepper accessions within each *Capsicum* group (data not shown), but also among the five domesticated species (Table 3). As above, the greatest contribution to the variance was due to descriptors related to size (basic measurements and latitudinal section) and shape indexes. High variation among species was also detected for attributes associated to homogeneity (Ellipsoid), shape (PIA), and Asymmetry (V.Asymmetry (VAs)). In general, the greatest differences were found between *C. frutescens* and *C. annuum*, which showed the lowest and highest values, respectively, for the majority of fruit size features (Table 3). Considering the shape, measured as Fruit shape index external I (FSIEI), Fruit shape index external II (FSIEII), Curved fruit shape index (CFSI), and Fruit shape index internal (FSII) indices, the highest and significantly different values were recorded for *C. annuum* and *C. baccatum*. This suggests that pepper fruits from those species displayed much larger height than width, whereas in *C. frutescens*, *C. chinense*, and *C. pubescens* fruits, both attributes were not so dissimilar. Likewise, *C. baccatum* accessions possessed the least triangular shape, as indicated by the Fruit shape triangle (FST) value (Table 3). The largest variability, according to the coefficient of variation, was recorded for *C. frutescens*, which displayed a CV higher than 50% for twenty-two traits (Table 3).

### 2.3. Differences Among Geographical Regions

**Figure 2:**
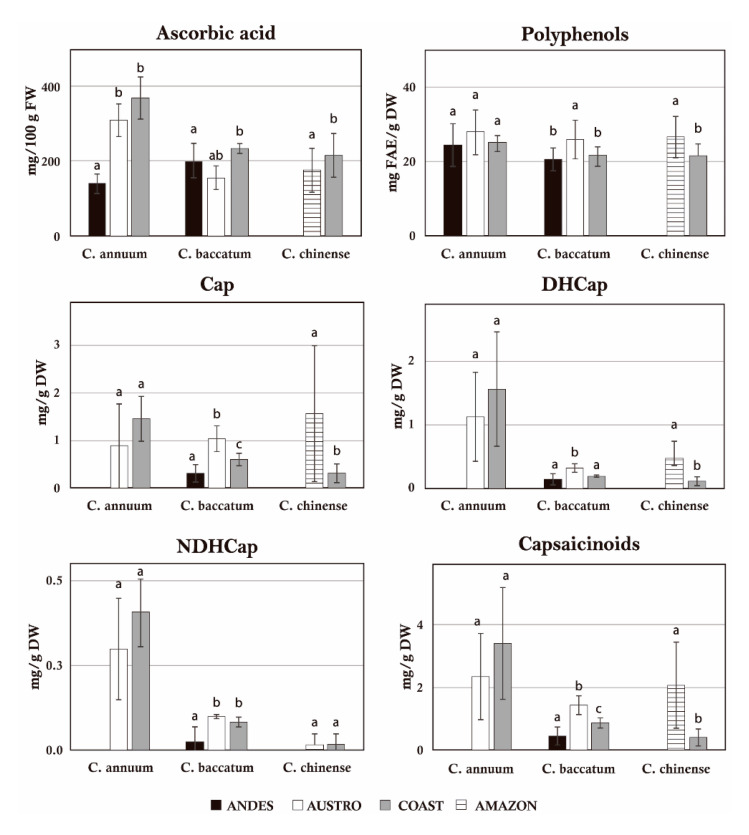
Bar plots of biochemical parameters expressed per region for *C. annuum*, *C. baccatum,* and *C. chinense*. Different letters indicate significant differences at *p* < 0.05. Only those parameters showing significant differences among regions are represented.

Differences among geographical regions were analyzed for *C. annuum*, *C. chinense*, and *C. baccatum*, which were represented by more than one accession at any of the four Ecuadorian areas (Coast, Andes, Austro, and Amazon). The analysis of phytochemical parameters per region pointed out that *C. annuum* fruits from the Coast and Austro contained significantly higher amounts of vitamin C and capsaicinoids than those originated from Andes (Figure 2). *C. baccatum* from Austro possessed the lowest amount of vitamin C but the largest significant values for phenols and capsaicinoids. Finally, *C. chinense* fruits coming from the Coast maintained higher amounts of vitamin C but significantly lower quantities of phenols and capsaicinoids than those growing in the Amazon region (Figure 2).

Regarding the conventional descriptors, significant differences were observed in *C. annuum* for the majority of traits. Accessions from the Andes possessed plants with the highest height, stem length, stem diameter, and the largest leaves. In the same manner, mountainous areas under the Andean region contained pepper fruits with the largest width, weight, and pericarp thickness but the least length (Table S5). Such associations between fruit characteristics of *C. annuum* and specific geographical regions were also evident on the sections analyzed with TA software. Thus, peppers from the Coastal region exhibited the lowest values for the majority of parameters under the size and latitudinal section categories but the largest for most of those traits belonging to shape index, homogeneity, asymmetry, and internal eccentricity (Table S5). The *C. baccatum* group from Austro showed significantly taller plants and longer leaves than those from the Coast and Andes. Significant differences for conventional fruit traits were only detected for fruit weight, with Andes being the area with the heaviest fruits (Table S5). However, analysis of data from scanned fruits revealed significant differences for the majority of attributes, with *C. baccatum* peppers from the Andes being those showing the most significant differences regarding traits related to size (maximum height (MH)) and shape (FSIEI, FSIEII, CFSI) (Table S5). In the case of *C. chinense*, significant disparities between the Amazon and Coast were observed for plant width, stem length, fruit weight, and pedicel length. Thus, accessions from the former region were wider and taller than those from the latter. Likewise, pepper fruits from the Amazon had the highest weight but shortest pedicels. The analysis of sections indicated that *C. chinense* fruits from the Amazon displayed significantly higher values for the majority of TA descriptors associated to size (perimeter (P), area (A), height mid-width (HMW), MH, curved height (CH), lobedness degree (LD), tomato pericarp area (TPA)) and shape (FSIEI, FSIEII, CFSI, FSII, distal fruit blockiness (DFB)) (Table S5).

### 2.4. Correlation between Nutraceutical and Morphological Parameters

**Figure 3:**
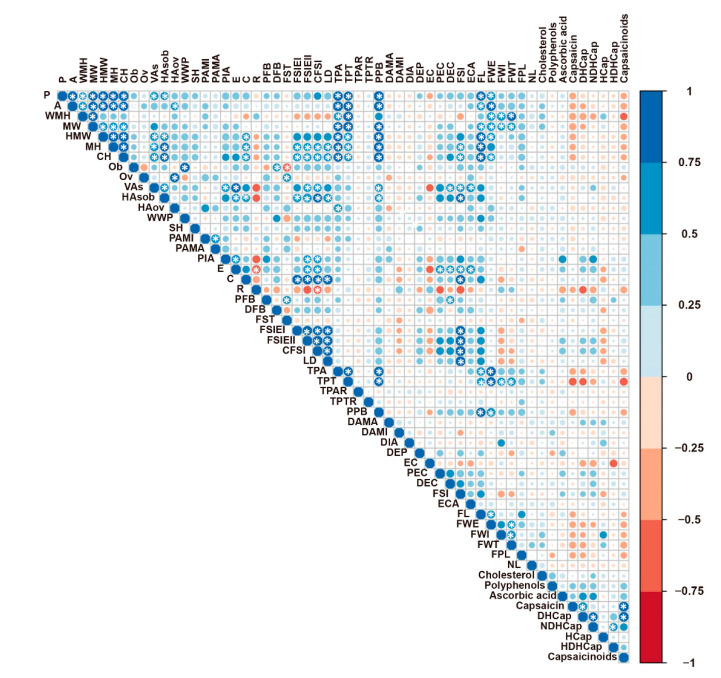
Pearson’s rank correlation coefficients between pairs of traits. Color intensity (right scale) and the size of the circles are proportional to the correlation coefficients. Significant correlations at *p* < 0.05 are indicated with a white asterisk inside the circle.

An analysis of correlation was performed with all fruit parameters to investigate the relationship between biochemical components and morphological traits, represented as conventional descriptors and morphometric attributes implemented in the TA software. Interestingly, the mean values of nutraceutical traits (except remaining cholesterol) showed a negative correlation with fruit conventional descriptors (Figure 3). Likewise, negative correlations were observed between biochemical parameters and the majority of TA measures, particularly with those under the categories of basic measurements and latitudinal section. Unexpectedly, none of these correlations were significant after Bonferroni correction for multiple testing. In the same manner, positive Pearson coefficients were recorded between pairs of biochemical parameters, although only Cap, DHCap, HDHCap, and NDHCap were significantly inter-correlated (Figure 3).

Strong positive significant correlations were observed for conventional fruit length, fruit weight, and size-related traits described by TA software (P, A, WMH, MW, HMW, MH, CH). Additionally, those conventional parameters were significantly correlated with TPA, tomato pericarp thickness (TPT), and pepper pericarp boundary (PPB) (Figure 3). The network of correlations for TA attributes revealed that most of them were rather independent, whereas some categories were tightly linked and significantly correlated. Hence, traits under the size category were positive correlated with TPA, TPT, and PPB. The shape indices were significantly and positive correlated with MH, CH, VAs, HAob, PIA, Ellipsoid, and Circular. In addition, V.Asymmetry and Ellipsoid were significantly linked to the parameters under Internal Eccentricity. Negative significant correlations were evidenced for Rectangular when compared to Ellipsoid and CFSI but also for FST and Obovoid (Figure 3).

### 2.5. Multivariate Analysis

Principal component analysis was performed with six fruit conventional quantitative descriptors related to fruit morphology, 41 TA measures, and 9 nutraceutical traits. Principal component analysis (PCA) resulted in thirteen principal components with eigenvalues > 1, cumulatively accounting for 87.6% of the total variance (Figure S4). The first component (PC1) explained 27.4% of the total variance and it was positively and robustly correlated (>50%) to all TA attributes within the size category, various under latitudinal section (TPA, TPT, PPB), and the majority of conventional descriptors (FLE, FWI, FWE, FWT). Remarkably, all nutraceutical traits, except cholesterol, ascorbic acid, and HCap, were negatively correlated to this first component, in particular Cap, DHCap, and capsaicinoids (Figure S4). The second component (PC2), which accounted for 17.4% of the variance, was positively correlated to traits related to size (HMW, MH, CH), shape index (FSIEI, FSIEII, CFSI), homogeneity (Circular), asymmetry (VAs, HAob), and latitudinal section (LD) but also to fruit length, within conventional descriptors. The third component (PC3) contributed to 8.8% of the total variance and the nutraceutical parameters were clearly the principal traits responsible for the observed variability, being phenols, Cap, DHCap, and capsaicinoids those with a higher degree of correlation (Figure S4). 

**Figure 4:**
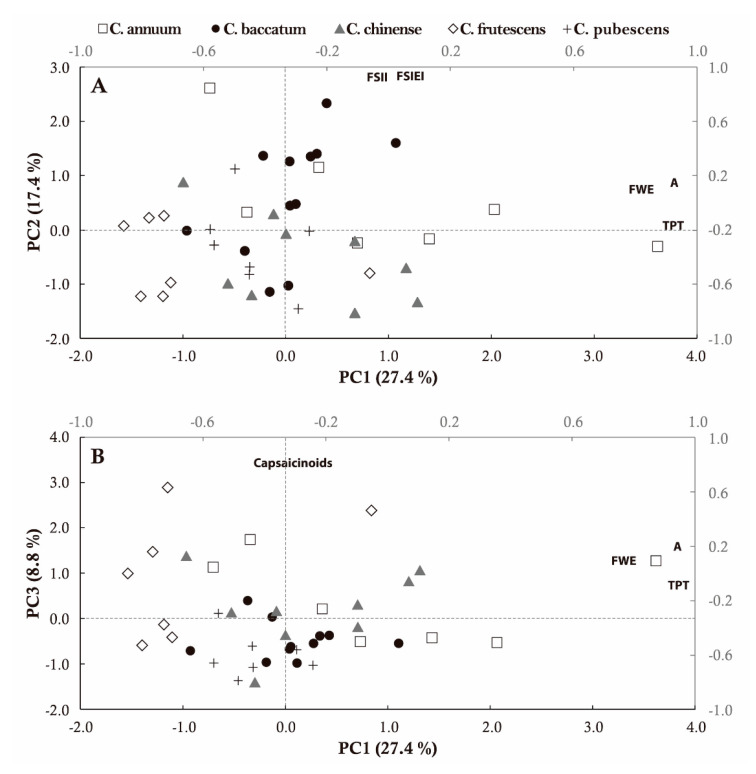
Scatter plot of the principal component analysis (PCA) analysis for forty-two Ecuadorian pepper landraces. (**A**) Principal Component 1 (PC1) vs. PC2; (**B**) PC1 vs. PC3. Secondary axes show the factor loadings values. Traits with the highest factors for each component are represented.

The three principal components were used to project the forty-two accessions on two-dimensional plots. Pepper fruits were widely dispersed on the PCA diagrams according to their morphology and nutraceutical composition (Figure 4). Area, TPT, and TPA with correlation coefficients higher than 90%, explained the distribution of the accessions on the first axis. Three fruit shape index traits (FSIEI, FSIEII, FSII) and LD were the main factors discriminating the pepper fruits on the second component. The arrangement of accessions on the third axis, primarily responded to their content in capsaicinoids (Figure 4). Belonging to a particular *Capsicum* spp. did not greatly influence the distribution of pepper accessions on the graphical space. Thus, *C. annuum* mainly plotted on the positive axes of PC1 and PC2, according to their predominant morphology, which included fruits with a large height/width ratio (average values for FSIEII of 4.93), large size (average area = 2154.82) and weight (average FWE = 19.05), and thicker pericarps (average TPT = 3.85). *C. frutescens* comprised the smallest fruits (average area = 241.20, average FWE = 2.83), with variable shapes from conical to elongated (average FSIEII = 2.12), which mainly converged on the negative panel delimited by PC1 and PC2. The majority of *C. chinense* accessions were distributed on the negative area of the first component. That position agreed with the medium sizes of these pepper fruits (average area = 1024.48, average FWE = 10.12), which tend to possess more round and conical shapes (average FSIE2 = 2.14) (Figure 4). Similarly, accessions from *C. pubescens* plotted on the negative segment of PC1 (average FSIEI = 1.96), although closer to the zero value of PC2, which is in agreement with the presence of fruits with size and shape in the midst of *C. frutescens* and *C. chinense* (average area = 609.20, average FWE = 8.34, FSIEI = 1.95). *C. baccatum* exhibited the widest dispersion, with accessions displaying positive and negative values for both the first and second components (Figure 4). The third component discriminated accessions from *C. frutescens*, *C. chinense*, and few *C. annuum*, as those harboring the highest amounts of capsaicinoids. 

**Figure 5:**
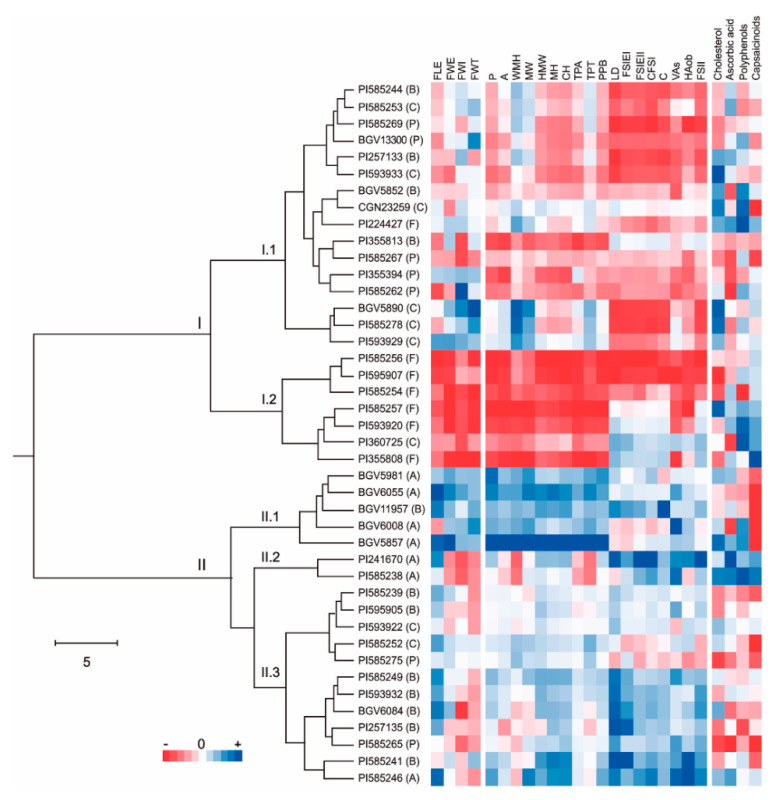
Cluster analysis (Ward coefficient) based on four biochemical traits, four conventional fruit descriptors, and eighteen TA attributes for the forty-two Ecuadorian accessions. The color code matrix represents the variables standardized to z-scores. Letters in brackets indicate the *Capsicum* species (A = *C. annuum*, B = *C. baccatum*, C = *C. chinense*, F = *C. frutescens*, P = *C. pubescens*).

Hierarchical clustering was performed to identify groups of promising accessions with the most interesting traits. The parameters employed for that purpose were selected according to their contribution to the variance on the PCA outcome. Hence, only four conventional traits and eighteen TA attributes with correlation coefficients higher than 50% were considered for cluster analysis. Four biochemical compounds (ascorbic acid, polyphenols, remaining cholesterol, and capsaicinoids) were used to accomplish the assembly of pepper accessions. All variables were standardized to z-scores. The clustering pattern was independent of *Capsicum* spp. and primarily based upon fruit morphology and nutraceutical content (Figure 5). Two main groups (I and II) could be differentiated. The first one (I) comprised accessions mainly from *C. chinense*, *C. frutescens*, and *C. pubescens* with fruits of small and medium sizes and shapes from round to triangular. The subdivision of this group established two subgroups, one of which (I.2) clearly corresponded to *C. frutescens* accessions. The majority of pepper fruits within that subgroup were pungent (high level of capsaicinoids), of small size, and variable shape. The second cluster (II) contained mostly *C. annuum* and *C. baccatum* accessions with huge fruits of triangular and elongated shapes. The partitioning of this group distinguished a combination of *C. annuum* (II.1) holding the fruits with the largest size and lowest amount of capsaicinoids. Conversely, group II.3 consisted of *C. baccatum* accessions with high values for fruit shape indices, but low amounts of bioactive compounds (Figure 5). Interestingly, the two *C. annuum* accessions PI241670 and PI585238, which stand out for containing values above average for all the assessed nutraceutical compounds, constituted a highly distinctive group (II.2). Pepper fruits with the greater capabilities for inhibiting cholesterol oxidation and considerable amounts of vitamin C or phenols were broadly distributed across groups, except for II.3 (Figure 5).

## 3. Discussion

Health-promoting compounds have been identified in the five *Capsicum* domesticated species, although *C. annuum* and *C. frutescens* are the most widely used for commercial purposes [41]. However, the search for novel pepper genotypes containing beneficial compounds for humans and industry still remains relevant. Local *Capsicum* varieties coming from the primary areas of domestication, such as northwestern South America, constitute a promising source of valuable metabolites, as they might retain part of the wealth stored in primitive peppers [9]. In the present work, different phytochemical compounds were quantified on a set of pepper landraces (*Capsicum* spp.) from Ecuador and their correlation to accurately defined fruit morphometric traits was established.

Capsaicinoids were quantified in 83.3% of accessions within the Ecuadorian collection and total amounts varied from 0.10 to 8.58 mg/g DW, which agrees with the concentrations found in other comparable panels [42,43]. As expected, Cap, DHCap, and NDHCap were the majority components, even though minor capsaicinoids were detected as well. *C. frutescens* accessions showed the highest levels and the line PI355808 was at the top, exhibiting a pungency corresponding to ca. 138,000 Scoville Heat Units (SHU) (extremely hot) when applying a conversion factor of 161 SHU per mg/100 g of Cap or DHCap [44]. Various works have claimed *C. chinense* as the spiciest *Capsicum*, with Habanero being the habitual representative [42,45]. However, our results confirmed those by Meckelmann et al. [43], who reported *C. frutescens* accessions as the ones having the highest capsaicinoids content in a set of native Peruvian chili peppers. Similar outcomes were claimed by Bogusz et al. [46] in Brazilian *Capsicum* peppers but also for other diverse collections [47,48]. Unexpectedly, *C. annuum* exhibited slightly higher levels of capsaicinoids than *C. chinense*. This was due to the presence of two accessions (PI241670 and PI585238) with an atypical amount of capsaicinoids and an unusual ratio of Cap/DHCap compared to the literature [49,50]. The morphology of those peppers (cayenne type) suggest that they might be varieties employed for powder production, which frequently possess more capsaicinoids than those destined for fresh consumption [38]. *C. baccatum* was the least pungent, with maximum values of 1.60 mg/g DW, equivalent to ca. 24,000 SHU, which concurs with previous works [43,46,51]. The amount of capsaicinoids in *C. pubescens* was also relatively low compared to the other four *Capsicum* groups, with concentrations varying from 0.38 to 2.11 mg/g DW. These values were within the range reported by Meckelmann et al. [52] on a panel of thirty-two *C. pubescens* (Rocoto) from Peru. In addition, *C. pubescens* accession in the Ecuadorian collection displayed a low Cap/DHCap ratio and high levels of minor capsaicinoids, particularly nordihydrocapsaicin. Such a distinctive profile was reported in earlier publications [52,53,54]. 

Total polyphenols showed a large variation across the different accessions but not among the five *Capsicum* spp., which exhibited short ranges from 21 to 26 mg FAE/g DW. This suggested that all domesticated species contained accessions with low, medium, and high levels of phenols. Similar results were reported by van Zonneveld et al. [9] on *Capsicum* peppers from Bolivia and Peru. Indeed, phenolic contents comparable to ours were quantified by those authors in Peruvian peppers belonging to the five *Capsicum* species [43]. In contrast, some reports supported great variability in the levels of polyphenols among different *Capsicum* species [46,50,51]. Such inconsistency might be attributed to the different geographical origin of plants and environmental growing conditions [38,55]. The highest values of total phenolic compounds were found in *C. annuum*, particularly on accession PI585238, which also possessed atypical amounts of capsaicinoids. Considering that both types of compounds are principal contributors to the antioxidant activity of pepper fruits [56,57], this Ecuadorian accession emerged as a promising source of free radical scavenging molecules, able to protect human cells against oxidative damage. 

Ascorbic acid was evaluated in fresh peppers to avoid degradation due to drying processes [58]. The ascorbic acid values in peppers found in the literature vary considerably [33]. All Ecuadorian accessions assessed in the present work displayed levels of vitamin C greater than 100 mg/100 g, in contrast to works by Wahyuni et al. [28], who found low amounts of this vitamin in a large fraction of the *Capsicum* pepper accessions under evaluation. In our research, the largest differences in the vitamin C content were observed between *C. annuum* and *C. pubescens*, which displayed the highest and lowest values, respectively. Such an outcome is in line with that obtained by the majority of publications, ascribing the maximum amounts of vitamin C to *C. annuum* accessions, while describing *C. pubescens* as the *Capsicum* group with the lowest content of ascorbic acid [31,47,59]. Recently, Guevara et al. [39] and Pérez-Balladares et al. [40] demonstrated that chili peppers from Ecuador contained much higher amounts of Vitamin C than other legumes, tubers, and tropical fruits commonly consumed in the Andean country. In our work, the two *C. annuum* accessions with the highest concentrations of ascorbic acid were PI241670 and PI585238, which also showed high levels of capsaicinoids and phenolic compounds. Such compositional profile points at those pepper fruits as relevant depositories of antioxidant agents. The other *Capsicum* spp. accessions within the Ecuadorian collection, although they did not reach the levels of vitamin C recorded for *C. annuum*, would be more than satisfactory for the human recommended daily intake of this vitamin [60].

Knowledge on the nutraceutical composition of peppers is of limited value if it is not accompanied by an extensive and detailed characterization of fruit phenomics. Fruit size, shape, and weight are essential attributes for the establishment of market types and for the acceptance of new pepper varieties by farmers and consumers [61]. In the present work, a total of 86 plant and fruit characters where investigated by testing traditional descriptors commonly used by breeders and a high-throughput digital assessment, which allowed highly precise dissection of pepper fruit morphometry. The Ecuadorian collection displayed broad diversity for plant and fruit traits. Significant differences were found across species but also among accessions within the same *Capsicum* group. Those conventional traits linked to the magnitude of plant and fruits, such as PHE, PWI, SLE, FWE, FLE, and FWI, mostly contributed to the variability, as previously reported [62,63]. Regarding the TA analysis, descriptors under the categories of basic measurements, shape index, and latitudinal section, exhibited the highest contribution to the variance. Similar findings were reported not only for pepper [23,24] but also for tomato [20,27] and eggplant [21]. ANOVA analysis revealed that *C. annuum* statistically diverged from the other species, exhibiting the highest values for traits related to size, while *C. frutescens* showed the lowest measurements for identical parameters. This is in line with results described by Tripodi and Greco [24] on a large collection of cultivated and wild peppers.

Several studies performing high-throughput phenotyping in different pepper collections have been recently published [23,24,25,26]. However, to the best of our knowledge, no works exist attempting to evidence the impact of morphology on fruit composition for health-promoting compounds. To investigate the correlation between these two groups of attributes emerged as a mandatory task, since this relationship might affect the speed at which beneficial metabolites for human-health could be introgressed into commercial pepper cultivars. Phenols and vitamin C were positively correlated to capsaicinoids at coefficients higher than 0.4. This support the work by Kantar et al. [31], who found that the levels of vitamin C concurrently increased with the amount of capsaicin. Likewise, high and positive correlations between the phenolic composition and capsaicinoids levels were reported for pepper fruits [50]. All biochemical traits, except for cholesterol, negatively interacted with quantitative conventional parameters describing fruit morphology, with the highest coefficients found for the correlation between capsaicin/capsaicinoids and fruit wall thickness. In the same manner, all nutraceutical compounds apart from vitamin C and cholesterol showed a negative association with the majority of TA descriptors under size and latitudinal section but also with rectangular and eccentricity traits. Such results suggest that pepper fruits with larger sizes, more rectangular shapes, and medium ratios for height/width, i.e., bell pepper types, contain lower amounts of polyphenols and capsaicinoids, but higher contents of ascorbic acid and a greater capability for preventing cholesterol oxidation after heating.

Correlations between conventional and TA descriptors were checked as well. FWE and FLE significantly and positively correlated with every TA attribute related to fruit size but also with TPA, TPT, and PPB. Associations among fruit weight, shape, and size were further highlighted in tomato by Nankar et al. [27]. Conversely, FWI and FWT displayed a significantly positive interaction only with WMW, MW, and TPT. Furthermore, manually measured fruit width was slightly lower than the maximum width derived from TA. Identical observations were reported by other authors [21,26], who suggested that this inconsistency might be associated to the way both parameters are quantified. Hence, width measured by TA was recorded where longitudinal sections are wider, so it could hardly vary depending on fruit shape. The network of correlations for TA evidenced positive and significantly tight connections between size-based traits and those related to pericarp magnitude (TPT, TPA, and PPB), indicating that the fruit wall was thicker in larger fruits. This is additionally supported by negative associations between pericarp thickness and fruit shape indices (FSIEI, FSIEII, CFSI, FSII). Maximum height was positive correlated to fruit shape indices but not to MW, in agreement with evidence obtained by Naegele et al. [23]. The TA shape designations (Circular, Rectangular, and Ellipsoid) were significantly associated with Asymmetry (VAs, HAob), shape indices (FSIEI, FSIEII, CFSI), and internal eccentricity (EC, PEC, DEC, FSII, ECAI). Even though, Ellipsoid and Circular correlated positively, while Rectangular did it in a negative manner, as previously reported by Naegele et al. [23], and Tripodi and Greco [24].

Variations in biochemical composition and agro-morphological properties can be explained not only by species differences, but also by the growing conditions of crops. The soil, water supply, and amount of sunlight and precipitation, as well as the changes in temperature, will cause alterations in the concentrations of phytochemical compounds and in the morphology of plants and fruits, although this will be more evident in the former [37,38]. For that reason, the variability on biochemical profiles, along with plant and fruit morphologies were investigated for *C. annuum*, *C. chinense,* and *C. baccatum* in the diverse geographical areas existing in Ecuador (Coast, Andes, Austro, and Amazon). Retrieving *Capsicum* spp. accessions covering the whole Ecuadorian territory became challenging, due to the highly dissimilar natural regions available in this country, and the fact that some species are restricted to some particular locations, e.g., *C. pubescens* requires a cool, freeze-free environment with long growing seasons [64]. *C. annuum* accessions from the Coastal and Austro regions possessed higher amounts of vitamin C and capsaicinoids than those from the Andes. Interestingly, Coastal pepper fruits had the lowest weight, area, perimeter, pericarp area, and thickness but the highest values for fruit shape indices, which measured the ratio of height to width. This suggests that *C. annuum* types similar to Cayenne are predominant in this region. *C. baccatum* accessions with significantly higher content of polyphenols and capsaicinoids were found at the Austro. In this case, an obviously distinctive morphology for those fruits could not be established in comparison to the Coast and Andes, although plants with larger dimensions were observed. *C. chinense* grown in the Amazon produced more polyphenols and capsaicinoids but less vitamin C than those from the Coast. Such biochemical expressions appeared in larger fruits with higher height, width, perimeter, and area. Differences between locations were also reported by Meckelmmann et al. [55] and van Zonneveld et al., [9] who evaluated a collection of Peruvian and Bolivian chili peppers (*Capsicum* spp.) at different climate zones in Peru. At a larger geographical scale, Naegele et al. [23] showed that peppers from South America had a smaller perimeter, area, and pericarp thickness than those from the European continent. Despite the well-defined Ecuadorian geographical regions with highly distinctive environmental conditions, and the low number of accessions per area prevented us from drawing more clear conclusions on the effect of growing locations. A higher number of lines should be considered to determine whether some regions are especially suited for obtaining peppers with certain beneficial characteristics or which accessions might consistently grow and produce phytochemicals at any climate zone.

Principal component analysis (PCA) was carried out to determine the most effective attributes in discriminating among accessions. The first three components explained only 53.6% of the total variance, likely due to the excessive number of variables analyzed. Pereira-Dias et al., [25] working on a collection of Spanish peppers, recorded higher percentages of variation when PCA analysis was performed separately for conventional and digital phenotyping, than that obtained when both sets were combined. The most important positively correlated traits for the first PC were conventional descriptors (FWE, FLE, FWI, and FWT) and TA attributes from basic measurements and latitudinal section, although the former contributed more than the latter, accordingly with previous works [20,21,25,26]. In addition, this component was negatively correlated with polyphenols and capsaicinoids, i.e., larger fruits possessed a low content of these compounds. Such results confirmed those obtained by the correlation analysis. The second component was primarily associated with TA shape indices, LD, Circular, VAs, and HAob, while PC3 was basically explained by nutraceutical parameters. This result suggested that those traits mostly contributing to the variance explained by the three axes were the ones subjected to the higher selection pressure imposed by humans. Our data partly agreed with other studies dealing with pepper phenomics, with differences likely arising from differences across the groups of accessions investigated. Nevertheless, those descriptors related to fruit size and shape always explained a high level of diversity, regardless of the collection considered [24,25,26,62]. The projection of the accessions on a two-dimensional plot confirmed that each *Capsicum* species was diverse for morphological and biochemical traits, as accessions of a single group presented a certain degree of dispersion, plotting at different areas of the graph. This was particularly evident for *C. annuum*, which displayed variable accessions from those with large fruits, thick walls, moderate height/width ratios, and triangular shapes, to those with tiny, thin-walled fruits, with high height/width ratios, and extremely elongated shapes (Cayenne type). The fruits from *C. chinense* accessions exhibited more variation in size than in shape, with fruit weight ranging from 2.5 to 27 g, and shapes varying from rectangular forms (Habanero type) to more triangular profiles. In contrast, *C. baccatum* fruits possessed less variable sizes and pericarp thickness but shapes that differed from the “Peruvian ají type”; a long, medium size pepper with high shape indices, to almost round peppers with very low fruit shape indices. *C. frutescens* accessions constituted the most homogenous group, except for PI224427. That accession was misclassified in a previous work that genetically placed it as more closely related to *C. chinense* [11]. Current data, based on fruit morphology and biochemical composition, confirmed that accession PI224427 should be ascribed to the *C. chinense* group. The other *C. frutescens* showed very small fruits highly similar to Tabasco type peppers. The fact that most of the variation in fruit features occurred in *C. annuum* was somehow expected. This *Capsicum* species represented the most commonly cultivated and consumed around the world and the one that has suffered from more intensive human selection pressures, which greatly modified the morphometry of fruits from that of the wild ancestor *C. annuum* var. *glabriusculum* [65,66].

Hierarchical clustering allowed the organization of accessions into groups according to their common characteristics. The outcome of that analysis reflected similar results as those obtained in PCA, where Ecuadorian pepper accessions arranged independently of their species and according to the fruit morphology and content in biochemical components. A group of *C. frutescens* accessions with high amounts of capsaicinoids, polyphenols, and ascorbic acid were identified. Likewise, cluster analysis revealed the existence of *C. annuum* with huge, fleshly fruits and very low pungency. A group of *C. baccatum* accessions with highly diverse fruit morphology and moderate pungency were detected as well. Presumably, the most promising accessions were *C. annuum* PI241670 and PI585238, which exhibited outstanding levels for all health-promoting compounds analyzed in this work. These accessions could be directly integrated into breeding programs, avoiding problems of crossability associated with other *Capsicum* species [4].

Fruit shapes and sizes derived from TA analysis have been investigated for their role in disease resistance by Naegele et al. [23]. These authors reported that pepper fruit perimeter, width, and pericarp thickness had a slight positive correlation with Phytophthora fruit rot, whereas fruit shape index had a slightly negative one. Our own previous work revealed that the majority of Ecuadorian accessions harbored potential resistance to some or several common diseases of pepper [67]. Among the goals of the present study was not the identification of associations between fruit traits and disease response. Nevertheless, the combination of both datasets will be extremely useful to select valuable accessions, not only for their morpho-chemical attributes, but also for their ability to confront pathogen attack.

The present work demonstrated that Ecuador, one of the postulated places for the origin of the genus *Capsicum*, possesses a substantial diversity of pepper fruits with different sizes and shapes, carrying variable amounts of beneficial compounds for human health and nutrition. The existence of interesting traits in one unique accession would guarantee higher speed in their transference to commercial pepper cultivars. To do so, it is fundamental to understand the genetic basis underlying such promising attributes. In peppers, conventional and TA descriptors have been already utilized to identify single nucleotide polymorphisms (SNPs) related to fruit size and shape [68,69]. Our collection of Ecuadorian landraces, together with a large set of pepper (*Capsicum* spp.) local varieties from Peru, Bolivia, Mexico, Iberian Peninsula (secondary center of pepper diversification), and Mediterranean areas were subjected to genotyping-by-sequencing technology (unpublished data). Fruit phenomics, biochemical, and genotypic data are now being integrated through a genome wide association study (GWAS) with the aim of identifying those genomic regions governing the most relevant fruit traits in pepper.

## 4. Materials and Methods

### 4.1. Plant Material

**Figure 6:**
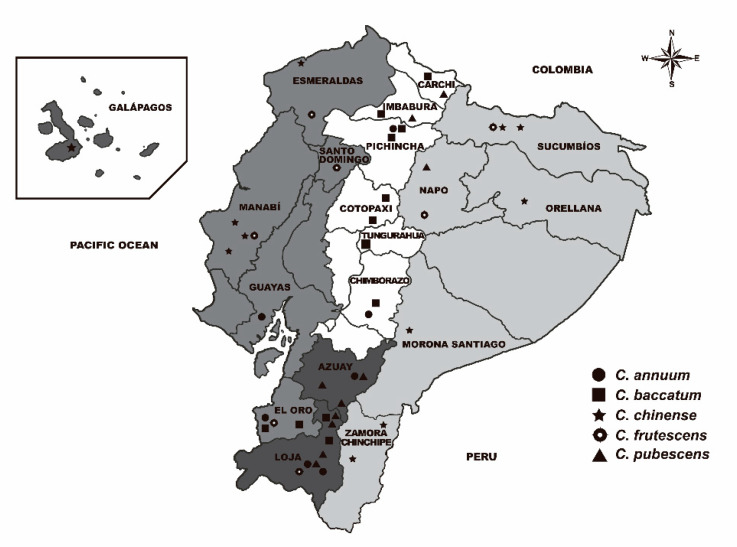
Geographical distribution of Ecuadorian pepper accessions. Provinces colored in white represent the Andes, those in grey the Coast, light grey the Amazon and dark grey the Austro.

Forty-eight accessions from Ecuador depicting five domesticated species including *C. annuum* (7), *C. chinense* (11), *C. frutescens* (8), *C. pubescens* (10), and *C. baccatum* (12), were selected for this work (Table S6). They were kindly provided by the U.S. Department of Agriculture (USDA)—Agricultural Research Service (ARS)—Plant Genetic Resources Conservation Unit (Griffin, GA, USA), the Instituto de Conservación y Mejora de la Agrodiversidad Valenciana (COMAV, Valencia, Spain), and the Center for Genetic Resources (CGN, Wageningen, The Netherlands) (Table S6). When possible, they were selected to cover the four Ecuadorian geographical regions: Coast, Andes, Austro, and Amazon (Figure 6). For the sake of clarity, the accession PI585278 from the Galapagos Islands was considered within the Coastal region. This collection was previously characterized for genetic diversity and molecular markers linked to disease resistance and pungency-related traits [11,67]. The forty-eight landraces were cultivated during 2017 in an experimental field at the Technical University of Machala (Machala, El Oro, Ecuador) (03°16′ S, 79°54′ W). Ten plants per accession were grown following a completely randomized design. Cultivation was carried out according to the horticultural practices in the area for local pepper varieties. Thus, plants were drip irrigated and fertilized with a mix of nitrogen, phosphorus, and potassium before and after transplanting. Phytosanitary treatments against whiteflies, aphids, and spider mites were applied according to their population levels.

### 4.2. Conventional Descriptors Assessment

All accessions were characterized by using 45 conventional morphological descriptors for *Capsicum* following the protocol described by the International Plant Genetic Resources Institute (IPGRI) [70]. These morphological descriptors comprised different traits of the whole plant (18), inflorescence (12), and fruit (15). Descriptors and abbreviations are provided in Table S7. Fifteen descriptors were quantitative, while thirty were qualitative or pseudo-qualitative and they were measured according to pre-established scales [70] (Table S7). Ten fruits per plant were harvested at maturity stage and employed for conventional characterization in the laboratory.

### 4.3. Fruit Characterization with Tomato Analyzer

Between 22 and 30 fruits per accession, were harvested at maturity and subjected to digital phenotyping. Pepper fruits were longitudinally and transversally cut and scanned with an Epson L365 photo scanner (Epson, Amsterdam, The Netherlands) at a resolution of 300 dpi. Stored images (TIF format) were subsequently analyzed using Tomato Analyzer version 4 software [16,17]. A total of 41 fruit parameters, categorized into basic measurements (7), fruit shape index (3), blockiness (3), homogeneity (3), proximal fruit end shape (4), distal fruit end shape (4), asymmetry (6), internal eccentricity (5), and latitudinal section (6), were automatically recorded (Table S7). A complete description of these traits can be found elsewhere [16,17,18]. Default settings were used for all categories, although points were adjusted manually when the software was unable to accurately identify the outline of a trait. Individual measurements of each fruit were used to obtain an average value for the corresponding accession.

### 4.4. Analytical Methods

Ripe fruits were collected from different plants of the same accession and divided into two bulks: (1) Ten fresh fruits were washed, pooled, and cut into pieces. They were homogenized with a mortar and pestle and filtered through gauze. The resulting pepper juice was directly used for ascorbic acid determination. (2) Ten fresh fruits were pooled and oven-dried at 55 °C to a constant weight for approximately 72 h. Then, they were ground in an electric grinder and the obtained powder was stored into plastic tubes at −20 °C until further analysis. This sample was used for the assessment of polyphenols, capsaicinoids, and the inhibition of cholesterol oxidation after heating. All analytical measures were performed in triplicate.

#### 4.4.1. Analysis of Ascorbic Acid

The quantification of ascorbic acid was done by linear voltammetry on a potentiostat (Princeton Applied Research). Five mL of pepper juice was mixed with different aliquots (5, 12, 17, 25, and 35 mL) of ascorbic acid (5 mg/mL) and supplemented with up to 50 mL NaNO_3_ electrolyte. Then, the mixtures were placed in an electrochemical cell consisting of three electrodes: one vitreous carbon electrode as a working electrode, one Ag/AgCl (sat. KCl) as a reference electrode, and one platinum as the auxiliary electrode. A potential between 0 and 1.5 mV and a sweep speed of 0.2 mV/s were applied. Ascorbic acid was quantified by a linear regression model obtained from the potential oxidation peaks observed on the voltammograms.

#### 4.4.2. Determination of Total Polyphenols Content

Twenty-five milligrams of pepper powder was mixed with 5 mL of methanol and incubated for 1 h at 60 °C with occasional shaking. Then, the crude extract was filtered through a 0.45 µm syringe filter (Millipore) and stored at 4 °C for further use. The Folin–Ciocalteu method was applied according to Singleton and Rossi [71]. Fifty μL of extract was diluted with 750 μL of distilled water and mixed with 50 μL of Folin–Ciocalteu reagent. After an incubation time of 3 min, 150μL of 20% Na_2_CO_3_ was added. The mixture was shaken vigorously and incubated for 2 h at room temperature in the dark. Absorbance against a blank was measured at 760 nm using a Thermo Heλios spectrophotometer. The standard curve was constructed with ferulic acid. The results were expressed as ferulic acid equivalents (FAE) in mg/g dry weight (DW).

#### 4.4.3. Determination of the Inhibition Capability in Preventing Cholesterol Oxidation during Heating

The same pepper extracts employed for polyphenol determination were used for this analysis. The capability of pepper fruits to prevent the oxidation of cholesterol during heating was determined according to Sun et al. [36]. Briefly, 100 µL of cholesterol solution (1 mg/mL in hexane) and 50 µL of pepper extract were mixed in a test tube and subsequently, the solvents were evaporated at 50 °C. The control was prepared by adding only the cholesterol solution in the test tube. The dried mixture was heated at 175 °C for 20 min. Then, the tube was cooled down, 1 mL of methanol was added, and the mixture was vortexed for 30 s. The solution was transferred to HPLC vials to determine the remaining cholesterol. A HPLC system Water Alliance including a 2695 Separation Module, a 2996 PDA detector, and a Spherisorb ODS2 C18 reversed phase column (4 μm, 150 mm × 4.6 mm) was employed. The mobile phase consisted of methanol:acetone (90:10) at a flow rate of 0.8 mL/min. The wavelength for quantifying the cholesterol was 215 nm. The percentage of the remaining cholesterol was obtained by comparing the remaining concentrations to the original concentration.

#### 4.4.4. Extraction and Analysis of Capsaicinoids

Pepper extracts were prepared by adding 100 mg of powder into 1 mL of acetonitrile. The mixture was incubated for 4 h at 80 °C and vortexed every 30 min. After cooling to room temperature, the sample was centrifuged at 1000 rpm for 5 min. The supernatants were then filtered through a 0.45 µm syringe filter (Millipore).

The identification and quantification of capsaicinoids was performed on an HPLC Water Alliance system containing a 2695 Separation Module, 2996 PDA detector, and equipped with a Spherisorb ODS2 C18 reversed phase column (4 μm, 150 mm × 4.6 mm), which was maintained at 30 °C. The detector was set to 280 nm for excitation and to 320 nm for detection. Separation of different capsaicinoids was achieved by isocratic elution with acetonitrile and double distilled water (55:45, v/v) at a flow rate of 1 mL/min. Cap and DHCap were quantified from the calibration curves obtained from standard solutions of capsaicin (97%) and dihydrocapsaicin (90%) (Sigma-Aldrich, Steinheim, Germany). Since there are no commercial standards of NDHCap, HCap, and HDHCap, these were quantified from the calibration curve of DHCap (for NDHCap and for HDHCap) and from the calibration curve of Cap (for HCap), given the structural similarities between these molecules [72]. Capsaicinoids content was calculated as the sum of Cap, DHCap, HCap, NDHCap, and HDHCap.

### 4.5. Data Analyses

Analysis of variance (ANOVA) was performed for conventional descriptors, digital parameters, and biochemical compounds. Means, standard deviations, and coefficient of variation (expressed in percentage as the ratio between standard deviation and mean) were used for descriptive analysis of traits. Significant differences among means were detected using Tukey HSD test (*p* < 0.05). Statistical analyses were performed with SPSS version 17.0 software [73]. The correlations among all evaluated parameters were estimated by using the Pearson’s test at *p* < 0.05 after Bonferroni’s adjustment for multiple comparisons [74]. The calculation of coefficients and visualization of correlograms was performed with Rcmdr and Corrplot packages implemented in R 3.6.0 software [75]. Conventional (quantitative), TA, and biochemical attributes were comprehensively examined through principal component analysis (PCA). In addition, the similarity across accessions was estimated by agglomerative hierarchical cluster analysis (HCA) using Ward’s coefficient. Multivariate analyses were conducted with the computer software SPSS version 17.0 [73] and R 3.6.0. [75]. Graphical representation of the tree was performed with MEGA X software [76].

## Figures and Tables

**Table 1:** Analysis of variance for biochemical traits among the five *Capsicum* species. Cap = Capsaicin, DHCap = Dihydrocapsaicin, NDHCap = Nordihydrocapsaicin, HCap = Homocapsaicin, HDHCap = Homodihydrocapsaicin.

	Ascorbic Acid	Polyphenols	Cholesterol	Cap	DHCap	NDHCap	HCap	HDHCap	Capsaicinoids
Sum of squares	236373.1	348.2	8144.4	87.2	15.4	0.7	0.024	0.016	159.8
Mean squares	59093.3	87.0	2036.1	21.8	3.9	0.2	0.006	0.004	39.9
F value ^†^	16.4 ***	3.7 **	5.0 **	22.8 ***	9.9 ***	7.1 ***	4.9 ^ns^	4.0 ^ns^	15.2 ***

^†^, ** and *** Significant at *p* < 0.01 and 0.001, respectively; ^ns^ not significant.

**Table 2:** Analysis of variance for quantitative conventional descriptors among the five *Capsicum* species (left). Mean (Coefficient Variation %) for each group (right). Different letters within the same row indicate significant differences at *p* < 0.05. PHE = Plant height, PWI = Plant width, SLE = Stem length, SDI = Stem width, MLL = Mature leaf length, MLW = Mature leaf width, CLE = Corolla length, ALE = Anther length, FILE = Filament length, FLE = Fruit length, FWI = Fruit width, FWE = Fruit weight, FPL = Fruit pedicel length, FWT = Fruit wall thickness, NL = Number of locules.

Trait	Sum of Squares	F Value ^†^	*C. annuum*			*C. baccatum*			*C. chinense*			*C. frutescens*			*C. pubescens*		
**PHE**	53049.3	44.93 ***	72.06	(17.6)	b	65.71	(33.0)	b	67.55	(25.1)	b	87.39	(18.5)	a	48.69	(26.7)	c
**PWI**	28384.1	33.85 ***	67.59	(7.0)	c	87.66	(18.0)	a	78.33	(16.9)	b	65.84	(17.7)	c	77.95	(27.1)	b
**STE**	27092.0	38.48 ***	17.95	(49.1)	b	12.84	(82.7)	b,c	12.94	(96.9)	b,c	33.11	(73.5)	a	7.41	(41.2)	c
**SDI**	730.9	43.09 ***	5.58	(41.0)	b	7.35	(29.9)	a	7.31	(29.1)	a	6.29	(38.2)	b	3.71	(17.0)	c
**MLL**	1563.4	62.05 ***	12.68	(26.2)	c	11.24	(20.7)	d	14.50	(11.3)	b	16.69	(16.7)	a	11.92	(21.2)	c,d
**MLW**	258.3	24.30 ***	6.224	(24.8)	b	6.749	(31.8)	b	7.744	(17.1)	a	8.401	(14.3)	a	6.351	(21.7)	b
**CLE**	13.706	22.32 ***	1.744	(24.7)	a	1.412	(28.1)	b,c	1.519	(30.1)	b	1.254	(12.9)	c	1.760	(23.0)	a
**ALE**	4.571	1.45 ^ns^	3.086	(27.4)	-	3.308	(24.7)	-	3.167	(28.5)	-	3.090	(30.7)	-	3.029	(31.3)	-
**FILE**	33.5	9.78 ***	4.100	(20.0)	a	4.075	(20.4)	a	3.811	(25.8)	a	3.284	(33.9)	b	3.700	(24.5)	a
**FLE**	33.5	9.78 ***	8.319	(36.0)	a	6.659	(39.1)	b	5.162	(38.7)	c	2.524	(49.9)	d	4.231	(35.7)	c
**FWI**	82.7	21.33 ***	2.283	(48.4)	a	1.699	(38.5)	b	2.431	(39.2)	a	1.151	(61.4)	c	2.183	(67.7)	a
**FWE**	9420.0	34.38 ***	19.05	(76.6)	a	10.95	(53.1)	b	10.12	(80.8)	b	2.83	(95.2)	c	8.34	(56.6)	b
**FPL**	53.9	16.41 ***	3.693	(23.3)	a	3.738	(26.9)	a	3.346	(26.8)	a,b	2.781	(39.3)	c	3.051	(16.3)	c,b
**FWT**	185.8	39.39 ***	3.357	(38.6)	a	2.825	(31.0)	b	3.656	(36.3)	a	1.612	(51.7)	c	3.286	(32.0)	a,b
**NL**	27.8	7.96 ***	2.800	(33.1)	a	2.942	(27.3)	a	2.811	(37.3)	a	2.552	(41.1)	a,b	2.200	(39.9)	b

^†^, *** Significant at *p* < 0.001; ^ns^ not significant.

**Table 3:** Analysis of variance for TA descriptors among the five *Capsicum* species (left). Mean (Coefficient Variation %) for each group (right). Different letters within the same row indicate significant differences at *p* < 0.05. P = Perimeter, A = Area, WMH = Width mid-height, MW = Maximum width, HMW = Height mid-width, MH = Maximum height, CH = Curved height, FSIEI = Fruit shape index external I, FSIEII = Fruit shape index external II, CFSI = Curved fruit shape index, PFB = Proximal fruit blockiness, DFB = Distal fruit blockiness, FST = Fruit shape triangle, E = Ellipsoid, C = Circular, R = Rectangular, SH = Shoulder height, PAMI = Proximal angle micro, PAMA = Proximal angle macro, PIA = Proximal indentation area, DAMI = Distal angle micro, DAMA = Distal angle macro, DIA = Distal indentation area, DEP = Distal end protrusion, Ob = Obovoid, Ov = Ovoid, VAs = V.Asymmetry, HAob = H.Asymmetry.Ob, HAov = H.Asymmetry.Ov, WWP = Width widest position, EC = Eccentricity, PEC = Proximal eccentricity, DEC = Distal eccentricity, FSII = Fruit shape index internal, ECAI = Eccentricity area index, LD = Lobedness degree, TPA = Tomato pericarp area, TPAR = Tomato pericarp area ratio, TPT = Tomato pericarp thickness, TPTR = Tomato oericarp tickness ratio, PPB = Pepper pericarp boundary.

Trait	Sum of Squares	F Value ^†^	*C. annuum*			*C. baccatum*			*C. chinense*			*C. frutescens*			*C. pubescens*		
**P**	4076327.52	510.28 ***	258.41	(23.6)	a	163.21	(32.9)	b	140.64	(22.8)	c	63.62	(47.7)	e	111.47	(29.4)	d
**A**	395699848.67	209.54 ***	2154.81	(66.2)	a	1036.27	(51.2)	b	1024.48	(40.3)	b	241.20	(117.4)	d	609.20	(50.6)	c
**WMH**	22387.62	88.74 ***	23.28	(49.6)	b	19.44	(23.9)	c	25.51	(38.2)	a	12.23	(65.7)	d	20.01	(23.6)	c
**MW**	62417.36	297.27 ***	37.13	(24.6)	a	24.23	(24.4)	c	28.86	(28.4)	b	12.90	(60.9)	d	22.93	(20.4)	c
**HMW**	407730.65	289.78 ***	80.42	(28.4)	a	60.23	(40.4)	b	46.68	(27.8)	c	21.56	(51.5)	e	37.24	(40.5)	d
**MH**	556367.80	310.73 ***	89.13	(29.9)	a	69.28	(40.5)	b	49.93	(27.0)	c	22.52	(50.9)	e	39.30	(40.2)	d
**CH**	631209.90	353.83 ***	97.74	(25.0)	a	70.93	(40.1)	b	52.95	(23.6)	c	24.44	(46.8)	e	42.97	(43.6)	d
**FSIEI**	394.52	109.51 ***	2.956	(23.3)	b	3.203	(38.6)	a	1.989	(43.4)	c	1.960	(43.6)	c	1.957	(37.0)	c
**FSIEII**	1433.10	164.84 ***	4.927	(47.7)	a	3.715	(42.0)	b	2.135	(49.3)	c	2.115	(54.6)	c	1.943	(41.8)	c
**CFSI**	2108.96	186.94 ***	5.977	(45.6)	a	4.206	(42.5)	b	2.387	(46.3)	c	2.502	(48.7)	c	2.314	(45.4)	c
**PFB**	14.72	157.66 ***	0.841	(25.3)	a	0.531	(21.4)	d	0.544	(29.7)	c,d	0.581	(20.6)	c	0.693	(23.9)	b
**DFB**	12.80	85.90 ***	1.086	(14.8)	a	0.921	(14.0)	b	0.842	(28.3)	c	0.870	(33.0)	c	0.729	(13.5)	d
**FST**	37.61	43.63 ***	0.950	(47.9)	a	0.582	(25.2)	b	0.901	(83.1)	a	1.020	(46.3)	a	0.995	(30.0)	a
**E**	1.07	320.09 ***	0.162	(32.6)	a	0.087	(21.6)	b	0.090	(22.9)	b	0.072	(24.6)	c	0.073	(42.0)	c
**C**	4.40	89.19 ***	0.344	(21.0)	a	0.337	(41.2)	a	0.223	(46.9)	b	0.227	(50.9)	b	0.203	(40.5)	b
**R**	1.33	80.71 ***	0.375	(22.7)	d	0.448	(12.2)	b	0.428	(14.3)	c	0.430	(15.9)	c	0.491	(10.9)	a
**SH**	2.63	35.15 ***	0.498	(6.7)	a	0.455	(19.9)	b	0.411	(40.1)	c	0.347	(57.9)	d	0.413	(34.9)	c
**PAMI**	646218.10	39.37 ***	162.27	(57.7)	a	94.07	(81.7)	d	119.68	(32.1)	c	117.54	(46.4)	c	140.42	(19.3)	b
**PAMA**	914210.13	54.28 ***	137.25	(90.8)	a	70.31	(55.0)	c	105.88	(41.9)	b	96.08	(49.2)	b	144.54	(39.6)	a
**PIA**	1507.27	306.59 ***	3.763	(67.8)	a	0.682	(58.8)	b	0.692	(58.7)	b	0.564	(120.2)	b	0.802	(61.2)	b
**DAMI**	188471.47	20.39 ***	118.99	(28.8)	c	114.58	(20.9)	c	147.03	(38.8)	a	135.58	(60.3)	a,b	131.92	(17.8)	b
**DAMA**	60989.22	7.09 ***	121.42	(65.8)	c	106.02	(36.2)	a	121.67	(29.4)	c	112.90	(39.1)	b,c	123.47	(18.9)	c
**DIA**	2.34	46.69 ***	0.038	(58.6)	b	0.021	(72.0)	b	0.048	(119.3)	b	0.026	(79.3)	b	0.153	(182.1)	a
**DEP**	10.19	34.65 ***	0.417	(95.4)	a	0.245	(109.7)	b	0.225	(89.5)	b	0.274	(116.2)	b	0.088	(58.9)	c
**Ob**	5.18	81.82 ***	0.402	(24.1)	a	0.356	(36.7)	b	0.303	(50.1)	c	0.246	(54.6)	d	0.204	(40.8)	e
**Ov**	4.10	47.15 ***	0.169	(153.3)	a	0.000	(486.2)	d	0.078	(185.3)	c	0.080	(155.7)	c	0.126	(127.9)	b
**VAs**	31.52	340.03 ***	0.587	(39.3)	a	0.263	(69.9)	b	0.190	(45.2)	c	0.062	(56.1)	e	0.144	(95.4)	d
**HAob**	128.97	144.24 ***	1.128	(63.4)	a	0.746	(83.7)	b	0.489	(50.7)	c	0.152	(92.7)	d	0.217	(114.0)	d
**HAov**	29.84	61.81 ***	0.472	(168.1)	a	0.000	(718.5)	d	0.090	(165.3)	b,c	0.045	(145.6)	c,d	0.148	(217.8)	b
**WWP**	6.55	111.26 ***	0.766	(13.2)	a	0.680	(16.1)	b	0.605	(23.9)	c	0.562	(22.7)	d	0.549	(21.4)	d
**EC**	0.61	71.12 ***	0.688	(9.9)	c	0.743	(6.5)	b	0.744	(5.2)	b	0.759	(4.8)	a	0.752	(4.4)	a,b
**PEC**	2.18	79.77 ***	1.018	(19.0)	a	0.911	(3.8)	b	0.893	(3.4)	b	0.905	(3.5)	b	0.896	(3.6)	b
**DEC**	0.42	84.92 ***	0.929	(5.6)	a	0.893	(2.3)	b	0.880	(3.6)	c	0.868	(5.0)	d	0.896	(3.4)	b
**FSII**	976.05	118.11 ***	4.159	(49.0)	a	3.780	(41.0)	b	2.097	(52.4)	c	2.205	(54.6)	c	2.021	(53.3)	c
**ECAI**	6.75	156.65 ***	0.647	(34.7)	a	0.467	(13.8)	b	0.437	(11.9)	c,d	0.418	(13.4)	d	0.447	(11.9)	b,c
**LD**	101373.30	106.07 ***	35.75	(38.7)	b	39.95	(49.2)	a	21.36	(66.3)	c	21.26	(62.5)	c	17.62	(62.5)	c
**TPA**	86661426.60	204.01 ***	1024.65	(69.0)	a	471.15	(45.5)	b	430.75	(38.9)	b,c	110.90	(119.3)	d	360.73	(46.6)	c
**TPAR**	2.51	27.54 ***	0.441	(1.1)	b	0.446	(3.5)	b	0.556	(57.4)	a	0.453	(1.1)	b	0.444	(2.0)	b
**TPT**	621.97	163.19 ***	3.850	(43.9)	a	3.101	(22.1)	c	3.523	(23.9)	b	1.580	(52.1)	d	2.964	(25.5)	c
**TPTR**	0.26	27.72 ***	0.209	(12.0)	b,c	0.213	(11.6)	b	0.241	(39.1)	a	0.202	(12.5)	b,c	0.200	(5.0)	c
**PPB**	2804013.45	421.79 ***	211.31	(30.9)	a	130.74	(35.3)	b	111.59	(24.6)	c	48.97	(49.5)	e	91.01	(25.9)	d

^†^, *** Significant at *p* < 0.001.

## Supplementary Materials

The following are available online at https://www.mdpi.com/2223-7747/9/8/986/s1, Table S1: Analysis of variance for biochemical traits among 42 pepper landraces (above). Mean ± SD for each accession (below). Last five columns represent the relative percentage for each capsaicinoids. Last row indicates the average value (Coefficient Variation) for each trait. Nd stands for not detected, Table S2: Analysis of variance for quantitative conventional descriptors among 42 pepper landraces (above). Mean ± SD for each accession (below). Last row indicates the average value (Coefficient Variation) for each descriptor, Table S3: Analysis of variance for qualitative conventional descriptors among 42 pepper landraces. See Table S7 for acronyms, Table S4: Analysis of variance for TA descriptors among 42 pepper landraces (above). Mean ± SD for each accession (below). Last row indicates the average value (Coefficient Variation) for each descriptor, Table S5: Mean and Coefficient of Variation (%) for conventional and digital descriptors in *C. annuum*, *C. chinense*, and *C. baccatum* within each geographical region. Different letters within the same column indicate significant differences at *p* < 0.05. Only the parameters showing significant differences among regions are represented, Table S6: List of accessions employed in this work. Na stands for not available, Table S7: List of conventional descriptors [70] and digital traits measured with Tomato Analyzer software [16,17], Figure S1: Chemical structure of ascorbic acid, phenol and capsaicinoids, Figure S2: Box plot analysis of percentage of capsaicinoids distribution. Twenty-fifth percentile, median, 75th percentile, and range minimum-maximum. Outliers (white circle) were identified as 1.5 times the interquartile range, Figure S3: Distribution of the qualitative morphological traits throughout the groups of species according to the location (Coast, Andes, Austro, Amazon). Each chart corresponds to a different Capsicum species and each bar to a different region or to the total (last column). The percentages are calculated over the number of plants found to have every type of each character. Different letters at the last column indicate significant differences at *p* < 0.05, Figure S4: Bar plot of the contribution coefficients of each trait to the three principal components.
