# Supplementary material for: Phytochemical Assessment of Native Ecuadorian Peppers (Capsicum spp.) and Correlation Analysis to Fruit Phenomics
Source: Plants (Basel). 2020 Aug 4;9(8):986. doi: 10.3390/plants9080986 (PMC7464142; doi:10.3390/plants9080986)

Table S1. Analysis of variance for biochemical traits among 42 pepper landraces (above). Mean ± SD for each accession (below). Last five columns represent the relative percentage for each capsaicinoids. Last row indicates the average value (Coefficient Variation) for each trait. *Nd* stands for *not detected*. Letters in brackets indicate the *Capsicum* species (A = *C. annuum*, B = *C. baccatu* m, C = *C. chinense*, F = *C. frutescens*, P = *C. pubescens*). Cap = Capsaicin, DHCap = Dihydrocapsaicin, NDHCap = Nordihydrocapsaicin, HCap = Homocapsaicin, HDHCap = Homodihydrocapsaicin.

	**Ascorbic acid**	**Polyphenols**	**Cholesterol**	**Cap**	**DHCap**	**NDHCap**	**Hcap**	**HDHCap**	**Capsaicinoids**	**% Cap**	**% DHCap**	**% NDHCap**	**% Hcap**	**% HDHCap**
Sum of squares	669366.0	3131.64	21036.18	200.64	61.44	3.74	0.17	0.1	471.91					
Mean squares	16326.0	76.38	513.08	4.89	1.50	0.09	0.00	0.0	11.51					
F value[†]	820.7***	73.9***	1.12^{ns}	185.1***	173.8***	129.7***	128.3***	79.1***	188.4***					
BGV11957 (B)	234.72 ± 2.4	20.89 ± 0.4	66.22 ± 4.9	*nd*	*nd*	*nd*	*nd*	*nd*	*nd*	*nd*	*nd*	*nd*	*nd*	*nd*
BGV13300 (P)	209.55 ± 2.3	28.01 ± 0.2	57.62 ± 5.5	0.11 ± 0.0	0.15 ± 0.0	0.09 ± 0.0	*nd*	*nd*	0.39 ± 0.0	28.5	38.3	22.7	10.6	0.0
BGV5852 (B)	128.57 ± 0.7	30.49 ± 0.1	82.43 ± 25.2	1.19 ± 0.3	0.31 ± 0.1	0.10 ± 0.0	*nd*	*nd*	1.60 ± 0.4	74.2	19.6	6.2	0.0	0.0
BGV5857 (A)	293.34 ± 1.6	29.69 ± 0.2	89.48 ± 7.8	*nd*	*nd*	*nd*	*nd*	*nd*	*nd*	*nd*	*nd*	*nd*	*nd*	*nd*
BGV5890 (C)	198.35 ± 4.3	21.23 ± 0.9	91.50 ± 9.1	1.75 ± 0.3	0.60 ± 0.1	*nd*	0.10 ± 0.0	*nd*	2.45 ± 0.3	71.4	24.5	0.0	4.0	0.0
BGV5981 (A)	272.49 ± 4.6	20.48 ± 1.0	78.75 ± 28.4	0.13 ± 0.0	0.10 ± 0.0	*nd*	*nd*	*nd*	0.23 ± 0.0	57.4	42.9	0.0	0.0	0.0
BGV6008 (A)	116.80 ± 2.2	29.67 ± 0.2	74.14 ± 22.3	*nd*	*nd*	*nd*	*nd*	*nd*	*nd*	*nd*	*nd*	*nd*	*nd*	*nd*
BGV6055 (A)	164.30 ± 1.9	19.36 ± 0.4	66.57 ± 34.0	*nd*	*nd*	*nd*	*nd*	*nd*	*nd*	*nd*	*nd*	*nd*	*nd*	*nd*
BGV6064 (B)	139.94 ± 2.4	20.05 ± 0.9	80.62 ± 25.7	0.36 ± 0.0	0.14 ± 0.0	*nd*	*nd*	*nd*	0.50 ± 0.0	72.1	27.9	0.0	0.0	0.0
CGN23259 (C)	183.29 ± 6.6	32.56 ± 0.5	87.08 ± 7.4	*nd*	*nd*	*nd*	*nd*	*nd*	*nd*	*nd*	*nd*	*nd*	*nd*	*nd*
PI224427 (F)	326.75 ± 1.7	33.88 ± 1.3	80.46 ± 33.5	2.69 ± 0.3	1.10 ± 0.1	*nd*	0.14 ± 0.0	*nd*	3.93 ± 0.5	68.4	28.1	0.0	3.6	0.0
PI241670 (A)	422.82 ± 5.2	26.82 ± 0.6	72.68 ± 15.8	1.81 ± 0.2	2.55 ± 0.4	0.59 ± 0.1	*nd*	*nd*	4.95 ± 0.6	36.5	51.6	11.9	0.0	0.0
PI257133 (B)	296.22 ± 1.4	24.82 ± 0.3	81.26 ± 24.6	0.36 ± 0.0	0.29 ± 0.0	0.12 ± 0.0	*nd*	*nd*	0.76 ± 0.0	46.9	37.8	15.3	0.0	0.0
PI257135 (B)	194.84 ± 9.0	15.41 ± 1.6	55.13 ± 28.2	0.55 ± 0.1	0.17 ± 0.0	*nd*	*nd*	*nd*	0.72 ± 0.1	76.0	24.0	0.0	0.0	0.0
PI355394 (P)	125.72 ± 9.2	19.38 ± 0.7	65.97 ± 11.3	0.48 ± 0.0	0.47 ± 0.0	0.16 ± 0.0	*nd*	0.1 ± 0.0	1.19 ± 0.1	40.6	39.3	13.3	0.0	6.9
PI355808 (F)	256.00 ± 6.9	23.28 ± 0.5	55.10 ± 22.1	6.23 ± 0.4	2.35 ± 0.2	*nd*	*nd*	*nd*	8.58 ± 0.6	72.6	27.4	0.0	0.0	0.0
PI355813 (B)	159.28 ± 1.9	20.44 ± 1.3	63.92 ± 14.0	0.30 ± 0.0	0.18 ± 0.0	*nd*	*nd*	*nd*	0.48 ± 0.0	62.5	37.5	0.0	0.0	0.0
PI360725 (C)	116.81 ± 1.5	33.55 ± 1.8	69.24 ± 19.1	4.01 ± 0.5	0.80 ± 0.1	*nd*	*nd*	*nd*	4.81 ± 0.7	83.4	16.6	0.0	0.0	0.0
PI585238 (A)	368.63 ± 5.6	33.66 ± 2.4	86.47 ± 4.6	2.48 ± 0.2	3.28 ± 0.2	0.90 ± 0.1	*nd*	0.2 ± 0.0	6.83 ± 0.5	36.4	48.0	13.1	0.0	2.5
PI585239 (B)	174.54 ± 1.5	18.17 ± 0.1	57.07 ± 26.4	0.15 ± 0.0	0.07 ± 0.0	*nd*	*nd*	*nd*	0.22 ± 0.0	68.1	31.9	0.0	0.0	0.0
PI585241 (B)	196.34 ± 2.0	24.62 ± 0.1	60.11 ± 16.1	0.16 ± 0.0	0.08 ± 0.0	*nd*	*nd*	*nd*	0.23 ± 0.0	67.0	33.2	0.0	0.0	0.0
PI585244 (B)	247.55 ± 0.8	19.92 ± 1.1	62.68 ± 12.6	0.70 ± 0.0	0.18 ± 0.0	0.10 ± 0.0	*nd*	*nd*	1.03 ± 0.1	68.5	18.0	9.3	0.0	4.3
PI585246 (A)	320.46 ± 8.3	23.22 ± 0.3	78.17 ± 7.7	1.07 ± 0.3	0.56 ± 0.2	0.22 ± 0.1	*nd*	*nd*	1.85 ± 0.5	57.8	30.1	12.1	0.0	0.0
PI585249 (B)	185.57 ± 1.6	21.47 ± 2.4	71.46 ± 18.6	0.85 ± 0.1	0.34 ± 0.1	0.10 ± 0.0	*nd*	*nd*	1.29 ± 0.1	66.0	26.4	7.6	0.0	0.0
PI585252 (C)	203.06 ± 1.5	20.80 ± 0.2	74.14 ± 10.5	*nd*	*nd*	*nd*	*nd*	*nd*	*nd*	*nd*	*nd*	*nd*	*nd*	*nd*
PI585253 (C)	302.85 ± 1.5	18.39 ± 0.3	63.61 ± 25.8	0.38 ± 0.1	0.13 ± 0.0	*nd*	*nd*	*nd*	0.51 ± 0.1	74.9	25.0	0.0	0.0	0.0
PI585254 (F)	262.17 ± 4.9	13.83 ± 1.8	52.00 ± 34.5	1.56 ± 0.0	0.50 ± 0.0	*nd*	*nd*	*nd*	2.06 ± 0.0	75.7	24.3	0.0	0.0	0.0
PI585256 (F)	175.54 ± 3.0	21.39 ± 0.8	65.95 ± 27.8	1.73 ± 0.3	0.64 ± 0.1	*nd*	*nd*	*nd*	2.37 ± 0.3	73.0	27.0	0.0	0.0	0.0
PI585257 (F)	248.39 ± 8.2	28.31 ± 1.3	91.43 ± 10.2	2.18 ± 0.0	0.91 ± 0.0	0.31 ± 0.0	*nd*	*nd*	3.40 ± 0.0	64.0	26.9	9.1	0.0	0.0
PI585262 (P)	111.61 ± 1.0	27.95 ± 0.7	72.13 ± 18.1	0.91 ± 0.1	0.75 ± 0.1	0.34 ± 0.0	0.12 ± 0.0	*nd*	2.12 ± 0.2	43.0	35.4	16.0	5.6	0.0
PI585265 (P)	107.37 ± 9.2	19.83 ± 0.3	49.75 ± 28.0	*nd*	*nd*	*nd*	*nd*	*nd*	*nd*	*nd*	*nd*	*nd*	*nd*	*nd*

PI585267 (P)	124.54 ± 7.4	25.42 ± 1.1	57.41 ± 32.4	*nd* ± 0.0	0.10 ± 0.0	*nd*	*nd*	*nd*	0.10 ± 0.0	0.0	100.0	0.0	0.0	0.0
PI585269 (P)	174.38 ± 6.2	21.95 ± 1.8	56.78 ± 27.9	0.25 ± 0.0	0.38 ± 0.0	0.13 ± 0.0	0.09 ± 0.0	0.1 ± 0.0	0.96 ± 0.0	26.3	39.7	14.1	9.7	10.4
PI585275 (P)	143.86 ± 7.2	21.00 ± 1.2	49.12 ± 11.6	0.06 ± 0.0	0.15 ± 0.0	*nd*	*nd*	*nd*	0.22 ± 0.0	29.1	70.7	0.0	0.0	0.0
PI585278 (C)	145.52 ± 1.3	26.41 ± 0.7	89.42 ± 1.9	0.51 ± 0.0	0.16 ± 0.0	*nd*	*nd*	*nd*	0.67 ± 0.1	75.7	24.2	0.0	0.0	0.0
PI593920 (F)	282.05 ± 2.4	32.69 ± 0.8	63.32 ± 23.2	3.40 ± 0.3	1.23 ± 0.1	0.19 ± 0.0	*nd*	*nd*	4.82 ± 0.3	70.5	25.5	4.0	0.0	0.0
PI593922 (C)	220.15 ± 1.0	23.07 ± 0.2	77.84 ± 23.9	1.13 ± 0.1	0.26 ± 0.0	*nd*	*nd*	*nd*	1.40 ± 0.1	81.1	18.9	0.0	0.0	0.0
PI593929 (C)	166.23 ± 1.8	22.90 ± 1.2	79.10 ± 7.2	0.85 ± 0.2	0.69 ± 0.2	0.08 ± 0.0	0.11 ± 0.0	*nd*	1.72 ± 0.4	49.4	40.0	4.4	6.3	0.0
PI593932 (B)	223.62 ± 1.6	23.69 ± 0.8	61.06 ± 14.2	0.47 ± 0.0	0.20 ± 0.0	0.07 ± 0.0	*nd*	*nd*	0.74 ± 0.0	63.9	26.6	9.6	0.0	0.0
PI593933 (C)	217.46 ± 0.9	21.17 ± 0.1	93.15 ± 6.7	0.31 ± 0.1	0.16 ± 0.0	0.07 ± 0.0	*nd*	*nd*	0.53 ± 0.1	57.5	29.8	12.7	0.0	0.0
PI595905 (B)	197.34 ± 1.8	20.76 ± 1.0	57.41 ± 21.7	0.52 ± 0.0	0.21 ± 0.0	0.08 ± 0.0	*nd*	*nd*	0.81 ± 0.0	64.3	26.4	9.4	0.0	0.0
PI595907 (F)	169.59 ± 3.2	25.74 ± 0.4	46.37 ± 27.3	1.50 ± 0.1	0.51 ± 0.0	*nd*	*nd*	*nd*	2.00 ± 0.1	74.8	25.2	0.0	0.0	0.0
**Total**	209.72 (34.9%)	23.96 (21.2%)	69.85 (30.6%)	1.21 (109.0%)	0.59 (124.7%)	0.21 (105.2%)	0.11 (16.9%)	0.12 (38.3%)	1.9 (105.4%)	-	-	-	-	-

† *** Significant at $P < 0.001$

Table S2. Analysis of variance for quantitative conventional descriptors among 42 pepper landraces (above). Mean ± SD for each accession (below). Last row indicates the average value (Coefficient Variation) for each descriptor. Letters in brackets indicate the *Capsicum* species (A = *C. annuum* , B = *C. baccatum* , C = *C. chinense* , F = *C. frutescens* , P = *C. pubescens*). PHE = Plant height, PWI = Plant width, SLE = Stem length, SDI = Stem width, MLL = Mature leaf length, MLW = Mature leaf width, CLE = Corolla length, ALE = Anther length, FILE = Filament length, FLE = Fruit length, FWI = Fruit width, FWE = Fruit weight, FPL = Fruit pedicel length, FWT = Fruit wall thickness, NL = Number of locules.

	PHE	PWI	SLE	SDI	MLL	MLW	CLE	ALE	FILE	FLE	FWI	FWE	FPL	FWT	NL
Sum of squares	173920.53	113976.87	99321.43	2417.21	4093.26	1276.38	52.55	126.88	177.89	3464.73	476.95	37614.48	365.45	579.67	289.33
Mean squares	4241.96	2779.92	2422.47	58.96	99.84	31.13	1.28	3.09	4.34	84.51	11.63	917.43	8.91	14.14	7.06
F value†	2215.32***	1379.10***	3334.55***	363.80***	574.81***	152.35***	19.69***	5.78***	7.80***	2639.65***	827.93***	16599.39***	124.62***	57.57***	27.05***
BGV11957 (B)	67.59 ± 1.59	50.95 ± 1.52	35.60 ± 1.10	9.43 ± 0.42	17.15 ± 0.57	12.08 ± 0.35	1.31 ± 0.09	3.00 ± 0.67	3.00 ± 0.67	9.98 ± 0.24	2.11 ± 0.11	20.68 ± 0.30	3.11 ± 0.21	4.40 ± 0.52	3.60 ± 0.52
BGV13300 (P)	38.96 ± 1.67	61.59 ± 1.53	3.55 ± 0.49	3.51 ± 0.51	8.07 ± 0.62	4.99 ± 0.47	1.95 ± 0.23	3.10 ± 0.99	3.90 ± 0.74	3.06 ± 0.10	2.07 ± 0.12	8.21 ± 0.33	2.49 ± 0.29	5.10 ± 0.57	1.50 ± 0.53
BGV5852 (B)	103.46 ± 1.40	62.47 ± 1.71	5.44 ± 0.75	5.42 ± 0.47	12.01 ± 0.49	6.09 ± 0.43	1.23 ± 0.19	3.00 ± 0.47	5.00 ± 0.82	4.25 ± 0.10	1.58 ± 0.13	5.19 ± 0.21	2.90 ± 0.27	3.10 ± 0.57	2.70 ± 0.48
BGV5857 (A)	75.66 ± 1.50	68.40 ± 1.50	19.01 ± 0.76	4.45 ± 0.36	16.07 ± 0.31	8.10 ± 0.50	1.94 ± 0.36	2.80 ± 0.63	3.90 ± 0.74	10.98 ± 0.19	3.14 ± 0.13	43.53 ± 0.30	3.68 ± 0.25	4.10 ± 0.57	1.60 ± 0.52
BGV5890 (C)	59.00 ± 1.43	92.67 ± 1.26	3.82 ± 1.25	9.48 ± 0.31	12.07 ± 0.34	7.06 ± 0.25	1.88 ± 0.40	3.20 ± 0.63	5.10 ± 0.74	4.78 ± 0.10	4.26 ± 0.11	20.67 ± 0.32	3.11 ± 0.24	6.10 ± 0.74	4.70 ± 0.48
BGV5981 (A)	58.59 ± 0.92	58.78 ± 1.26	13.05 ± 0.74	4.66 ± 0.50	13.09 ± 0.33	6.13 ± 0.52	1.94 ± 0.42	3.30 ± 0.82	4.00 ± 0.82	6.87 ± 0.13	2.98 ± 0.11	24.97 ± 0.27	3.63 ± 0.32	4.10 ± 0.57	3.70 ± 0.48
BGV6008 (A)	62.32 ± 1.65	65.33 ± 1.13	23.53 ± 0.81	4.37 ± 0.27	16.20 ± 0.49	7.10 ± 0.57	1.94 ± 0.23	2.70 ± 0.67	3.90 ± 0.88	3.20 ± 0.12	3.06 ± 0.13	18.19 ± 0.31	3.88 ± 0.21	5.00 ± 0.67	3.70 ± 0.48
BGV6055 (A)	64.03 ± 1.39	65.91 ± 1.35	33.56 ± 0.81	8.64 ± 0.31	15.17 ± 0.62	8.10 ± 0.48	1.94 ± 0.33	2.90 ± 0.74	3.90 ± 0.74	11.66 ± 0.37	3.61 ± 0.10	33.03 ± 0.24	3.95 ± 0.25	4.10 ± 0.32	3.50 ± 0.53
BGV6064 (B)	65.10 ± 1.35	91.72 ± 1.57	4.73 ± 0.61	5.41 ± 0.47	12.09 ± 0.50	7.06 ± 0.33	1.98 ± 0.32	4.00 ± 0.47	4.00 ± 0.67	10.13 ± 0.26	0.59 ± 0.09	20.62 ± 0.23	5.61 ± 0.34	2.30 ± 0.48	3.50 ± 0.53
CGN23259 (C)	55.40 ± 1.49	54.41 ± 1.30	41.08 ± 0.78	7.40 ± 0.24	15.02 ± 0.30	8.05 ± 0.45	1.92 ± 0.36	4.00 ± 0.67	4.00 ± 0.82	5.40 ± 0.12	2.41 ± 0.14	3.23 ± 0.22	3.81 ± 0.20	3.00 ± 0.67	1.70 ± 0.48
PI224427 (F)	102.46 ± 1.06	74.44 ± 1.59	4.34 ± 0.60	8.43 ± 0.36	18.02 ± 0.28	10.07 ± 0.50	1.24 ± 0.16	3.90 ± 0.74	4.00 ± 0.82	5.18 ± 0.10	2.67 ± 0.12	15.89 ± 0.34	5.22 ± 0.26	3.10 ± 0.57	2.60 ± 0.52
PI241670 (A)	64.52 ± 1.58	74.18 ± 1.17	21.49 ± 0.98	9.46 ± 0.32	9.15 ± 0.70	4.97 ± 0.28	1.17 ± 0.13	2.80 ± 0.63	4.00 ± 0.67	9.28 ± 0.25	0.82 ± 0.10	3.29 ± 0.17	2.51 ± 0.27	2.00 ± 0.67	2.60 ± 0.52
PI257133 (B)	36.99 ± 0.96	90.14 ± 1.35	20.88 ± 0.56	6.46 ± 0.33	9.12 ± 0.45	4.05 ± 0.47	1.30 ± 0.18	3.00 ± 0.67	4.10 ± 0.74	3.85 ± 0.14	3.10 ± 0.12	4.75 ± 0.23	2.08 ± 0.33	2.20 ± 0.42	3.80 ± 0.42
PI257135 (B)	42.77 ± 1.27	82.28 ± 1.67	6.25 ± 0.89	7.46 ± 0.33	9.01 ± 0.30	7.00 ± 0.36	1.92 ± 0.43	4.00 ± 0.82	4.90 ± 0.88	8.22 ± 0.27	1.51 ± 0.12	4.96 ± 0.22	3.92 ± 0.20	2.40 ± 0.52	1.60 ± 0.52
PI355394 (P)	43.12 ± 1.23	56.76 ± 1.42	4.39 ± 0.66	3.43 ± 0.59	11.00 ± 0.48	6.11 ± 0.40	1.32 ± 0.19	3.00 ± 0.67	3.90 ± 0.74	6.56 ± 0.10	3.10 ± 0.12	17.33 ± 0.32	2.99 ± 0.26	4.00 ± 0.00	3.70 ± 0.48
PI355808 (F)	87.46 ± 1.17	44.99 ± 1.41	62.22 ± 0.58	4.47 ± 0.60	21.02 ± 0.33	9.95 ± 0.35	1.19 ± 0.15	3.00 ± 0.67	3.00 ± 0.47	2.61 ± 0.10	0.51 ± 0.14	0.50 ± 0.21	2.40 ± 0.27	1.10 ± 0.32	1.50 ± 0.53
PI355813 (B)	38.32 ± 0.94	94.24 ± 1.47	2.74 ± 0.71	10.31 ± 0.25	10.07 ± 0.51	8.06 ± 0.30	1.98 ± 0.33	4.00 ± 0.82	4.10 ± 0.74	2.64 ± 0.11	0.70 ± 0.14	16.52 ± 0.27	3.77 ± 0.18	3.40 ± 0.52	3.50 ± 0.53
PI360725 (C)	83.61 ± 1.46	90.01 ± 1.02	3.35 ± 1.01	9.36 ± 0.45	13.17 ± 0.39	7.05 ± 0.48	1.27 ± 0.23	2.90 ± 0.88	3.00 ± 0.67	3.19 ± 0.12	0.75 ± 0.11	3.76 ± 0.21	1.95 ± 0.28	2.20 ± 0.42	2.60 ± 0.52
PI585238 (A)	95.26 ± 1.08	70.19 ± 1.55	8.49 ± 0.78	3.26 ± 0.35	7.06 ± 0.27	5.03 ± 0.31	1.30 ± 0.19	3.00 ± 0.82	5.00 ± 0.67	5.47 ± 0.14	0.86 ± 0.12	3.69 ± 0.19	2.91 ± 0.22	2.10 ± 0.57	2.40 ± 0.52
PI585239 (B)	87.04 ± 0.92	108.32 ± 1.25	25.72 ± 1.16	3.56 ± 0.68	10.15 ± 0.53	6.13 ± 0.48	1.14 ± 0.16	3.00 ± 0.67	4.00 ± 0.67	8.00 ± 0.24	1.89 ± 0.13	14.92 ± 0.27	4.48 ± 0.29	2.10 ± 0.32	2.60 ± 0.52
PI585241 (B)	102.31 ± 1.60	90.74 ± 1.01	3.35 ± 0.60	8.35 ± 0.31	11.01 ± 0.45	6.07 ± 0.38	1.17 ± 0.17	2.90 ± 0.74	4.00 ± 0.47	4.52 ± 0.12	1.52 ± 0.12	13.19 ± 0.29	3.51 ± 0.24	4.10 ± 0.32	2.60 ± 0.52
PI585244 (B)	53.47 ± 1.81	94.63 ± 1.56	25.04 ± 0.73	9.51 ± 0.38	9.17 ± 0.61	5.09 ± 0.44	1.27 ± 0.13	3.00 ± 0.67	4.00 ± 0.67	3.71 ± 0.11	2.31 ± 0.13	7.78 ± 0.13	4.42 ± 0.26	3.10 ± 0.32	2.60 ± 0.52
PI585246 (A)	84.02 ± 1.46	70.33 ± 1.47	6.52 ± 0.67	4.20 ± 0.18	12.04 ± 0.28	4.14 ± 0.47	1.98 ± 0.30	4.10 ± 0.88	4.00 ± 0.82	10.77 ± 0.35	1.51 ± 0.12	6.66 ± 0.19	5.29 ± 0.31	2.10 ± 0.74	2.10 ± 0.57
PI585249 (B)	57.52 ± 1.33	105.41 ± 1.40	5.33 ± 1.56	4.43 ± 0.32	14.02 ± 0.29	9.15 ± 0.32	1.26 ± 0.25	4.10 ± 0.74	3.90 ± 0.74	9.21 ± 0.28	1.94 ± 0.11	7.83 ± 0.16	2.52 ± 0.20	2.40 ± 0.52	3.70 ± 0.48
PI585252 (C)	70.35 ± 1.51	66.27 ± 1.38	4.54 ± 0.85	8.57 ± 0.34	15.96 ± 0.37	7.13 ± 0.56	1.23 ± 0.09	2.00 ± 0.82	2.90 ± 0.74	8.54 ± 0.30	2.12 ± 0.10	13.28 ± 0.25	5.14 ± 0.23	3.30 ± 0.48	2.50 ± 0.53
PI585253 (C)	102.86 ± 1.02	91.68 ± 1.31	5.36 ± 0.94	10.35 ± 0.41	12.12 ± 0.30	5.11 ± 0.63	1.99 ± 0.46	4.00 ± 0.94	4.00 ± 0.67	4.00 ± 0.09	2.45 ± 0.14	6.36 ± 0.20	3.84 ± 0.32	4.10 ± 0.57	2.50 ± 0.53
PI585254 (F)	92.54 ± 1.43	61.17 ± 1.55	48.61 ± 0.86	8.61 ± 0.40	12.02 ± 0.33	7.12 ± 0.54	1.23 ± 0.16	2.00 ± 0.50	2.11 ± 0.93	2.64 ± 0.07	0.70 ± 0.10	0.30 ± 0.17	2.71 ± 0.35	1.11 ± 0.33	1.67 ± 0.50
PI585256 (F)	63.35 ± 1.50	60.44 ± 1.22	54.62 ± 0.97	4.40 ± 0.32	15.02 ± 0.27	8.07 ± 0.32	1.27 ± 0.15	2.80 ± 0.79	2.00 ± 0.67	1.13 ± 0.12	1.14 ± 0.13	0.67 ± 0.14	2.55 ± 0.31	1.20 ± 0.42	2.40 ± 0.52
PI585257 (F)	64.96 ± 1.44	62.36 ± 1.55	51.51 ± 0.94	10.18 ± 0.41	15.00 ± 0.36	7.14 ± 0.66	1.28 ± 0.18	2.88 ± 0.83	3.88 ± 0.64	1.90 ± 0.13	0.80 ± 0.13	0.26 ± 0.14	1.91 ± 0.25	1.25 ± 0.46	2.50 ± 0.53
PI585262 (P)	24.62 ± 1.37	110.57 ± 1.44	10.18 ± 1.09	4.30 ± 0.30	11.15 ± 0.47	6.09 ± 0.64	1.98 ± 0.23	4.10 ± 0.74	4.10 ± 0.57	1.75 ± 0.10	5.28 ± 0.13	4.08 ± 0.19	2.88 ± 0.26	3.30 ± 0.48	1.70 ± 0.48
PI585265 (P)	64.40 ± 1.48	89.63 ± 1.54	11.23 ± 0.50	3.43 ± 0.34	10.01 ± 0.34	6.02 ± 0.32	1.96 ± 0.24	3.00 ± 0.67	4.10 ± 0.74	4.86 ± 0.11	0.92 ± 0.09	5.41 ± 0.13	3.10 ± 0.18	2.20 ± 0.42	2.50 ± 0.53
PI585267 (P)	58.98 ± 1.53	64.55 ± 1.32	8.52 ± 0.87	3.47 ± 0.51	16.03 ± 0.34	9.08 ± 0.34	1.90 ± 0.32	3.00 ± 0.67	4.00 ± 0.82	3.48 ± 0.12	0.73 ± 0.11	3.93 ± 0.19	2.79 ± 0.24	2.00 ± 0.00	1.70 ± 0.48
PI585269 (P)	56.51 ± 1.31	103.07 ± 1.56	9.58 ± 0.55	3.36 ± 0.33	13.10 ± 0.44	5.07 ± 0.52	1.23 ± 0.23	2.00 ± 0.94	3.00 ± 1.05	4.37 ± 0.13	1.35 ± 0.12	6.35 ± 0.16	3.11 ± 0.23	3.30 ± 0.48	1.70 ± 0.48
PI585275 (P)	54.27 ± 1.44	59.48 ± 1.09	4.41 ± 0.60	4.45 ± 0.67	14.06 ± 0.38	7.10 ± 0.51	1.98 ± 0.40	3.00 ± 0.82	2.90 ± 0.88	5.54 ± 0.13	1.83 ± 0.09	13.09 ± 0.21	4.00 ± 0.22	3.10 ± 0.32	2.60 ± 0.52
PI585278 (C)	64.94 ± 1.30	76.22 ± 1.39	21.57 ± 0.51	4.33 ± 0.41	15.11 ± 0.39	9.14 ± 0.52	1.00 ± 0.00	3.30 ± 0.48	4.40 ± 0.52	3.56 ± 0.15	2.76 ± 0.12	9.51 ± 0.26	3.67 ± 0.21	4.90 ± 0.57	3.60 ± 0.52
PI593920 (F)	109.54 ± 1.40	80.46 ± 1.83	8.31 ± 0.86	3.40 ± 0.24	18.95 ± 0.39	8.10 ± 0.38	1.34 ± 0.19	3.90 ± 0.88	3.90 ± 0.88	2.59 ± 0.13	0.76 ± 0.13	0.34 ± 0.17	2.13 ± 0.33	1.20 ± 0.42	2.60 ± 0.52
PI593922 (C)	75.15 ± 1.52	79.97 ± 1.43	5.80 ± 0.73	5.35 ± 0.51	17.01 ± 0.27	9.08 ± 0.49	1.22 ± 0.13	3.20 ± 0.63	3.00 ± 0.82	5.40 ± 0.08	1.80 ± 0.12	4.78 ± 0.12	2.99 ± 0.27	2.20 ± 0.42	2.60 ± 0.52
PI593929 (C)	49.44 ± 0.99	88.88 ± 1.21	24.47 ± 0.38	5.50 ± 0.35	15.07 ± 0.33	8.06 ± 0.44	1.26 ± 0.14	3.00 ± 0.67	3.90 ± 0.74	8.53 ± 0.25	3.40 ± 0.11	27.00 ± 0.28	2.91 ± 0.37	4.00 ± 0.67	1.60 ± 0.52
PI593932 (B)	71.27 ± 1.15	94.63 ± 1.59	8.64 ± 1.06	8.37 ± 0.39	10.05 ± 0.45	5.07 ± 0.43	1.14 ± 0.23	2.90 ± 0.57	3.90 ± 0.57	8.48 ± 0.32	1.58 ± 0.11	9.76 ± 0.33	3.64 ± 0.30	2.20 ± 0.42	2.50 ± 0.53
PI593933 (C)	47.16 ± 1.21	64.85 ± 1.10	6.48 ± 0.86	5.45 ± 0.31	14.96 ± 0.31	9.02 ± 0.41	1.90 ± 0.31	2.90 ± 0.74	4.00 ± 0.82	3.06 ± 0.13	1.93 ± 0.11	2.51 ± 0.24	2.69 ± 0.31	3.10 ± 0.57	3.50 ± 0.53
PI595905 (B)	62.70 ± 1.69	86.33 ± 1.57	10.39 ± 1.10	9.50 ± 0.37	11.04 ± 0.34	5.14 ± 0.66	1.24 ± 0.21	2.80 ± 0.63	4.00 ± 0.82	6.92 ± 0.13	1.56 ± 0.13	5.22 ± 0.23	4.89 ± 0.27	2.20 ± 0.42	2.60 ± 0.52
PI595907 (F)	87.41 ± 1.71	75.85 ± 1.62	7.41 ± 1.14	5.57 ± 0.32	16.01 ± 0.33	7.98 ± 0.29	1.23 ± 0.15	3.00 ± 0.82	4.10 ± 0.74	1.50 ± 0.09	1.36 ± 0.13	1.07 ± 0.15	2.36 ± 0.24	2.20 ± 0.42	4.50 ± 0.53
**Total**	67.79 (30.2%)	77.13 (21.5%)	16.06 (96.2%)	6.26 (38.9%)	13.17 (23.9%)	7.07 (25.4%)	1.52 (28.3%)	3.15 (28.1%)	3.83 (28.4%)	5.54 (52.4%)	1.94 (55.5%)	10.38 (91.5%)	3.37 (28.8%)	2.97 (42.9%)	2.70 (35.6%)

† *** Significant at $P < 0.001$

Table S3. Analysis of variance for qualitative conventional descriptors among 42 pepper landraces. SCO = Stem colour, NA = Nodal anthocy anin, SSH = Stem shape, SPU = Stem pubescens, PGH = Plant growth habit, BH = Branching habit, TIL = Tillering, LDE = Leaf density, LCO = Leaf colour, LSH = Leaf shape, LMA = Lamina margin, LPU = Leaf pubescens, FAX = Number of flowers per axil, FPO = Flower position, CCO = Corolla colour, CSC = Corolla spot colour, CSH = Corolla shape, ACO = Anther colour, FICO = Filament colour, SEX = Stigma exsertion, CMA = Calyx margin, FCIS = Fruit colour at intermediate stage, FSET = Fruit set, FCMS = Fruit colour at mature stage, FSH = Fruit shape, FSPA = Fruit shape at pedicel attachment, FSBE = Fruit shape at blossom end, FCSC = Fruit cross-sectional corrugation, FSUR = Fruit surface, PLLE = Placenta length.

Trait	Mean squares	F value†
SCO	36.022	33.97***
NA	241.310	85.73***
SSH	6.944	8.69***
SPU	210.754	410.28***
PGH	89.167	53.51***
BH	63.452	67.35***
TIL	7.302	17.16***
LDE	55.873	134.26***
LCO	6.310	15.06***
LSH	2.302	9.99***
LMA	8.452	24.63***
LPU	57.619	60.64***
FAX	9.504	67.52***
FPO	98.730	121.75***
CCO	210.397	308.34***
CSC	141.260	368.67***
CSH	10.456	68.68***
ACO	125.873	323.91***
FICO	353.968	601.72***
SEX	56.587	47.11***
CMA	2.679	64.84***
FCIS	7.629	7.05***
FSET	8.849	12.20***
FCMS	8.929	5.70***
FSH	98.730	55.48***
FSPA	25.655	31.18***
FSBE	37.061	27.75***
FCSC	40.992	40.98***
FSUR	2.232	12.06***
PLLE	3.492	11.24***

† *** Significant at $P < 0.001$

Table S4. Analysis of variance for TA descriptors among 42 pepper landraces (above). Mean ± SD for each accession (below). Last row indicates the average value (Coefficient Variation) for each descriptor. Letters in brackets indicate the *Capsicum* species (A = *C. annuum* , B = *C. baccatum* , C = *C. chinense*, F = *C. frutescens*, P = *C. pubescens*). P = Perimeter A = Area, WMH = Width mid-height, MW = Maximum width, HMW = Height mid-width, MH = Maximum height, CH = Curved height, FSIEI = Fruit shape index external I, FSIEII = Fruit shape index external II, CFSI = Curved fruit shape index, PFB = Proximal fruit blockiness, DFB = Distal fruit blockiness, FST = Fruit shape triangle, E = Ellipsoid, C = Circular, R = Rectangular, SH = Shoulder height, PAMI = Proximal angle micro, PAMA = Proximal angle macro, PIA = Proximal indentation area, DAMI = Distalangle micro, DAMA = Distalangle macro, DIA = Distalindentation area, DEP = Distalend protrusion, Ob = Obovoid, Ov = Ovoid, VAs = V. Asymmetry, HAob = H. Asymmetry. Ob, HAov = H. Asymmetry. Ov, WWP = Width widest position, EC = Eccentricity, PEC = Proximal eccentricity, DEC = Distal eccentricity, FSII = Fruit shape index internal, ECAI = Eccentricity area index, LD = Lobedness degree, TPA = Tomato pericarp area, TPAR = Tomato pericarp area ratio, TPT = Tomato pericarp thickness, TPTR = Tomato oericarp tickness ratio, P = Pepper pericarp boundary.

	P	A	WMH	MW	HMW	MH	CH	FSIEI	FSIEII	CFSI	PFB	DFB	FST	E	C
Sum of squares	6367345.07	948752987.40	93927.66	120922.84	813712.05	1073769.71	1140921.85	1393.01	3923.19	5320.13	38.46	48.90	285.56	1.92	18.40
Mean squares	155301.10	23140316.77	2290.92	2949.34	19846.64	26189.51	27827.36	33.98	95.69	129.76	0.94	1.19	6.96	0.05	0.45
F value[†]	2557.83***	5342.32***	873.28***	954.06***	2320.13***	2567.05***	1828.40***	588.81***	1387.88***	1212.75***	278.35***	171.97***	1182.01***	389.75***	927.50***
BGV11957 (B)	232.01 ± 8.13	2081.36 ± 61.99	27.86 ± 1.88	33.26 ± 1.07	90.40 ± 4.95	97.31 ± 4.53	106.60 ± 5.66	3.935 ± 0.18	5.988 ± 0.12	4.938 ± 0.12	0.501 ± 0.03	1.039 ± 0.11	0.511 ± 0.07	0.087 ± 0.01	0.631 ± 0.05
BGV13300 (P)	93.39 ± 10.14	510.28 ± 105.59	20.49 ± 3.00	22.68 ± 1.68	29.72 ± 3.95	30.56 ± 3.75	32.94 ± 4.26	1.425 ± 0.24	1.430 ± 0.27	1.697 ± 0.14	0.611 ± 0.07	0.841 ± 0.12	0.735 ± 0.12	0.063 ± 0.01	0.136 ± 0.03
BGV5852 (B)	108.87 ± 7.54	618.19 ± 64.81	17.33 ± 1.89	20.00 ± 1.25	39.70 ± 3.19	41.53 ± 3.30	43.48 ± 3.21	2.081 ± 0.18	2.288 ± 0.17	2.478 ± 0.17	0.482 ± 0.04	0.941 ± 0.08	0.496 ± 0.05	0.075 ± 0.01	0.246 ± 0.03
BGV5857 (A)	346.26 ± 11.34	5062.65 ± 94.70	41.83 ± 1.67	49.74 ± 1.36	127.12 ± 1.44	135.34 ± 1.70	143.18 ± 1.66	2.309 ± 0.22	2.964 ± 0.11	3.910 ± 0.12	0.968 ± 0.15	1.112 ± 0.12	1.990 ± 0.01	0.115 ± 0.00	0.339 ± 0.00
BGV5890 (C)	156.99 ± 4.62	1410.68 ± 72.80	40.48 ± 3.12	42.05 ± 2.41	42.57 ± 1.68	44.41 ± 2.85	50.43 ± 2.83	1.029 ± 0.04	1.015 ± 0.02	1.278 ± 0.06	0.529 ± 0.05	0.748 ± 0.04	0.644 ± 0.04	0.066 ± 0.01	0.093 ± 0.01
BGV5981 (A)	316.65 ± 1.70	2286.76 ± 3.95	29.21 ± 1.54	39.65 ± 1.64	65.31 ± 1.28	73.68 ± 1.66	89.33 ± 1.54	2.679 ± 0.24	3.662 ± 0.26	5.398 ± 0.18	0.624 ± 0.00	1.007 ± 0.17	0.672 ± 0.00	0.088 ± 0.01	0.238 ± 0.01
BGV6008 (A)	243.93 ± 1.99	2408.89 ± 24.03	29.41 ± 0.68	38.46 ± 1.00	67.15 ± 0.84	67.65 ± 1.04	80.59 ± 0.87	2.233 ± 0.11	2.655 ± 0.14	2.492 ± 0.20	0.500 ± 0.00	1.186 ± 0.09	0.480 ± 0.01	0.201 ± 0.01	0.275 ± 0.00
BGV6055 (A)	293.37 ± 1.96	3040.43 ± 129.61	30.75 ± 1.04	44.10 ± 0.96	101.20 ± 4.02	119.73 ± 0.37	115.22 ± 6.87	2.971 ± 0.12	3.366 ± 0.27	3.631 ± 0.23	0.979 ± 0.03	0.869 ± 0.10	0.730 ± 0.06	0.104 ± 0.00	0.325 ± 0.01
BGV6064 (B)	208.31 ± 10.91	1259.43 ± 67.21	14.95 ± 1.17	22.01 ± 1.44	78.44 ± 2.73	87.82 ± 3.96	89.06 ± 5.34	4.096 ± 0.23	5.175 ± 0.25	6.325 ± 0.66	0.420 ± 0.04	1.076 ± 0.07	0.390 ± 0.05	0.109 ± 0.01	0.413 ± 0.01
CGN23259 (C)	148.29 ± 13.62	1143.19 ± 86.16	23.78 ± 1.20	24.21 ± 1.03	59.24 ± 1.55	61.57 ± 2.28	59.79 ± 1.51	2.669 ± 0.13	2.833 ± 0.08	2.917 ± 0.13	0.695 ± 0.02	0.793 ± 0.01	0.956 ± 0.06	0.064 ± 0.01	0.289 ± 0.01
PI224427 (F)	133.22 ± 5.04	916.81 ± 41.76	30.36 ± 0.31	30.79 ± 0.61	45.45 ± 0.43	47.08 ± 0.92	50.02 ± 0.83	1.802 ± 0.05	1.728 ± 0.08	1.707 ± 0.04	0.418 ± 0.04	0.712 ± 0.05	0.684 ± 0.05	0.058 ± 0.01	0.182 ± 0.01
PI241670 (A)	214.15 ± 4.57	738.39 ± 26.85	9.37 ± 0.70	23.75 ± 1.75	75.85 ± 2.49	84.77 ± 2.49	92.49 ± 2.26	3.929 ± 0.31	9.305 ± 0.60	10.355 ± 0.62	0.923 ± 0.11	1.125 ± 0.11	0.935 ± 0.07	0.198 ± 0.02	0.424 ± 0.03
PI257133 (B)	105.93 ± 2.16	685.40 ± 8.56	25.82 ± 0.88	28.55 ± 1.45	29.88 ± 1.41	32.25 ± 1.90	35.06 ± 1.71	1.162 ± 0.06	1.203 ± 0.05	1.319 ± 0.13	0.551 ± 0.02	0.819 ± 0.04	0.681 ± 0.07	0.065 ± 0.01	0.081 ± 0.01
PI257135 (B)	170.66 ± 4.19	482.86 ± 2.92	17.43 ± 0.41	21.83 ± 0.95	41.98 ± 1.11	84.96 ± 4.07	82.20 ± 12.55	4.844 ± 0.33	5.081 ± 0.24	5.066 ± 0.92	0.484 ± 0.02	0.821 ± 0.05	0.714 ± 0.05	0.094 ± 0.01	0.405 ± 0.01
PI355394 (P)	71.51 ± 23.86	149.68 ± 18.19	16.96 ± 2.00	18.17 ± 2.36	21.24 ± 3.02	23.30 ± 3.58	24.90 ± 3.66	1.845 ± 0.18	1.948 ± 0.18	2.146 ± 0.19	0.619 ± 0.02	0.805 ± 0.02	0.859 ± 0.02	0.069 ± 0.00	0.221 ± 0.02
PI355808 (F)	58.81 ± 2.58	116.82 ± 6.87	5.53 ± 0.36	6.58 ± 0.33	22.06 ± 1.78	23.36 ± 1.16	23.37 ± 1.19	3.173 ± 0.26	4.099 ± 0.38	4.544 ± 0.24	0.548 ± 0.04	0.857 ± 0.08	0.567 ± 0.03	0.103 ± 0.01	0.383 ± 0.03
PI355813 (B)	62.54 ± 4.68	162.80 ± 11.20	12.43 ± 1.03	11.38 ± 1.02	23.27 ± 2.24	28.82 ± 2.30	27.80 ± 2.75	2.705 ± 0.22	3.047 ± 0.34	3.656 ± 0.43	0.329 ± 0.02	1.114 ± 0.11	0.291 ± 0.03	0.097 ± 0.01	0.330 ± 0.03
PI360725 (C)	99.85 ± 1.73	363.21 ± 14.67	10.26 ± 0.32	15.23 ± 0.38	35.19 ± 0.59	42.75 ± 0.98	50.96 ± 1.70	3.605 ± 0.13	4.095 ± 0.16	4.217 ± 0.12	0.431 ± 0.02	1.167 ± 0.05	0.453 ± 0.04	0.115 ± 0.01	0.371 ± 0.01
PI585238 (A)	159.51 ± 6.61	651.81 ± 50.52	10.37 ± 0.92	26.75 ± 4.43	56.35 ± 6.53	57.77 ± 5.36	65.50 ± 7.73	3.045 ± 0.70	5.565 ± 0.65	7.071 ± 0.68	1.010 ± 0.10	1.160 ± 0.12	0.932 ± 0.11	0.198 ± 0.03	0.394 ± 0.04
PI585239 (B)	163.40 ± 12.70	991.81 ± 13.27	18.28 ± 1.06	22.13 ± 1.36	61.80 ± 3.94	65.80 ± 3.14	67.20 ± 1.55	3.089 ± 0.23	3.308 ± 0.26	3.741 ± 0.23	0.501 ± 0.09	0.872 ± 0.08	0.694 ± 0.09	0.074 ± 0.01	0.347 ± 0.01
PI585241 (B)	218.68 ± 6.90	1702.26 ± 153.94	17.19 ± 0.73	23.71 ± 2.66	93.03 ± 1.52	117.85 ± 3.97	116.22 ± 3.04	4.988 ± 0.55	5.283 ± 0.42	6.840 ± 0.44	0.614 ± 0.07	0.793 ± 0.06	0.615 ± 0.09	0.081 ± 0.01	0.423 ± 0.02
PI585244 (B)	115.88 ± 7.88	801.24 ± 60.78	26.48 ± 2.36	27.91 ± 2.22	35.90 ± 3.19	37.25 ± 3.36	39.82 ± 2.62	1.342 ± 0.12	1.363 ± 0.13	1.501 ± 0.12	0.536 ± 0.06	0.844 ± 0.07	0.707 ± 0.07	0.065 ± 0.01	0.108 ± 0.01
PI585246 (A)	258.36 ± 12.47	1367.81 ± 157.85	14.67 ± 1.34	43.01 ± 1.12	84.12 ± 1.83	105.78 ± 1.63	114.10 ± 1.96	3.622 ± 0.20	6.649 ± 0.17	8.632 ± 0.19	0.987 ± 0.01	1.067 ± 0.19	0.986 ± 0.00	0.218 ± 0.01	0.429 ± 0.01
PI585249 (B)	230.07 ± 6.66	1666.12 ± 63.01	19.84 ± 0.95	33.36 ± 2.59	83.75 ± 4.51	92.92 ± 5.92	97.18 ± 4.73	3.794 ± 0.27	4.132 ± 0.30	4.736 ± 0.30	0.642 ± 0.07	0.989 ± 0.08	0.635 ± 0.10	0.105 ± 0.02	0.394 ± 0.01
PI585252 (C)	177.56 ± 3.26	1409.27 ± 75.11	25.85 ± 2.91	31.51 ± 2.70	63.20 ± 3.36	70.37 ± 3.77	72.48 ± 3.35	2.074 ± 0.23	2.352 ± 0.33	2.801 ± 0.49	0.398 ± 0.07	0.841 ± 0.11	0.597 ± 0.05	0.115 ± 0.02	0.295 ± 0.03
PI585253 (C)	120.68 ± 6.15	721.68 ± 4.34	24.89 ± 3.09	29.25 ± 2.83	41.27 ± 5.50	43.32 ± 5.05	44.55 ± 3.79	1.663 ± 0.09	1.457 ± 0.09	1.468 ± 0.08	0.428 ± 0.07	0.901 ± 0.08	0.486 ± 0.07	0.093 ± 0.01	0.176 ± 0.05
PI585254 (F)	64.04 ± 1.90	153.76 ± 17.52	10.19 ± 0.28	10.95 ± 0.48	21.51 ± 1.17	22.39 ± 1.31	23.32 ± 1.28	1.715 ± 0.16	1.504 ± 0.14	2.496 ± 0.16	0.569 ± 0.02	1.499 ± 0.13	1.575 ± 0.10	0.073 ± 0.01	0.264 ± 0.02
PI585256 (F)	42.09 ± 0.81	103.07 ± 3.64	12.32 ± 0.77	12.52 ± 0.65	9.62 ± 0.72	10.15 ± 0.49	14.93 ± 0.93	0.812 ± 0.05	0.766 ± 0.04	1.011 ± 0.04	0.677 ± 0.05	0.740 ± 0.04	0.989 ± 0.03	0.069 ± 0.01	0.094 ± 0.01
PI585257 (F)	40.24 ± 1.54	70.44 ± 3.97	5.69 ± 0.51	6.43 ± 0.38	15.08 ± 0.73	16.15 ± 0.72	17.19 ± 1.09	2.487 ± 0.09	2.749 ± 0.08	3.066 ± 0.15	0.818 ± 0.08	0.544 ± 0.07	1.968 ± 0.08	0.079 ± 0.01	0.296 ± 0.01
PI585262 (P)	87.26 ± 1.03	393.08 ± 8.85	15.95 ± 1.00	17.47 ± 0.99	29.98 ± 1.23	32.18 ± 1.05	33.97 ± 0.99	1.891 ± 0.02	1.967 ± 0.22	2.177 ± 0.11	0.587 ± 0.00	0.729 ± 0.00	0.840 ± 0.01	0.055 ± 0.00	0.192 ± 0.00
PI585265 (P)	162.88 ± 18.73	747.70 ± 85.37	14.80 ± 1.57	27.58 ± 1.72	63.51 ± 2.15	72.68 ± 1.82	86.77 ± 6.83	3.095 ± 0.25	4.004 ± 0.23	5.022 ± 0.20	1.073 ± 0.09	0.770 ± 0.02	1.651 ± 0.20	0.158 ± 0.02	0.376 ± 0.02
PI585267 (P)	125.18 ± 2.83	595.75 ± 34.36	17.37 ± 1.18	19.53 ± 1.24	40.84 ± 1.24	41.36 ± 0.96	45.37 ± 1.10	2.211 ± 0.08	2.203 ± 0.11	2.545 ± 0.20	0.869 ± 0.01	0.631 ± 0.03	1.397 ± 0.03	0.071 ± 0.01	0.256 ± 0.02
PI585269 (P)	111.54 ± 0.55	765.32 ± 10.57	25.41 ± 1.31	27.48 ± 0.95	27.88 ± 0.67	30.53 ± 2.60	33.33 ± 2.08	0.977 ± 0.05	0.972 ± 0.04	1.089 ± 0.09	0.738 ± 0.07	0.673 ± 0.07	1.018 ± 0.05	0.054 ± 0.01	0.099 ± 0.01
PI585275 (P)	156.16 ± 5.00	1175.97 ± 85.24	26.55 ± 2.33	28.64 ± 0.98	61.44 ± 3.53	60.56 ± 2.48	63.77 ± 2.52	2.988 ± 0.20	2.084 ± 0.17	2.833 ± 0.11	0.540 ± 0.04	0.636 ± 0.05	0.833 ± 0.08	0.077 ± 0.01	0.240 ± 0.05
PI585278 (C)	151.67 ± 2.34	1407.64 ± 22.84	39.23 ± 2.84	37.04 ± 3.04	34.64 ± 2.41	37.73 ± 3.50	43.02 ± 3.47	0.993 ± 0.08	1.018 ± 0.10	1.155 ± 0.12	0.508 ± 0.06	0.926 ± 0.06	0.727 ± 0.05	0.081 ± 0.01	0.088 ± 0.02
PI593920 (F)	58.97 ± 1.57	162.86 ± 0.73	7.45 ± 0.54	8.62 ± 0.50	23.20 ± 1.12	24.19 ± 0.98	25.80 ± 0.91	2.841 ± 0.17	3.095 ± 0.20	3.547 ± 0.19	0.515 ± 0.01	0.876 ± 0.01	0.668 ± 0.01	0.075 ± 0.00	0.329 ± 0.01
PI593922 (C)	159.08 ± 5.57	1059.13 ± 44.18	17.12 ± 0.85	24.70 ± 0.43	61.30 ± 3.59	62.25 ± 4.15	62.00 ± 5.83	2.817 ± 0.39	3.271 ± 0.51	3.859 ± 0.57	0.471 ± 0.07	1.121 ± 0.10	0.483 ± 0.05	0.091 ± 0.01	0.340 ± 0.03
PI593929 (C)	171.47 ± 13.19	1371.03 ± 127.09	32.59 ± 3.55	35.39 ± 3.52	53.58 ± 5.30	56.44 ± 6.33	61.36 ± 4.63	1.570 ± 0.15	1.597 ± 0.15	1.947 ± 0.15	0.532 ± 0.04	0.747 ± 0.08	0.806 ± 0.05	0.096 ± 0.01	0.204 ± 0.02
PI593932 (B)	197.83 ± 12.04	1264.01 ± 103.67	18.29 ± 1.10	23.88 ± 1.50	80.13 ± 1.62	80.79 ± 1.64	82.15 ± 1.62	3.746 ± 0.22	5.139 ± 0.26	6.004 ± 0.23	0.706 ± 0.01	0.932 ± 0.01	0.560 ± 0.01	0.110 ± 0.00	0.412 ± 0.00
PI593933 (C)	82.38 ± 1.00	411.12 ± 7.61	18.17 ± 0.60	22.03 ± 1.20	26.69 ± 1.58	28.06 ± 1.40	29.93 ± 1.52	1.285 ± 0.05	1.356 ± 0.05	1.593 ± 0.08	0.899 ± 0.07	0.350 ± 0.03	2.921 ± 0.27	0.087 ± 0.01	0.120 ± 0.01
PI595905 (B)	158.09 ± 7.92	928.76 ± 119.95	19.09 ± 1.80	24.51 ± 1.79	70.57 ± 5.84	69.61 ± 6.92	71.46 ± 6.45	2.802 ± 0.46	3.025 ± 0.45	4.013 ± 0.61	0.602 ± 0.10	0.835 ± 0.09	0.673 ± 0.07	0.077 ± 0.01	0.319 ± 0.03
PI595907 (F)	43.31 ± 0.40	130.50 ± 3.07	12.78 ± 0.43	13.08 ± 0.37	12.69 ± 0.58	13.01 ± 0.41	15.01 ± 0.71	0.996 ± 0.05	0.993 ± 0.05	1.255 ± 0.06	0.569 ± 0.01	0.799 ± 0.01	0.876 ± 0.00	0.048 ± 0.00	0.056 ± 0.00
**Total**	148.43 (49.6%)	1010.47 (88.7%)	20.24 (44.6%)	25.16 (40.7%)	50.34 (52.3%)	55.61 (54.4%)	59.01 (52.9%)	2.495 (44.4%)	3.018 (60.9%)	3.526 (61.1%)	0.619 (30.7%)	0.891 (24.5%)	0.847 (58.6%)	0.094 (43.9%)	0.275 (46.2%)

Table S4. (*continued*)

	R	SH	PAMI	PAMA	PIA	DAMI	DAMA	DIA	DEP	Ob	Ov	VAs	HAob	HAov	WWP
Sum of squares	4.92	21.99	5278687.48	5674392.08	2934.32	2712325.54	2393709.51	16.98	94.35	21.77	28.63	56.18	381.54	171.49	20.67
Mean squares	0.12	0.54	128748.48	138399.81	71.57	66154.28	58383.16	0.41	2.30	0.53	0.70	1.37	9.31	4.18	0.50
F value[†]	108.91***	223.87***	677.61***	731.94***	3194.60***	366.02***	321.38***	2379.49***	973.73***	284.31***	675.34***	574.52***	915.60***	4936.79***	175.78***
BGV11957 (B)	0.466 ± 0.03	0.492 ± 0.01	120.33 ± 11.41	79.00 ± 5.12	0.950 ± 0.10	124.73 ± 8.49	144.74 ± 14.35	0.006 ± 0.00	0.024 ± 0.01	0.334 ± 0.07	0.000 ± 0.00	0.272 ± 0.04	0.748 ± 0.14	0.000 ± 0.00	0.771 ± 0.06
BGV13300 (P)	0.532 ± 0.03	0.430 ± 0.06	153.11 ± 11.14	118.32 ± 15.48	0.391 ± 0.06	165.77 ± 25.10	138.04 ± 27.33	0.049 ± 0.01	0.078 ± 0.02	0.263 ± 0.06	0.000 ± 0.00	0.084 ± 0.01	0.188 ± 0.02	0.000 ± 0.00	0.621 ± 0.09
BGV5852 (B)	0.474 ± 0.04	0.262 ± 0.02	80.97 ± 7.38	69.98 ± 7.63	0.440 ± 0.04	144.71 ± 10.66	118.67 ± 9.38	0.007 ± 0.00	0.109 ± 0.01	0.395 ± 0.04	0.000 ± 0.00	0.063 ± 0.01	0.370 ± 0.03	0.000 ± 0.00	0.718 ± 0.06
BGV5857 (A)	0.456 ± 0.00	0.492 ± 0.01	296.97 ± 14.80	385.64 ± 5.76	3.502 ± 0.01	83.29 ± 12.00	49.86 ± 1.17	0.040 ± 0.00	0.168 ± 0.01	0.379 ± 0.01	0.550 ± 0.00	0.375 ± 0.00	0.977 ± 0.00	2.197 ± 0.17	0.890 ± 0.00
BGV5890 (C)	0.467 ± 0.05	0.485 ± 0.04	153.55 ± 22.97	121.73 ± 14.07	0.981 ± 0.07	156.78 ± 20.34	148.22 ± 19.08	0.028 ± 0.01	0.069 ± 0.01	0.255 ± 0.05	0.000 ± 0.00	0.141 ± 0.02	0.261 ± 0.04	0.000 ± 0.00	0.555 ± 0.05
BGV5981 (A)	0.481 ± 0.01	0.510 ± 0.01	120.69 ± 19.71	96.55 ± 1.37	0.641 ± 0.00	164.69 ± 20.75	105.53 ± 19.22	0.072 ± 0.01	0.912 ± 0.01	0.490 ± 0.00	0.000 ± 0.00	0.230 ± 0.01	0.570 ± 0.00	0.000 ± 0.00	0.770 ± 0.00
BGV6008 (A)	0.413 ± 0.01	0.550 ± 0.01	105.92 ± 2.52	71.56 ± 1.25	2.429 ± 0.15	123.87 ± 8.60	90.36 ± 10.04	0.045 ± 0.00	0.060 ± 0.01	0.359 ± 0.01	0.577 ± 0.00	0.845 ± 0.00	1.033 ± 0.04	1.052 ± 0.00	0.686 ± 0.01
BGV6055 (A)	0.419 ± 0.03	0.439 ± 0.03	56.63 ± 0.44	50.60 ± 1.12	2.880 ± 0.13	168.28 ± 2.70	106.42 ± 3.41	0.038 ± 0.01	0.069 ± 0.02	0.213 ± 0.14	0.000 ± 0.00	0.542 ± 0.10	0.789 ± 0.08	0.000 ± 0.00	0.776 ± 0.05
BGV6064 (B)	0.395 ± 0.03	0.500 ± 0.00	45.10 ± 2.19	18.49 ± 2.78	0.925 ± 0.14	104.02 ± 8.25	77.32 ± 15.04	0.019 ± 0.00	0.824 ± 0.08	0.472 ± 0.05	0.000 ± 0.00	0.258 ± 0.06	1.018 ± 0.05	0.000 ± 0.00	0.754 ± 0.04
CGN23259 (C)	0.513 ± 0.02	0.494 ± 0.01	129.85 ± 31.09	171.13 ± 16.91	0.608 ± 0.03	267.15 ± 11.15	95.36 ± 15.26	0.048 ± 0.01	0.504 ± 0.02	0.193 ± 0.01	0.194 ± 0.02	0.178 ± 0.02	0.404 ± 0.01	0.393 ± 0.02	0.497 ± 0.01
PI224427 (F)	0.423 ± 0.02	0.392 ± 0.03	51.65 ± 2.35	59.06 ± 3.27	1.810 ± 0.09	201.90 ± 3.09	199.93 ± 5.33	0.045 ± 0.01	0.088 ± 0.01	0.267 ± 0.03	0.000 ± 0.00	0.132 ± 0.01	0.315 ± 0.04	0.000 ± 0.00	0.532 ± 0.04
PI241670 (A)	0.274 ± 0.04	0.487 ± 0.02	303.83 ± 29.78	250.15 ± 56.44	9.053 ± 0.58	88.89 ± 14.93	294.73 ± 28.27	0.044 ± 0.01	0.798 ± 0.09	0.444 ± 0.05	0.000 ± 0.00	0.617 ± 0.07	1.913 ± 0.10	0.000 ± 0.00	0.784 ± 0.06
PI257133 (B)	0.492 ± 0.04	0.503 ± 0.04	120.82 ± 5.59	112.71 ± 7.51	0.200 ± 0.03	128.66 ± 7.14	122.51 ± 4.76	0.018 ± 0.01	0.053 ± 0.01	0.279 ± 0.08	0.000 ± 0.00	0.064 ± 0.01	0.188 ± 0.06	0.000 ± 0.00	0.648 ± 0.04
PI257135 (B)	0.456 ± 0.03	0.495 ± 0.05	50.22 ± 5.28	34.07 ± 4.36	0.489 ± 0.06	94.06 ± 9.95	82.06 ± 9.49	0.035 ± 0.01	0.207 ± 0.05	0.257 ± 0.06	0.000 ± 0.00	0.225 ± 0.11	0.740 ± 0.09	0.000 ± 0.00	0.549 ± 0.10
PI355394 (P)	0.511 ± 0.02	0.499 ± 0.00	140.30 ± 2.86	98.05 ± 12.89	0.972 ± 0.18	121.40 ± 19.38	128.39 ± 16.87	0.041 ± 0.01	0.078 ± 0.01	0.318 ± 0.03	0.000 ± 0.00	0.081 ± 0.01	0.123 ± 0.02	0.000 ± 0.00	0.698 ± 0.06
PI355808 (F)	0.371 ± 0.03	0.092 ± 0.02	58.07 ± 5.27	38.55 ± 2.58	0.091 ± 0.02	101.12 ± 5.68	66.00 ± 2.87	0.023 ± 0.00	0.059 ± 0.01	0.417 ± 0.03	0.000 ± 0.00	0.031 ± 0.00	0.342 ± 0.13	0.000 ± 0.00	0.723 ± 0.04
PI355813 (B)	0.420 ± 0.03	0.266 ± 0.03	30.07 ± 2.78	28.93 ± 2.75	0.360 ± 0.03	152.25 ± 17.02	138.22 ± 12.81	0.051 ± 0.00	0.055 ± 0.00	0.509 ± 0.05	0.000 ± 0.00	0.138 ± 0.01	0.495 ± 0.04	0.000 ± 0.00	0.778 ± 0.06
PI360725 (C)	0.374 ± 0.02	0.493 ± 0.01	65.89 ± 6.79	62.77 ± 2.71	0.282 ± 0.05	130.71 ± 17.03	121.69 ± 10.64	0.081 ± 0.03	0.163 ± 0.04	0.459 ± 0.05	0.000 ± 0.00	0.349 ± 0.03	0.784 ± 0.10	0.000 ± 0.00	0.764 ± 0.05
PI585238 (A)	0.280 ± 0.03	0.491 ± 0.02	96.59 ± 3.58	59.59 ± 15.80	3.886 ± 0.23	107.06 ± 11.32	109.00 ± 11.98	0.004 ± 0.01	0.005 ± 0.01	0.392 ± 0.03	0.004 ± 0.01	0.731 ± 0.08	0.320 ± 0.06	0.000 ± 0.00	0.620 ± 0.09
PI585239 (B)	0.460 ± 0.04	0.492 ± 0.01	80.04 ± 6.58	92.13 ± 9.77	0.787 ± 0.17	102.68 ± 18.26	90.87 ± 11.24	0.008 ± 0.00	0.359 ± 0.06	0.314 ± 0.08	0.000 ± 0.00	0.180 ± 0.03	0.594 ± 0.13	0.000 ± 0.00	0.656 ± 0.07
PI585241 (B)	0.381 ± 0.04	0.487 ± 0.02	28.98 ± 2.58	33.31 ± 2.21	0.552 ± 0.09	100.22 ± 5.84	19.53 ± 1.92	0.005 ± 0.00	0.010 ± 0.00	0.114 ± 0.02	0.000 ± 0.00	0.535 ± 0.08	2.515 ± 0.40	0.000 ± 0.00	0.496 ± 0.06
PI585244 (B)	0.492 ± 0.04	0.485 ± 0.04	112.86 ± 12.45	127.67 ± 13.67	0.452 ± 0.04	119.33 ± 10.30	151.34 ± 15.09	0.010 ± 0.00	0.052 ± 0.00	0.238 ± 0.02	0.000 ± 0.00	0.079 ± 0.01	0.196 ± 0.02	0.000 ± 0.00	0.628 ± 0.05
PI585246 (A)	0.308 ± 0.01	0.499 ± 0.01	129.16 ± 9.54	19.47 ± 1.64	3.568 ± 0.17	104.70 ± 21.15	64.24 ± 1.43	0.014 ± 0.01	0.881 ± 0.01	0.497 ± 0.01	0.000 ± 0.00	0.772 ± 0.22	2.540 ± 0.17	0.000 ± 0.00	0.883 ± 0.00
PI585249 (B)	0.407 ± 0.03	0.499 ± 0.00	323.95 ± 22.59	133.14 ± 11.52	1.691 ± 0.41	123.04 ± 13.03	130.46 ± 18.42	0.026 ± 0.00	0.469 ± 0.05	0.423 ± 0.07	0.000 ± 0.00	0.583 ± 0.04	1.091 ± 0.29	0.000 ± 0.00	0.714 ± 0.06
PI585252 (C)	0.391 ± 0.03	0.368 ± 0.23	116.86 ± 16.31	88.29 ± 11.06	1.055 ± 0.18	102.21 ± 11.45	101.93 ± 10.59	0.006 ± 0.01	0.312 ± 0.04	0.380 ± 0.05	0.000 ± 0.00	0.278 ± 0.06	0.848 ± 0.10	0.000 ± 0.00	0.652 ± 0.07
PI585253 (C)	0.392 ± 0.04	0.422 ± 0.09	144.40 ± 8.82	140.23 ± 8.42	1.353 ± 0.09	146.16 ± 8.61	147.69 ± 5.74	0.169 ± 0.06	0.580 ± 0.07	0.393 ± 0.05	0.000 ± 0.00	0.164 ± 0.05	0.401 ± 0.06	0.000 ± 0.00	0.686 ± 0.04
PI585254 (F)	0.336 ± 0.04	0.487 ± 0.02	163.41 ± 7.73	87.74 ± 3.16	1.336 ± 0.17	1.39 ± 0.22	76.40 ± 1.61	0.000 ± 0.00	0.974 ± 0.22	0.116 ± 0.02	0.144 ± 0.16	0.069 ± 0.01	0.068 ± 0.01	0.133 ± 0.01	0.332 ± 0.03
PI585256 (F)	0.542 ± 0.03	0.513 ± 0.03	163.14 ± 17.47	154.46 ± 6.61	0.313 ± 0.07	271.61 ± 32.78	108.39 ± 14.22	0.063 ± 0.01	0.296 ± 0.03	0.197 ± 0.03	0.173 ± 0.04	0.063 ± 0.01	0.038 ± 0.01	0.052 ± 0.06	0.547 ± 0.05
PI585257 (F)	0.408 ± 0.01	0.480 ± 0.02	183.60 ± 11.91	162.65 ± 10.03	0.284 ± 0.07	114.71 ± 4.90	102.20 ± 10.04	0.017 ± 0.00	0.246 ± 0.09	0.000 ± 0.00	0.282 ± 0.10	0.053 ± 0.01	0.000 ± 0.00	0.151 ± 0.03	0.503 ± 0.07
PI585262 (P)	0.486 ± 0.00	0.497 ± 0.00	112.95 ± 9.95	94.62 ± 13.07	0.578 ± 0.00	132.21 ± 9.28	126.27 ± 12.01	0.769 ± 0.00	0.076 ± 0.00	0.242 ± 0.00	0.000 ± 0.00	0.064 ± 0.00	0.173 ± 0.00	0.000 ± 0.00	0.639 ± 0.00
PI585265 (P)	0.397 ± 0.02	0.499 ± 0.01	151.80 ± 2.88	158.85 ± 1.52	2.042 ± 0.22	101.07 ± 13.30	88.88 ± 1.69	0.038 ± 0.00	0.164 ± 0.02	0.111 ± 0.02	0.530 ± 0.02	0.550 ± 0.02	0.940 ± 0.04	1.110 ± 0.04	0.462 ± 0.01
PI585267 (P)	0.462 ± 0.02	0.478 ± 0.02	157.42 ± 14.26	139.92 ± 18.61	0.234 ± 0.02	122.40 ± 9.31	139.08 ± 26.51	0.010 ± 0.00	0.153 ± 0.03	0.110 ± 0.02	0.244 ± 0.03	0.145 ± 0.02	0.157 ± 0.03	0.227 ± 0.02	0.406 ± 0.02
PI585269 (P)	0.547 ± 0.04	0.105 ± 0.00	168.02 ± 30.10	154.07 ± 10.37	0.946 ± 0.06	139.03 ± 8.42	127.12 ± 16.02	0.002 ± 0.00	0.007 ± 0.00	0.126 ± 0.01	0.124 ± 0.01	0.096 ± 0.01	0.009 ± 0.00	0.018 ± 0.00	0.467 ± 0.09
PI585275 (P)	0.451 ± 0.04	0.466 ± 0.03	103.79 ± 6.32	264.94 ± 36.73	0.837 ± 0.07	121.49 ± 7.46	104.36 ± 3.35	0.027 ± 0.02	0.118 ± 0.03	0.205 ± 0.06	0.189 ± 0.05	0.154 ± 0.02	0.205 ± 0.08	0.092 ± 0.01	0.481 ± 0.05
PI585278 (C)	0.465 ± 0.04	0.000 ± 0.00	149.18 ± 4.85	170.35 ± 18.53	0.000 ± 0.00	120.80 ± 6.26	192.98 ± 9.65	0.000 ± 0.00	0.000 ± 0.00	0.407 ± 0.03	0.000 ± 0.00	0.207 ± 0.04	0.231 ± 0.04	0.000 ± 0.00	0.603 ± 0.04
PI593920 (F)	0.455 ± 0.00	0.494 ± 0.01	64.26 ± 1.52	52.29 ± 1.48	0.060 ± 0.01	103.69 ± 11.32	94.34 ± 14.49	0.014 ± 0.00	0.216 ± 0.01	0.386 ± 0.00	0.000 ± 0.00	0.061 ± 0.01	0.219 ± 0.01	0.000 ± 0.00	0.689 ± 0.01
PI593922 (C)	0.421 ± 0.08	0.468 ± 0.06	55.89 ± 10.08	44.05 ± 6.62	0.792 ± 0.16	197.48 ± 12.82	117.47 ± 15.49	0.062 ± 0.02	0.000 ± 0.00	0.465 ± 0.03	0.000 ± 0.00	0.127 ± 0.02	0.803 ± 0.10	0.000 ± 0.00	0.807 ± 0.08
PI593929 (C)	0.440 ± 0.05	0.481 ± 0.06	130.58 ± 10.83	92.78 ± 7.78	0.704 ± 0.08	134.33 ± 11.41	120.94 ± 10.06	0.019 ± 0.00	0.107 ± 0.01	0.196 ± 0.02	0.053 ± 0.01	0.201 ± 0.01	0.340 ± 0.02	0.081 ± 0.01	0.538 ± 0.05
PI593932 (B)	0.522 ± 0.00	0.500 ± 0.00	68.81 ± 1.36	54.40 ± 1.60	0.740 ± 0.01	108.73 ± 15.00	126.37 ± 16.20	0.040 ± 0.00	0.669 ± 0.01	0.540 ± 0.00	0.000 ± 0.00	0.262 ± 0.01	0.587 ± 0.01	0.000 ± 0.00	0.813 ± 0.00
PI593933 (C)	0.396 ± 0.04	0.408 ± 0.10	136.84 ± 30.97	74.46 ± 8.04	0.313 ± 0.07	62.41 ± 4.12	63.02 ± 3.83	0.006 ± 0.00	0.247 ± 0.04	0.000 ± 0.00	0.440 ± 0.05	0.070 ± 0.01	0.279 ± 0.04	0.320 ± 0.06	0.345 ± 0.04
PI595905 (B)	0.416 ± 0.04	0.485 ± 0.03	71.96 ± 7.10	61.58 ± 4.16	0.655 ± 0.07	74.60 ± 7.08	77.93 ± 7.20	0.021 ± 0.00	0.066 ± 0.01	0.391 ± 0.03	0.000 ± 0.00	0.500 ± 0.04	0.410 ± 0.05	0.000 ± 0.00	0.660 ± 0.08
PI595907 (F)	0.471 ± 0.02	0.000 ± 0.00	151.89 ± 7.39	131.09 ± 10.36	0.000 ± 0.00	150.47 ± 17.12	140.86 ± 17.69	0.017 ± 0.00	0.037 ± 0.01	0.288 ± 0.01	0.000 ± 0.00	0.020 ± 0.00	0.052 ± 0.00	0.000 ± 0.00	0.595 ± 0.02
**Total**	0.436 (16.6%)	0.423 (33.8%)	121.51 (56.0%)	104.31 (67.6%)	1.166 (135.0%)	128.69 (38.5%)	115.72 (40.5%)	0.050 (240.5%)	0.249 (114.7%)	0.309 (45.8%)	0.0765 (206.9%)	0.245 (90.7%)	0.568 (101.29%)	0.1246 (305.9%)	0.637 (22.3%)

Table S4. (*continued*)

	EC	PEC	DEC	FSI	ECAI	LD	TPA	TPAR	TPT	TPTR	PPB
Sum of squares	1.78	8.77	1.22	3345.70	18.01	376828.55	210493646.31	29.24	1652.54	2.59	4695509.88
Mean squares	0.04	0.21	0.03	81.60	0.44	9190.94	5133991.37	0.71	40.31	0.06	114524.63
F value[†]	36.423***	163.75***	52.21***	1292.58***	341.13***	1513.60***	3481.82***	3677.52***	482.58***	145.94***	1797.58***
BGV11957 (B)	0.719 ± 0.06	0.900 ± 0.03	0.884 ± 0.03	4.605 ± 0.40	0.483 ± 0.04	49.89 ± 4.24	926.89 ± 58.67	0.476 ± 0.05	4.300 ± 0.26	0.198 ± 0.02	194.03 ± 5.33
BGV13300 (P)	0.760 ± 0.02	0.890 ± 0.00	0.887 ± 0.01	1.489 ± 0.20	0.436 ± 0.03	11.54 ± 1.52	234.43 ± 20.09	0.445 ± 0.00	2.819 ± 0.28	0.199 ± 0.00	71.42 ± 9.22
BGV5852 (B)	0.765 ± 0.03	0.889 ± 0.01	0.885 ± 0.01	2.312 ± 0.17	0.446 ± 0.05	21.05 ± 2.03	274.97 ± 28.57	0.445 ± 0.00	2.838 ± 0.18	0.208 ± 0.01	84.72 ± 5.88
BGV5857 (A)	0.717 ± 0.00	0.885 ± 0.01	0.887 ± 0.00	2.761 ± 0.02	0.525 ± 0.01	24.94 ± 0.94	2476.87 ± 51.86	0.436 ± 0.01	6.309 ± 0.05	0.196 ± 0.01	334.76 ± 3.73
BGV5890 (C)	0.749 ± 0.03	0.890 ± 0.01	0.886 ± 0.01	1.019 ± 0.04	0.400 ± 0.03	6.38 ± 0.48	600.41 ± 28.55	0.437 ± 0.01	4.443 ± 0.31	0.196 ± 0.01	125.36 ± 3.16
BGV5981 (A)	0.671 ± 0.00	0.882 ± 0.01	0.899 ± 0.01	2.572 ± 0.21	0.490 ± 0.01	35.35 ± 1.10	1139.83 ± 3.58	0.438 ± 0.01	4.714 ± 0.11	0.191 ± 0.00	248.80 ± 1.57
BGV6008 (A)	0.693 ± 0.00	0.893 ± 0.00	0.887 ± 0.00	2.463 ± 0.15	1.139 ± 0.02	24.20 ± 1.10	1130.53 ± 3.09	0.443 ± 0.01	4.661 ± 0.09	0.207 ± 0.01	182.65 ± 1.56
BGV6055 (A)	0.729 ± 0.04	1.000 ± 0.03	0.964 ± 0.03	3.017 ± 0.10	0.498 ± 0.06	29.57 ± 0.65	1410.32 ± 78.69	0.440 ± 0.00	5.331 ± 1.00	0.207 ± 0.06	233.86 ± 5.29
BGV6064 (B)	0.724 ± 0.05	0.930 ± 0.03	0.889 ± 0.01	5.716 ± 0.59	0.536 ± 0.03	58.15 ± 3.70	562.33 ± 58.62	0.443 ± 0.00	2.906 ± 0.21	0.234 ± 0.04	161.11 ± 7.84
CGN23259 (C)	0.769 ± 0.02	0.890 ± 0.01	0.887 ± 0.00	2.518 ± 0.39	0.423 ± 0.02	31.25 ± 1.65	453.01 ± 78.73	0.444 ± 0.00	3.504 ± 0.22	0.199 ± 0.01	113.69 ± 13.47
PI224427 (F)	0.766 ± 0.02	0.892 ± 0.01	0.889 ± 0.00	1.820 ± 0.07	0.360 ± 0.04	21.32 ± 1.59	427.31 ± 15.65	0.444 ± 0.00	3.496 ± 0.18	0.200 ± 0.01	104.45 ± 2.16
PI241670 (A)	0.706 ± 0.05	0.995 ± 0.06	0.944 ± 0.06	7.905 ± 0.51	0.553 ± 0.05	59.50 ± 4.36	339.07 ± 27.04	0.444 ± 0.00	1.783 ± 0.08	0.211 ± 0.01	165.77 ± 5.20
PI257133 (B)	0.773 ± 0.02	0.891 ± 0.00	0.889 ± 0.01	1.146 ± 0.04	0.397 ± 0.02	5.16 ± 0.37	318.29 ± 11.00	0.444 ± 0.00	3.248 ± 0.10	0.198 ± 0.01	81.91 ± 1.52
PI257135 (B)	0.730 ± 0.06	0.907 ± 0.03	0.899 ± 0.02	4.338 ± 0.51	0.428 ± 0.07	60.46 ± 5.89	453.42 ± 86.60	0.445 ± 0.00	2.538 ± 0.38	0.219 ± 0.04	125.85 ± 29.71
PI355394 (P)	0.754 ± 0.03	0.898 ± 0.01	0.885 ± 0.00	2.007 ± 0.25	0.491 ± 0.01	18.64 ± 1.66	661.24 ± 94.55	0.448 ± 0.01	2.489 ± 0.88	0.199 ± 0.01	80.11 ± 16.83
PI355808 (F)	0.741 ± 0.04	0.906 ± 0.03	0.888 ± 0.01	4.245 ± 0.23	0.478 ± 0.05	40.50 ± 2.18	49.12 ± 1.74	0.458 ± 0.00	0.970 ± 0.04	0.225 ± 0.02	41.45 ± 3.53
PI355813 (B)	0.689 ± 0.05	0.905 ± 0.02	0.885 ± 0.00	3.092 ± 0.34	0.500 ± 0.06	33.20 ± 3.80	72.83 ± 6.10	0.447 ± 0.01	1.535 ± 0.15	0.221 ± 0.02	49.07 ± 4.58
PI360725 (C)	0.703 ± 0.04	0.895 ± 0.03	0.889 ± 0.02	4.454 ± 0.27	0.507 ± 0.04	43.09 ± 4.30	180.55 ± 23.57	0.448 ± 0.00	2.076 ± 0.09	0.233 ± 0.03	78.84 ± 3.54
PI585238 (A)	0.567 ± 0.06	1.102 ± 0.11	0.956 ± 0.07	4.057 ± 0.13	0.595 ± 0.05	24.63 ± 0.52	275.05 ± 9.93	0.443 ± 0.00	1.814 ± 0.10	0.219 ± 0.02	118.89 ± 3.08
PI585239 (B)	0.750 ± 0.03	0.900 ± 0.02	0.887 ± 0.01	3.375 ± 0.29	0.448 ± 0.04	38.29 ± 4.05	466.90 ± 54.65	0.444 ± 0.00	3.283 ± 0.23	0.209 ± 0.02	125.05 ± 11.80
PI585241 (B)	0.715 ± 0.06	0.903 ± 0.05	0.909 ± 0.04	5.519 ± 0.25	0.496 ± 0.05	60.78 ± 4.62	593.38 ± 66.16	0.443 ± 0.00	3.031 ± 0.22	0.209 ± 0.03	186.47 ± 7.81
PI585244 (B)	0.770 ± 0.02	0.890 ± 0.01	0.892 ± 0.01	1.363 ± 0.13	0.400 ± 0.03	7.44 ± 0.51	344.82 ± 32.01	0.444 ± 0.00	3.398 ± 0.35	0.200 ± 0.01	90.63 ± 6.22
PI585246 (A)	0.775 ± 0.00	1.467 ± 0.14	0.987 ± 0.00	6.353 ± 0.24	0.670 ± 0.00	53.21 ± 1.52	613.44 ± 2.87	0.442 ± 0.00	2.913 ± 0.02	0.240 ± 0.00	220.97 ± 1.25
PI585249 (B)	0.750 ± 0.03	0.952 ± 0.04	0.897 ± 0.02	4.571 ± 0.37	0.488 ± 0.04	54.70 ± 3.24	720.22 ± 33.74	0.442 ± 0.00	3.745 ± 0.23	0.236 ± 0.03	177.99 ± 10.17
PI585252 (C)	0.741 ± 0.04	0.917 ± 0.04	0.887 ± 0.01	1.981 ± 0.17	0.448 ± 0.05	30.20 ± 3.54	642.76 ± 48.65	0.442 ± 0.00	4.474 ± 0.35	0.489 ± 0.05	154.86 ± 7.37
PI585253 (C)	0.752 ± 0.04	0.885 ± 0.03	0.847 ± 0.07	1.446 ± 0.09	0.421 ± 0.05	11.47 ± 1.07	385.88 ± 40.66	1.444 ± 0.06	3.488 ± 0.26	0.217 ± 0.04	93.35 ± 5.42
PI585254 (F)	0.721 ± 0.06	0.965 ± 0.03	0.770 ± 0.03	1.601 ± 0.31	0.347 ± 0.03	15.64 ± 0.24	69.51 ± 5.26	0.453 ± 0.00	1.398 ± 0.03	0.156 ± 0.02	51.64 ± 1.06
PI585256 (F)	0.747 ± 0.02	0.892 ± 0.00	0.879 ± 0.01	0.792 ± 0.05	0.423 ± 0.02	5.07 ± 0.39	51.54 ± 5.16	0.455 ± 0.00	1.305 ± 0.08	0.199 ± 0.01	31.57 ± 2.31
PI585257 (F)	0.768 ± 0.02	0.896 ± 0.01	0.883 ± 0.02	2.886 ± 0.12	0.457 ± 0.02	27.79 ± 1.90	35.37 ± 0.88	0.460 ± 0.00	0.969 ± 0.02	0.216 ± 0.02	30.71 ± 1.30
PI585262 (P)	0.766 ± 0.00	0.888 ± 0.00	0.883 ± 0.01	1.759 ± 0.15	0.426 ± 0.00	14.24 ± 1.15	182.85 ± 4.00	0.443 ± 0.01	2.331 ± 0.10	0.196 ± 0.01	68.04 ± 1.08
PI585265 (P)	0.731 ± 0.02	0.974 ± 0.01	0.983 ± 0.01	5.048 ± 0.23	0.554 ± 0.02	46.66 ± 2.04	534.76 ± 15.73	0.445 ± 0.00	3.122 ± 0.07	0.216 ± 0.02	131.67 ± 12.19
PI585267 (P)	0.772 ± 0.02	0.894 ± 0.01	0.889 ± 0.01	2.222 ± 0.04	0.419 ± 0.02	21.51 ± 2.05	280.17 ± 23.36	0.440 ± 0.02	2.402 ± 0.27	0.198 ± 0.01	103.46 ± 5.79
PI585269 (P)	0.720 ± 0.05	0.890 ± 0.00	0.886 ± 0.00	0.990 ± 0.17	0.451 ± 0.04	5.46 ± 0.39	292.18 ± 19.62	0.443 ± 0.00	3.560 ± 0.28	0.198 ± 0.01	86.56 ± 4.19
PI585275 (P)	0.758 ± 0.04	0.867 ± 0.05	0.892 ± 0.02	2.002 ± 0.08	0.388 ± 0.05	19.70 ± 3.91	453.62 ± 34.74	0.441 ± 0.01	3.999 ± 0.73	0.200 ± 0.01	120.71 ± 5.85
PI585278 (C)	0.743 ± 0.03	0.895 ± 0.01	0.885 ± 0.01	1.044 ± 0.09	0.440 ± 0.04	6.87 ± 0.27	378.30 ± 20.88	0.443 ± 0.00	4.163 ± 0.07	0.191 ± 0.01	117.12 ± 2.65
PI593920 (F)	0.785 ± 0.00	0.889 ± 0.00	0.885 ± 0.00	3.244 ± 0.18	0.475 ± 0.00	35.45 ± 0.87	69.42 ± 1.33	0.454 ± 0.00	1.280 ± 0.05	0.216 ± 0.00	45.54 ± 1.09
PI593922 (C)	0.742 ± 0.05	0.879 ± 0.06	0.867 ± 0.05	3.213 ± 0.27	0.481 ± 0.05	39.25 ± 1.34	432.33 ± 26.25	0.439 ± 0.03	3.090 ± 0.27	0.206 ± 0.02	122.37 ± 3.96
PI593929 (C)	0.744 ± 0.04	0.894 ± 0.02	0.890 ± 0.01	1.596 ± 0.15	0.403 ± 0.02	13.72 ± 1.11	605.63 ± 48.51	0.442 ± 0.00	4.067 ± 0.37	0.202 ± 0.01	134.15 ± 9.09
PI593932 (B)	0.782 ± 0.01	0.949 ± 0.01	0.903 ± 0.01	5.553 ± 0.28	0.561 ± 0.00	55.72 ± 1.33	616.27 ± 3.66	0.440 ± 0.01	3.652 ± 0.12	0.217 ± 0.01	165.96 ± 1.65
PI593933 (C)	0.755 ± 0.03	0.890 ± 0.01	0.887 ± 0.02	1.391 ± 0.08	0.411 ± 0.04	7.08 ± 0.45	187.35 ± 19.73	0.446 ± 0.00	2.526 ± 0.20	0.228 ± 0.03	65.69 ± 3.01
PI595905 (B)	0.743 ± 0.03	0.918 ± 0.04	0.895 ± 0.01	3.929 ± 0.31	0.427 ± 0.03	36.53 ± 3.22	394.67 ± 37.87	0.444 ± 0.00	2.985 ± 0.23	0.206 ± 0.01	138.74 ± 13.25
PI595907 (F)	0.787 ± 0.00	0.892 ± 0.00	0.883 ± 0.00	0.983 ± 0.00	0.399 ± 0.00	4.37 ± 0.18	58.89 ± 1.79	0.453 ± 0.00	1.521 ± 0.06	0.202 ± 0.00	33.80 ± 0.41
**Total**	0.738 (6.9%)	0.920 (10.1%)	0.892 (4.5%)	2.932 (57.9%)	0.477 (26.8%)	28.60 (62.9%)	471.45 (89.7%)	0.470 (33.5%)	3.031 (40.1%)	0.214 (23.7%)	119.26 (53.2%)

[†] *** Significant at *P*< 0.001

Table S5. Mean and Coefficient of Variation (%) for conventional and digital descriptors in *C. annuum*, *C. chinense* and *C. baccatum* within each geographical region. Different letters within the same column indicate significant differences at $P < 0.05$. Only the parameters showing significant differences among regions are represented. See Tables S2 and S4 for traits´ acronyms.

C. annuum

Region	PHE		PWI		SLE		SDI		MLL		MLW		CLE		ALE	
Andes	63.18 2.7	b	65.62 1.9	b	28.55 18.2	a	6.505 34.0	a	15.685 4.8	a	7.600 9.5	a	1.940 14.2	a	2.800 24.9	b
Austro	76.50 20.0	a	65.79 8.0	b	13.52 32.9	b	4.123 18.0	b	12.073 31.7	b	6.420 21.2	b	1.727 25.9	ab	3.033 25.2	ab
Costa	74.27 13.6	a	72.26 3.3	a	14.01 55.1	b	6.830 39.7	b	10.595 14.8	b	4.555 12.5	c	1.575 30.1	b	3.450 28.9	a
	**FLE**		**FWI**		**FWE**		**FWT**		**NL**		**P**		**A**		**WMH**	
Andes	7.43 58.5	b	3.335 9.1	a	25.61 29.7	a	4.550 15.1	a	3.600 14.0	a	263.70 9.3	a	2661.51 12.1	a	29.94 3.6	a
Austro	7.77 30.7	b	2.327 45.7	b	24.06 68.8	a	3.433 32.2	b	2.567 39.2	b	270.78 30.7	a	2555.65 70.3	a	26.46 49.0	a
Costa	10.03 8.2	a	1.165 31.8	c	4.98 34.9	b	2.050 33.5	c	2.350 25.0	b	232.86 10.2	b	1004.68 32.9	b	11.61 24.4	b
	**MW**		**FSIEI**		**FSIEII**		**CFSI**		**PFB**		**FST**		**E**		**C**	
Andes	40.71 7.3	a	2.528 15.1	b	2.939 13.7	c	2.948 20.4	c	0.691 34.4	c	0.580 22.4	c	0.162 29.8	b	0.295 8.5	c
Austro	38.20 25.6	a	2.695 19.9	b	4.115 28.7	b	5.532 24.4	b	0.862 23.6	b	1.161 48.5	a	0.134 38.0	c	0.323 21.6	b
Costa	31.90 30.5	b	3.799 8.1	a	8.181 17.2	a	9.626 10.3	a	0.950 9.0	a	0.957 6.4	b	0.207 8.5	a	0.426 5.3	a
	**R**		**PAMI**		**PAMA**		**PIA**		**DAMI**		**DAMA**		**DIA**		**DEP**	
Andes	0.415 5.4	a	86.21 28.4	c	63.17 16.5	b	2.609 10.1	b	141.64 16.2	a	96.78 11.7	b	0.042 16.2	a	0.064 25.8	c
Austro	0.403 23.1	a	165.58 53.6	b	171.06 83.8	a	2.638 56.3	b	119.98 31.3	b	89.91 33.0	b	0.039 74.1	ab	0.371 109.0	b
Costa	0.289 11.1	b	229.93 39.2	a	152.56 80.4	a	6.732 41.2	a	95.58 20.2	c	197.21 59.3	a	0.031 55.3	b	0.833 9.2	a
	**Ob**		**Ov**		**VAs**		**HAob**		**HAov**		**WWP**		**EC**		**PEC**	
Andes	0.301 38.3	c	0.346 82.5	a	0.724 22.5	a	0.936 14.3	b	0.631 82.5	a	0.722 7.7	b	0.707 4.5	b	0.936 6.0	b
Austro	0.422 12.7	b	0.167 151.2	b	0.449 49.3	b	0.606 44.5	c	0.664 153.4	a	0.754 16.0	a	0.649 11.0	c	0.960 12.7	b
Costa	0.466 9.4	a	0.000 -	c	0.683 24.7	a	2.178 15.6	a	0.000 -	b	0.826 8.0	a	0.735 6.6	a	1.194 21.5	a
	**DEC**		**FSII**		**ECAI**		**LD**		**TPA**		**TPAR**		**TPT**		**TPTR**	
Andes	0.918 4.5	b	2.685 11.3	c	0.883 36.2	a	26.35 10.7	a	1242.44 11.8	a	0.442 1.0	a	4.929 14.4	a	0.207 16.7	b
Austro	0.916 5.5	b	3.147 21.9	b	0.537 10.2	b	28.46 18.1	b	1242.38 72.0	a	0.439 1.3	b	4.184 44.5	b	0.203 9.7	b
Costa	0.963 5.2	a	7.249 12.1	a	0.602 11.2	b	56.84 8.2	c	455.15 30.4	b	0.443 0.5	a	2.261 25.1	c	0.223 7.0	a

Table S5.*(continued)*

C. chinense								
**Region**	**PWI**	**SLE**	**FWE**	**FPLE**	**NL**	**P**	**A**	**HMW**
Amazonia	81.19 17.55 a	15.70 96.11 a	11.89 84.74 a	2.95 22.23 b	2.64 46.40 b	147.13 17.97 a	1069.45 36.13 a	50.38 20.79 a
Costa	74.76 14.60 b	9.49 75.27 b	7.92 50.85 b	3.84 24.00 a	3.03 24.24 a	132.09 27.66 b	965.31 45.48 b	41.81 34.30 b
	**MH**	**CH**	**FSIEI**	**FSIEII**	**CFSI**	**DFB**	**FST**	**E**
Amazonia	53.48 17.15 a	56.91 11.09 a	2.338 40.49 a	2.562 44.84 a	2.844 40.39 a	0.915 21.86 a	0.668 29.67 b	0.086 23.86 b
Costa	45.25 36.46 b	47.73 34.02 b	1.530 27.54 b	1.574 33.36 b	1.786 38.08 b	0.745 33.64 b	1.207 86.36 a	0.094 20.79 a
	**C**	**R**	**SH**	**PAMI**	**PAMA**	**DAMI**	**DEP**	**Ov**
Amazonia	0.259 39.63 a	0.443 15.01 a	0.484 8.79 a	107.15 40.27 b	98.49 47.05 b	177.29 29.95 a	0.169 105.60 b	0.049 153.37 b
Costa	0.174 48.81 b	0.408 11.38 b	0.315 67.00 b	136.17 16.29 a	115.60 34.40 a	107.22 30.17 b	0.300 69.06 a	0.116 169.70 a
	**VAs**	**HAs**	**WWP**	**FSII**	**ECA**	**LD**	**TPA**	**TPAR**
Amazonia	0.199 41.29 a	0.095 161.35 a	0.632 21.76 a	2.560 48.41 a	0.443 12.39 a	26.74 54.43 a	454.39 35.60 a	0.442 3.19 a
Costa	0.178 50.31 b	0.084 171.60 b	0.570 25.73 b	1.488 23.50 b	0.429 11.06 b	14.27 69.49 b	399.64 42.63 b	0.707 62.73 b
	**TPTR**	**PPB**						
Amazonia	0.207 10.60 b	114.88 18.03 a						
Costa	0.286 44.92 a	107.26 31.72 b						

Table S5.*(continued)*

C. baccatum																
**Region**	**PHE**		**SLE**		**SDI**		**MLL**		**MLW**		**CLE**		**FWE**		**FPL**	
Andes	62.85 35.15	b	13.71 84.32	a	7.56 29.21	b	11.21 22.30	b	6.95 33.19	a	1.505 29.03	a	12.61 50.92	a	3.921 26.98	a
Austro	80.49 29.33	a	5.39 22.17	b	4.93 13.04	c	13.02 8.47	a	7.62 21.15	a	1.245 17.18	b	6.51 20.99	b	2.710 11.22	b
Costa	62.37 14.83	b	16.84 50.23	a	8.94 7.76	a	9.61 7.16	c	5.08 8.34	b	1.205 16.26	b	8.77 11.91	b	4.030 12.00	a
	**NL**		**WMH**		**MW**		**MH**		**CH**		**FSIEI**		**FSIEII**		**CFSI**	
Andes	2.975 28.79	a	18.91 25.9	b	23.17 25.6	b	72.43 40.4	a	73.63 40.5	a	3.440 36.2	a	3.963 38.6	a	4.476 38.4	a
Austro	3.200 21.75	ab	18.59 10.5	b	26.68 26.3	a	67.22 39.2	ab	70.33 38.9	ab	2.937 30.4	b	3.210 29.9	b	3.607 32.3	b
Costa	2.550 20.02	b	22.38 20.2	a	25.90 10.7	a	59.02 37.5	b	60.99 35.2	b	2.544 48.1	b	3.251 58.9	b	3.753 60.7	b
	**PFB**		**DFB**		**FST**		**C**		**R**		**SH**		**PAMI**		**PAMA**	
Andes	0.500 21.4	c	0.918 16.0	ab	0.573 28.7	b	0.362 38.7	a	0.435 11.8	b	0.464 17.6	a	67.11 51.3	c	56.98 57.5	b
Austro	0.562 17.9	b	0.965 8.5	a	0.566 18.3	b	0.320 24.2	a	0.440 11.2	b	0.380 31.6	b	202.46 61.1	a	101.56 32.8	a
Costa	0.621 15.2	a	0.888 7.5	b	0.634 14.0	a	0.260 59.1	b	0.507 6.1	a	0.493 5.4	a	90.83 26.3	b	91.04 41.9	a
	**PIA**		**DAMI**		**DAMA**		**DIA**		**DEP**		**Ob**		**VAs**		**HAs**	
Andes	0.606 43.7	b	109.78 23.0	b	92.85 42.1	c	0.021 75.0	ab	0.204 130.2	b	0.334 39.7	a	0.272 61.9	a	0.841 83.7	a
Austro	1.065 65.2	a	133.87 12.0	a	124.57 12.6	b	0.016 61.7	b	0.289 63.9	ab	0.409 14.4	b	0.323 81.6	a	0.730 57.2	a
Costa	0.596 24.9	b	114.03 12.1	b	138.85 14.4	a	0.025 61.6	a	0.360 86.4	a	0.389 39.4	b	0.171 54.0	b	0.391 50.5	b
	**WWP**		**EC**		**PEC**		**LD**		**TPT**		**TPTR**					
Andes	0.661 17.7	b	0.731 7.1	c	0.907 3.5	b	42.63 42.9	a	2.944 25.1	b	0.212 12.5	b				
Austro	0.716 8.4	a	0.758 4.0	b	0.920 4.6	a	37.88 45.3	ab	3.291 15.2	a	0.222 11.4	a				
Costa	0.721 13.7	a	0.776 2.1	a	0.920 3.3	a	31.58 77.2	b	3.525 8.2	a	0.209 5.0	b				

Table S6 . List of accessions employed in this work. *Na* stands for *not available* . Coordinates and elevation were retrieved from genebanks.

Accession	Species	Region	Province	Canton	Coordinates	Elevation (m)	Source[†]
BGV5857	*C. annuum*	Austro	Loja	Loja	04º13'S, 79º16'W	1496	COMAV
BGV5981	*C. annuum*	Austro	Azuay	Sígsig	03º02'S, 78º47'W	2684	COMAV
BGV6008	*C. annuum*	Andes	Chimborazo	Alausí	*na*	*na*	COMAV
BGV6055	*C. annuum*	Andes	Pichincha	Quito	*na*	*na*	COMAV
PI241670	*C. annuum*	Costa	Guayas	Guayaquil	*na*	*na*	USDA
PI585238	*C. annuum*	Austro	Loja	Gonzanamá	04º13'S, 79º26'W	1700	USDA
PI585246	*C. annuum*	Costa	El Oro	Arenillas	03º46'S, 80º01'W	410	USDA
BGV5852	*C. baccatum*	Austro	Loja	Loja	04º10'S, 79º12'W	1815	COMAV
BGV6064	*C. baccatum*	Andes	Pichincha	Quito	*na*	*na*	COMAV
BGV11957	*C. baccatum*	Andes	Pichincha	Quito	00º13'S, 78º30'W	2763	COMAV
PI257133	*C. baccatum*	Andes	Carchi	Tulcan	00º49'S, 77º43'W	2946	USDA
PI257135	*C. baccatum*	Andes	Cotopaxi	Latacunga	00º55'S, 78º37'W	2812	USDA
PI355813	*C. baccatum*	Andes	Tungurahua	Ambato	01º14'S, 78º37'W	2550	USDA
PI585239	*C. baccatum*	Andes	Cotopaxi	Salcedo	00º55'S, 79º36'W	2600	USDA
PI585241	*C. baccatum*	Andes	Chimborazo	Guamote	01º55'S, 78º42'W	3063	USDA
PI585244	*C. baccatum*	Costa	El Oro	Arenillas	03º46'S, 80º01'W	420	USDA
PI585249	*C. baccatum*	Austro	Loja	Saraguro	03º30'S, 79º22'W	2680	USDA
PI593932	*C. baccatum*	Costa	El Oro	Piñas	03º41'S, 79º41'W	1060	USDA
PI595905	*C. baccatum*	Andes	Imbabura	Cotacachi	16º24'S, 67º31'W	1660	USDA
BGV5890	*C. chinense*	Amazonia	Zamora Chinchipe	Zamora	*na*	*na*	COMAV
CGN17040	*C. chinense*	Amazonia	Sucumbios	Lago Agrio	*na*	500	CGN
CGN23259	*C. chinense*	Amazonia	Esmeraldas	Esmeraldas	*na*	*na*	CGN
PI360725	*C. chinense*	Amazonia	Orellana	Orellana	*na*	500	USDA
PI585252	*C. chinense*	Costa	Manabí	Jipijapa	01º28'S, 80º45'W	20	USDA
PI585253	*C. chinense*	Costa	Manabí	Santa Ana	01º10'S, 80º45'W	90	USDA
PI585278	*C. chinense*	Galapagos	Galápagos	Isabela	00º58'S, 91ºW	5	USDA
PI593922	*C. chinense*	Amazonia	Sucumbios	Cascales	00º02'N, 77º12'W	475	USDA
PI593929	*C. chinense*	Amazonia	Zamora Chinchipe	El Pangui	03º37'S, 78º35'W	885	USDA

PI593933	*C. chinense*	Costa	Manabí	Portoviejo	01º03'S, 80º27'W	15	USDA
PI595908	*C. chinense*	Amazonia	Morona Santiago	Sucúa	02º33'S, 78º09'W	850	USDA
PI224427	*C. frutescens*	*na*	*na*	*na*	*na*	*na*	USDA
PI355808	*C. frutescens*	Costa	Esmeraldas	Quininde	*na*	25	USDA
PI585254	*C. frutescens*	Costa	Manabí	Santa Ana	01º10'S, 80º16'W	100	USDA
PI585256	*C. frutescens*	Costa	El Oro	Arenillas	03º46'S, 80º01'W	410	USDA
PI585257	*C. frutescens*	Austro	Loja	Calvas	04º24'S, 79º28'W	1200	USDA
PI593920	*C. frutescens*	Amazonia	Sucumbios	Cascales	00º02'S, 77º12'W	475	USDA
PI593924	*C. frutescens*	Amazonia	Napo	Tena	01º02'S, 67º34'W	470	USDA
PI595907	*C. frutescens*	Costa	Santo Domingo de los Tsáchilas	Santo Domingo	00º06'S, 79º26'W	360	USDA
BGV13300	*C. pubescens*	Austro	Azuay	San Fernando	*na*	*na*	COMAV
PI355394	*C. pubescens*	Austro	Loja	Catamayo	*na*	2200	USDA
PI585262	*C. pubescens*	Austro	Loja	Gonzanamá	04º13'S, 79º25'W	2045	USDA
PI585264	*C. pubescens*	Austro	Azuay	Sigsig	03º04'S, 78º46'W	2610	USDA
PI585265	*C. pubescens*	Andes	Imbabura	Otavalo	00º10'N, 78º12'W	2780	USDA
PI585267	*C. pubescens*	Austro	Azuay	Oña	03º27'S, 79º09'W	2372	USDA
PI585269	*C. pubescens*	Austro	Loja	Saraguro	04º24'S, 79º28'W	2500	USDA
PI585271	*C. pubescens*	Austro	Loja	Saraguro	03º34'S, 79º18'W	2620	USDA
PI585273	*C. pubescens*	Andes	Carchi	Montufar	00º34'N, 79º48'W	2740	USDA
PI585275	*C. pubescens*	Amazonia	Napo	Quijos	*na*	2400	USDA

[†]COMAV= Instituto de Conservación y Mejora de la Agrodiversidad Valenciana,

CGN= Center for Genetic Resources,

USDA= USDA - ARS - Plant Genetic Resources Conservation Unit

Table S7. List of conventional descriptors [70] and digital traits measured with Tomato Analyzer software [16,17].

Conventional descriptors	Acronym	IPGRI	Units/scale
Stem colour	SCO	7.1.2.2	1=Green, 2=Green with purple stripes, 3=Purple
Nodal anthocyanin	NA	7.1.2.3	1=Green, 3=Light purple, 5=Purple, 7=Dark purple
Stem shape	SSH	7.1.2.4	1=Cylindrical, 2=Angled, 3=Flattened
Stem pubescens	SPU	7.1.2.5	3=Sparse, 5=Intermediate, 7=Dense
Plant height	PHE	7.1.2.6	cm
Plant growth habit	PGH	7.1.2.7	3=Prostrate, 5=Intermediate (compact), 7=Erect
Plant width	PWI	7.1.2.8	cm
Stem length	SLE	7.1.2.9	cm
Stem diameter	SDI	7.1.2.10	cm
Branching habit	BH	7.1.2.11	3=Sparse, 5=Intermediate, 7=Dense
Tillering	TIL	7.1.2.12	3=Sparse, 5=Intermediate, 7=Dense
Leaf density	LDE	7.1.2.13	3=Sparse, 5=Intermediate, 7=Dense
Leaf colour	LCO	7.1.2.14	1=Yellow, 2=Light green, 3=Green, 4=Dark green, 5=Light purple, 6=Purple, 7=Variegated
Leaf shape	LSH	7.1.2.15	1=Deltoid, 2=Ovate, 3=Lanceolate
Lamina margin	LMA	7.1.2.16	1=Entire, 2=Undulate, 3=Ciliate
Leaf pubescens	LPU	7.1.2.17	3=Sparse, 5=Intermediate, 7=Dense
Mature leaf length	MLL	7.1.2.18	cm
Mature leaf width	MLW	7.1.2.19	cm
Number of flowers per axil	FAX	7.2.1.2	1=One, 2=Two, 3=Three or more, 4=Many in bunches
Flower position	FPO	7.2.1.3	3=Pendant, 5=Intermediate, 7=Erect
Corolla colour	CCO	7.2.1.4	1=White, 2=Light yellow, 3=Yellow, 4=yellow-green
Corolla spot colour	CSC	7.2.1.5	0=Absent, 1=White, 2=Yellow, 3=Green yellow, 4=Green, 5=purple
Corolla shape	CSH	7.2.1.6	1=Rotate, 2=Campanulate
Corolla lenght	CLE	7.2.1.7	cm
Anther colour	ACO	7.2.1.8	1=White, 2=Yellow, 3=Light blue, 4=Blue, 5=Purple, 6=Dark purple
Anther lenght	ALE	7.2.1.9	mm
Filament colour	FICO	7.2.1.10	1=White, 2=Yellow, 3=Green, 4=Blue, 5=Light purple, 6=Purple
Filament lenght	FILE	7.2.1.11	mm
Stigma exsertion	SEX	7.2.1.12	3=Inserted, 5=Same level, 7=Exserted
Calyx margin	CMA	7.2.1.15	1=Entire, 2=Intermediate, 3=Dentate
Fruit colour at intermediate stage	FCIS	7.2.2.3	1=White, 2=Yellow, 3=Green, 4=Orange, 5=Purple, 6=Deep purple
Fruit set	FSET	7.2.2.4	3=Low, 5=Intermediate, 7=High
Fruit colour at mature stage	FCMS	7.2.2.6	1=White, 2=Lemon-yellow, 3=Pale orange-yellow, 4=Orange-yellow, 5=Pale orange, 6=Orange,
Fruit shape	FSH	7.2.2.7	1=Elongate, 2=Almost round, 3=Triangular, 4=Campanulate, 5=Blocky
Fruit lenght	FLE	7.2.2.8	cm
Fruit width	FWI	7.2.2.9	cm
Fruit weight	FWE	7.2.2.10	g
Fruit pedicel lenght	FPL	7.2.2.11	cm
Fruit wall tickness	FWT	7.2.2.12	mm
Fruit shape at pedicel attachment	FSPA	7.2.2.13	1=Acute, 2=Obtuse, 3=Truncate, 4=Cordate, 5=Lobate
Fruit shape at blossom end	FSBE	7.2.2.15	1=Pointed, 2=Blunt, 3=Sunken, 4=Sunken and pointed
Fruit cross-sectional corrugation	FCSC	7.2.2.17	3=Slightly corrugated, 5=Intermediate, 7=Corrugated
Number of locules	NL	7.2.2.18	-
Fruit surface	FSUR	7.2.2.19	1=Smooth, 2=semi wrinkled, 3=Wrinkled
Placenta length	PLLE	7.2.2.21	1=<1/4 fruit length, 2=1/4-1/2 fruit length, 3= >1/2 fruit length

Tomato Analyzer Descriptors:		
**Basic Measurements:**		
Perimeter	P	mm
Area	A	mm^2
Width mid-height	WMH	mm
Maximum width	MW	mm
Height mid-width	HMW	mm
Maximum height	MH	mm
Curved height	CH	mm
**Fruit Shape Index:**		
Fruit shape index external I	FSIEI	
Fruit shape index external II	FSIEII	
Curved fruit shape index	CFSI	
**Blockiness:**		
Proximal fruit blockiness	PFB	
Distal fruit blockiness	DFB	
Fruit shape triangle	FST	
**Homogeneity:**		
Ellipsoid	E	
Circular	C	
Rectangular	R	
**Proximal Fruit End Shape**		
Shoulder height	SH	
Proximal angle micro	PAMI	Degrees
Proximal angle macro	PAMA	Degrees
Proximal indentation area	PIA	
**Distal Fruit End Shape**:		
Distal angle micro	DAMI	Degrees
Distal angle macro	DAMA	Degrees
Distal indentation area	DIA	
Distal end protrusion	DEP	
**Asymmetry:**		
Obovoid	Ob	
Ovoid	Ov	
V.Asymmetry	VAs	
H.Asymmetry.Ob	HAob	
H.Asymmetry.Ov	HAov	
Width widest position	WWP	
**Internal Eccentricity:**		
Eccentricity	EC	
Proximal eccentricity	PEC	
Distal eccentricity	DEC	
Fruit shape index internal	FSII	
Eccentricity area index	ECA	
**Lattitudinal Section:**		
Lobedness degree	LD	
Tomato pericarp area	TPA	
Tomato pericarp area ratio	TPAR	
Tomato pericarp thickness	TPT	
Tomato oericarp tickness ratio	TPTR	
Pepper pericarp boundary	PPB	

Figure S1. Chemical structure of ascorbic acid, phenol and capsaicinoids

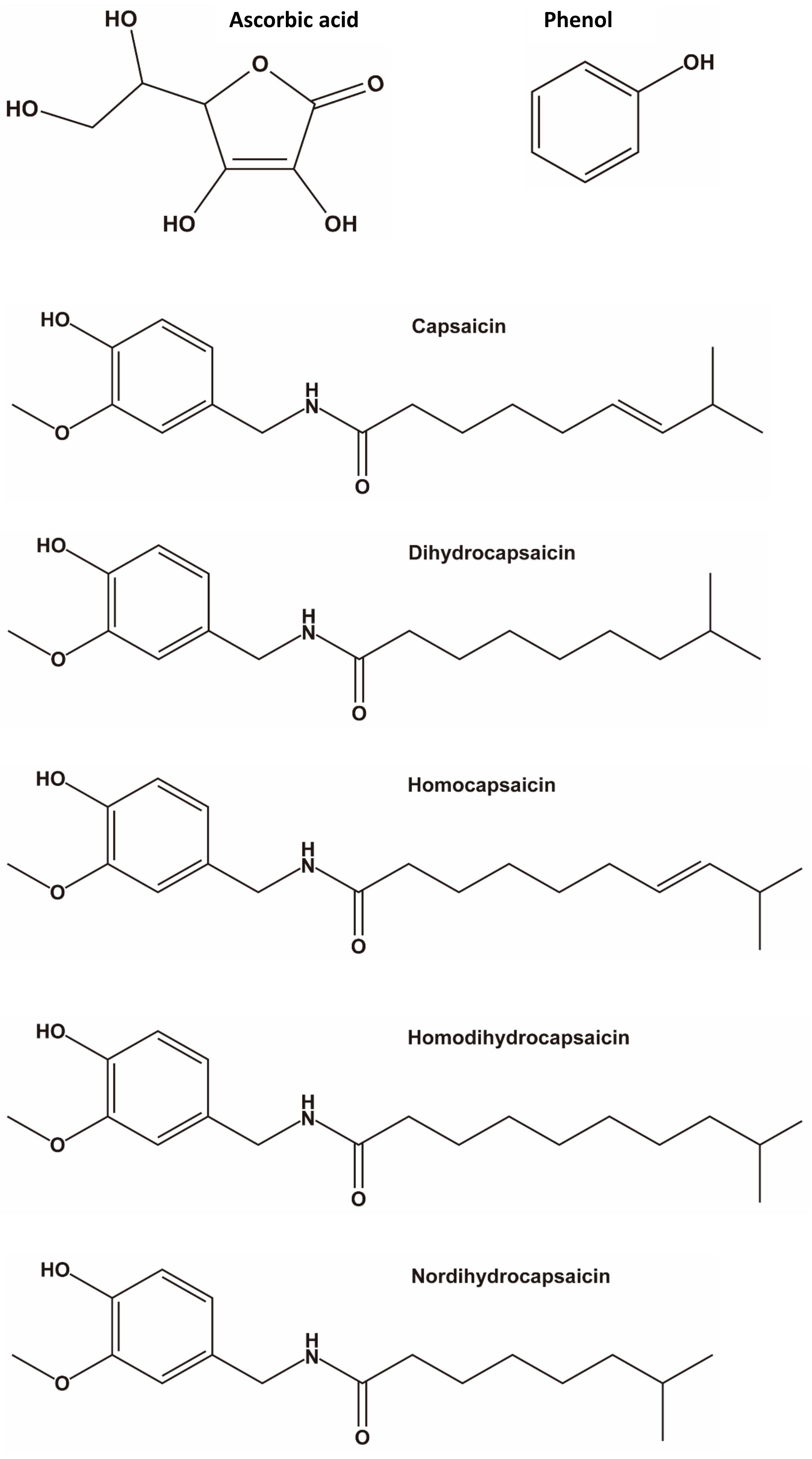

Figure S2. Box plot analysis of percentage of capsaicinoid distribution. Twenty-fifth percentile, median (thick line), 75th percentile, and range minimum-maximum. Outliers (white circle) ere identified as 1.5 times the interquartile range.

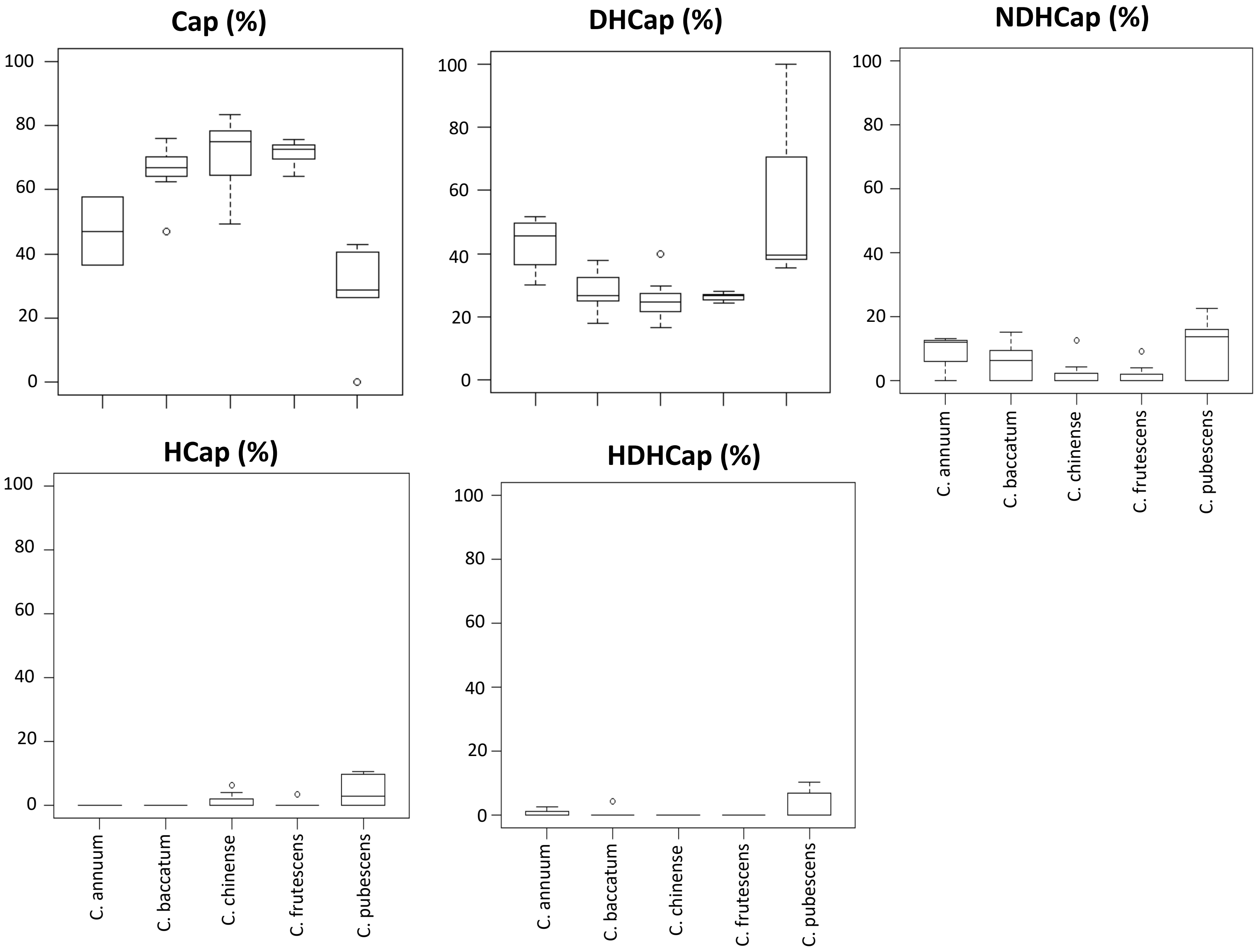

Figure S3. Distribution of the qualitative morphological traits throughout the groups of species according to the location (Coast, Andes, Austro, Amazon). Each chart corresponds to a different character and each bar to a different region. The percentages are calculated over the number of plants found to have every type of each character. Different letters at the last column indicate significant differences at $P < 0.05$.

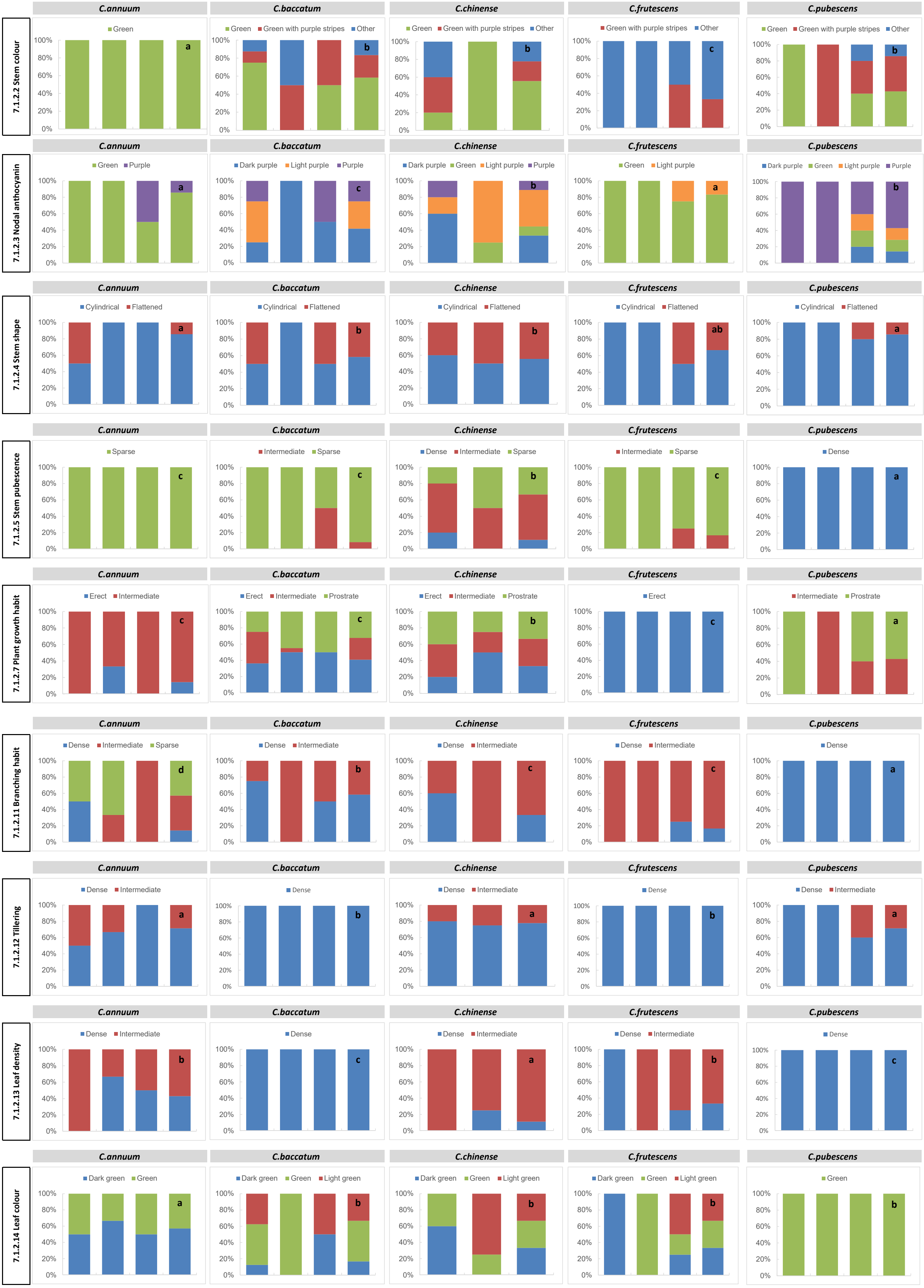

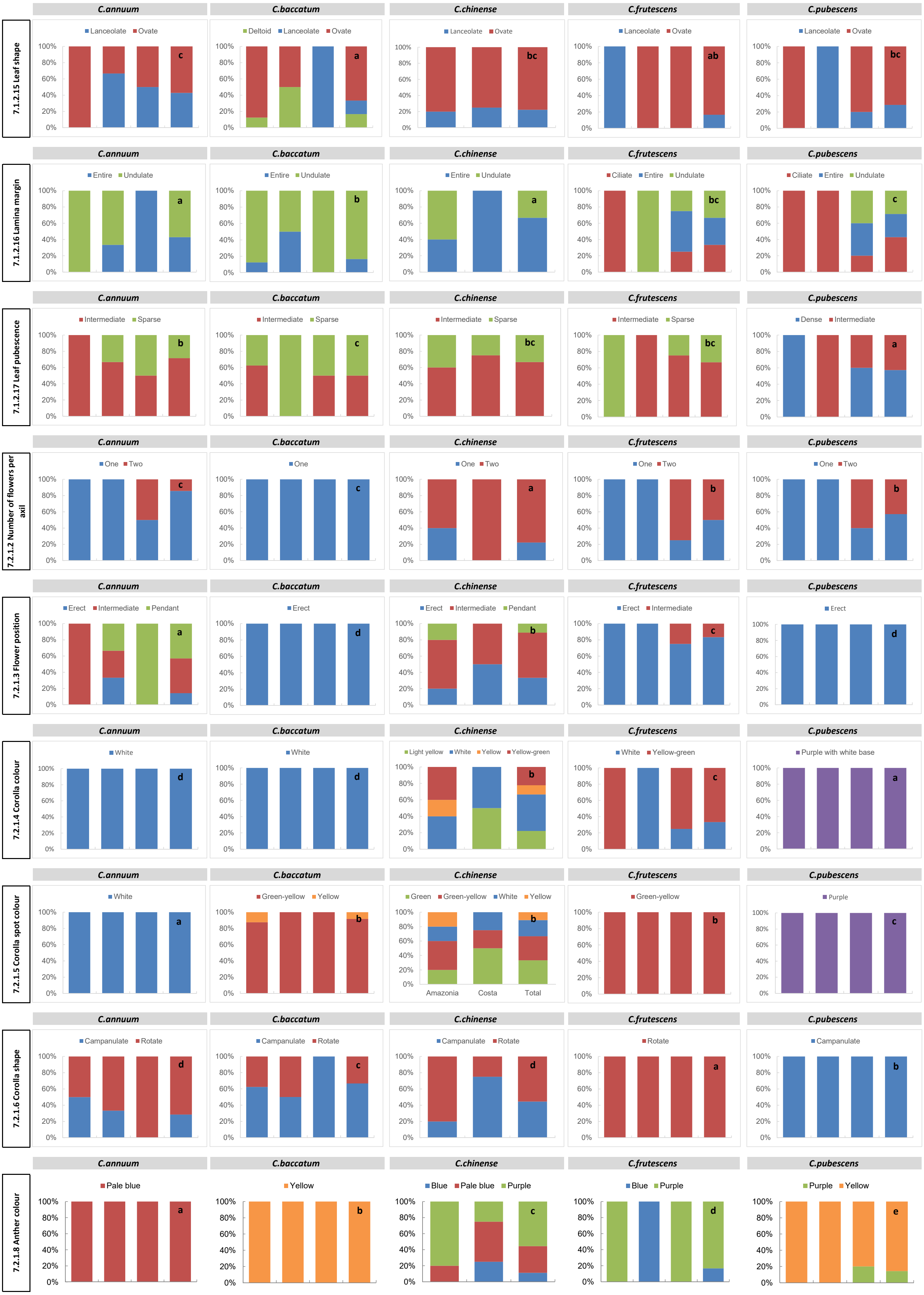
C.annuum
C.baccatum
C.chinense
C.frutescens
C.pubescens
7.1.2.15 Leaf shape
Lanceolate
Ovate
Deltoid
7.1.2.16 Lamina margin
Entire
Undulate
Ciliate
7.1.2.17 Leaf pubescence
Intermediate
Sparse
Dense
7.2.1.2 Number of flowers per axil
One
Two
7.2.1.3 Flower position
Erect
Intermediate
Pendant
7.2.1.4 Corolla colour
White
Light yellow
Yellow
Yellow-green
Purple with white base
7.2.1.5 Corolla spot colour
Green-yellow
Yellow
Green
White
Purple
Amazonia
Costa
Total
7.2.1.6 Corolla shape
Campanulate
Rotate
7.2.1.8 Anther colour
Pale blue
Yellow
Blue
Purple
0%
20%
40%
60%
80%
100%

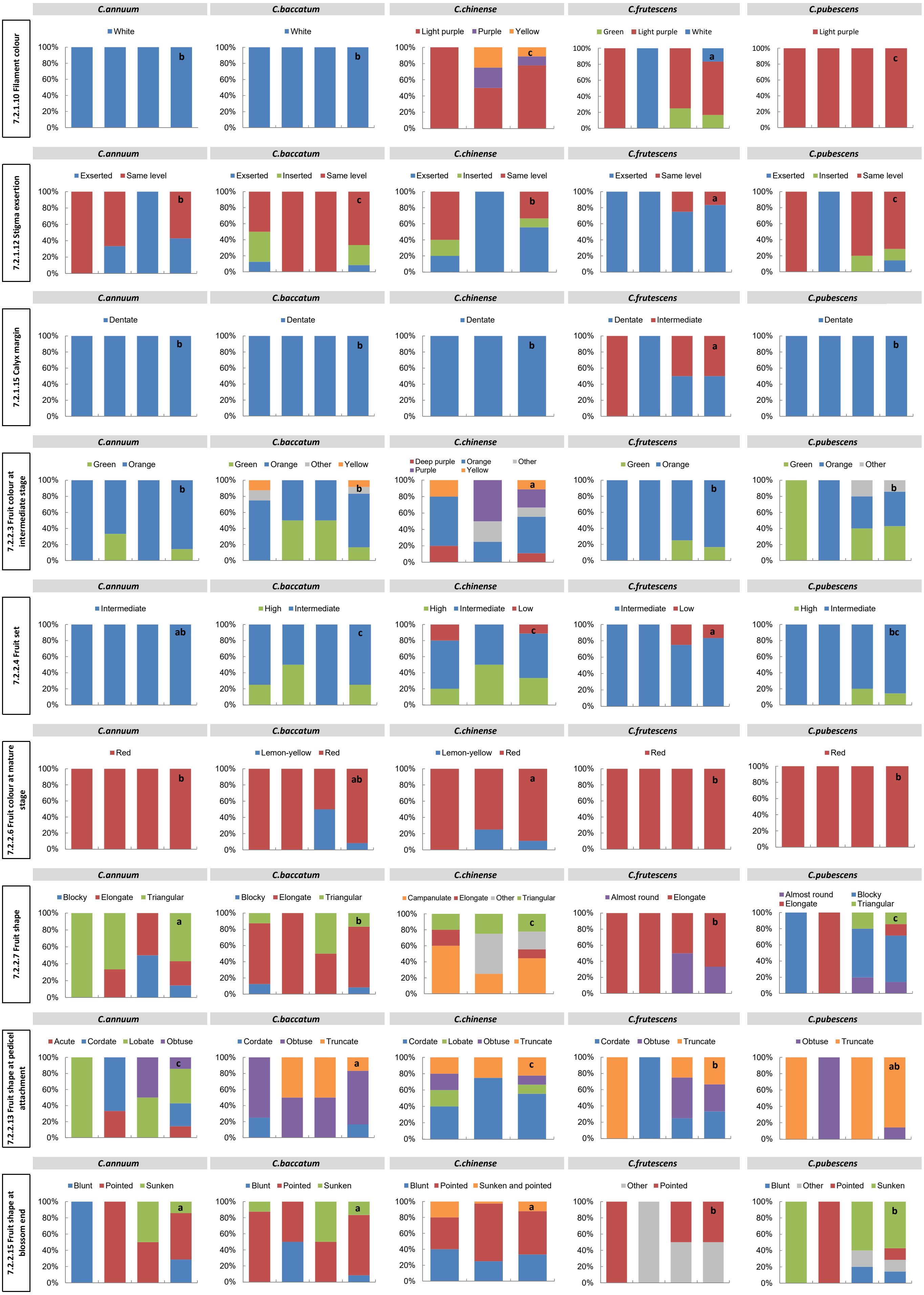
C.annuum
C.baccatum
C.chinense
C.frutescens
C.pubescens
100%
80%
60%
40%
20%
0%
7.2.1.10 Filament colour
White
b
White
b
Light purple
Purple
Yellow
c
Green
Light purple
White
a
Light purple
c
7.2.1.12 Stigma exsertion
Exserted
Same level
b
Exserted
Inserted
Same level
c
Exserted
Inserted
Same level
b
Exserted
Same level
a
Exserted
Inserted
Same level
c
7.2.1.15 Calyx margin
Dentate
b
Dentate
b
Dentate
b
Dentate
Intermediate
a
Dentate
b
7.2.2.3 Fruit colour at intermediate stage
Green
Orange
b
Green
Orange
Other
Yellow
b
Deep purple
Orange
Other
Purple
Yellow
a
Green
Orange
b
Green
Orange
Other
b
7.2.2.4 Fruit set
Intermediate
ab
High
Intermediate
c
High
Intermediate
Low
c
Intermediate
Low
a
High
Intermediate
bc
7.2.2.6 Fruit colour at mature stage
Red
b
Lemon-yellow
Red
ab
Lemon-yellow
Red
a
Red
b
Red
b
7.2.2.7 Fruit shape
Blocky
Elongate
Triangular
a
Blocky
Elongate
Triangular
b
Campanulate
Elongate
Other
Triangular
c
Almost round
Elongate
b
Almost round
Blocky
Elongate
Triangular
c
7.2.2.13 Fruit shape at pedicel attachment
Acute
Cordate
Lobate
Obtuse
c
Cordate
Obtuse
Truncate
a
Cordate
Lobate
Obtuse
Truncate
c
Cordate
Obtuse
Truncate
b
Obtuse
Truncate
ab
7.2.2.15 Fruit shape at blossom end
Blunt
Pointed
Sunken
a
Blunt
Pointed
Sunken
a
Blunt
Pointed
Sunken and pointed
a
Other
Pointed
b
Blunt
Other
Pointed
Sunken
b

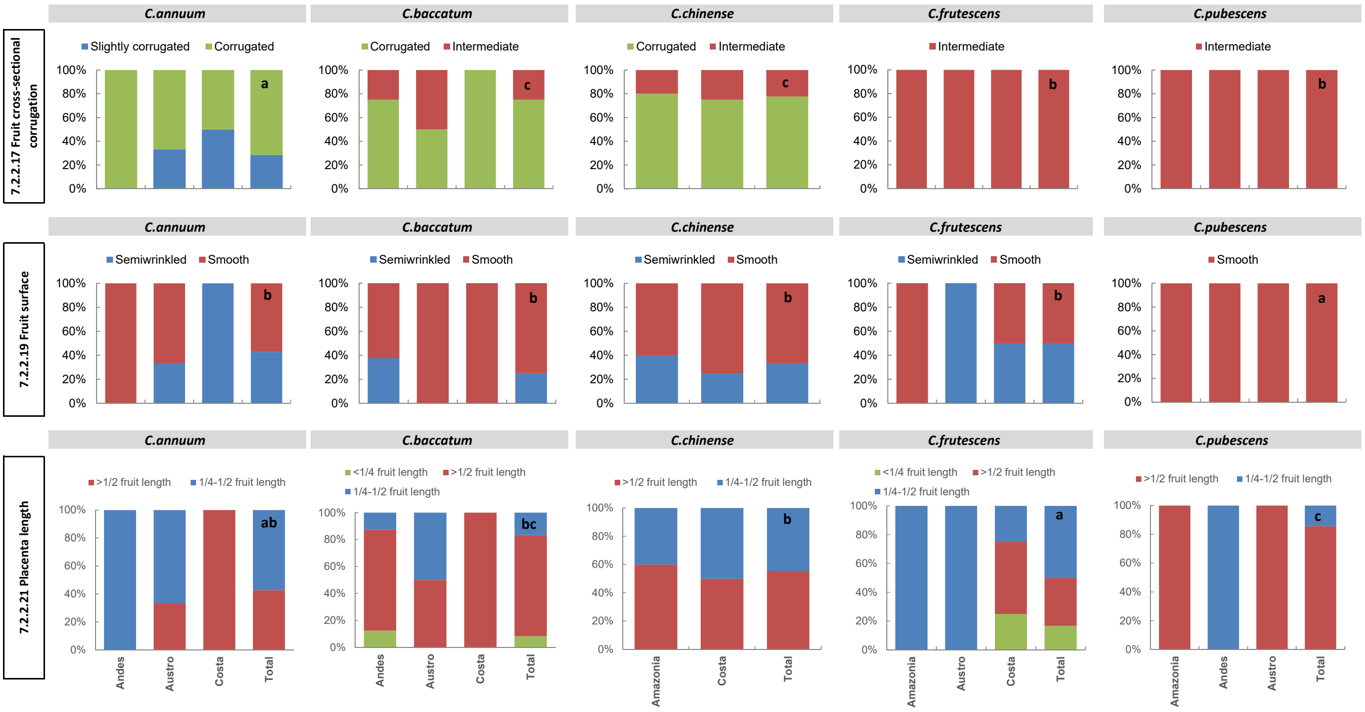

7.2.2.17 Fruit cross-sectional corrugation
7.2.2.19 Fruit surface
7.2.2.21 Placenta length
C.annuum
C.baccatum
C.chinense
C.frutescens
C.pubescens
Slightly corrugated
Corrugated
Intermediate
Semiwrinkled
Smooth
>1/2 fruit length
1/4-1/2 fruit length
<1/4 fruit length
100%
80%
60%
40%
20%
0%
a
b
c
ab
bc
Andes
Austro
Costa
Amazonia
Total

Figure S4. Bar plot of the contribution coefficients of each trait to the three principal components.

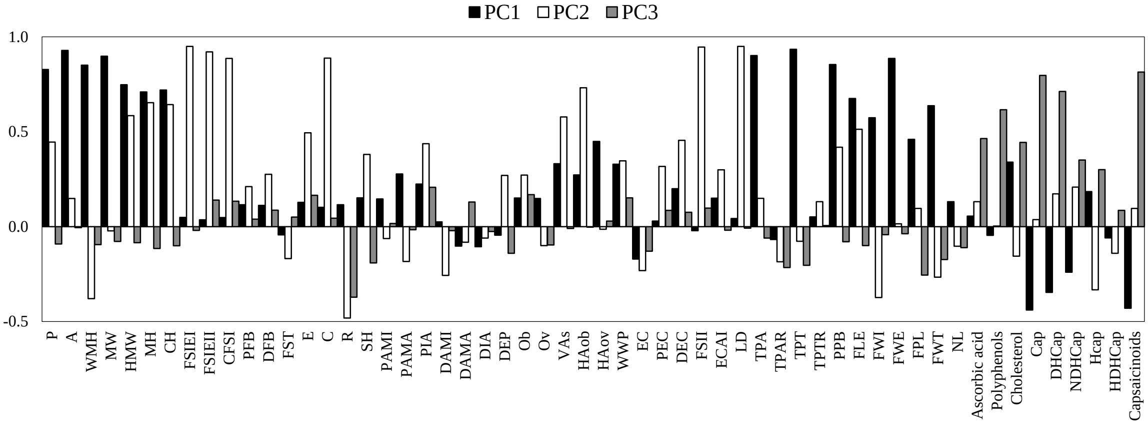

Supplement: Supplementary file 1 [file plants-09-00986-s001.pdf]
